# Integrating Hydrogen Deuterium Exchange–Mass Spectrometry with Molecular Simulations Enables Quantification of the Conformational Populations of the Sugar Transporter XylE

**DOI:** 10.1021/jacs.2c06148

**Published:** 2023-03-28

**Authors:** Ruyu Jia, Richard T. Bradshaw, Valeria Calvaresi, Argyris Politis

**Affiliations:** † Department of Chemistry, King’s College London, 7 Trinity Street, London SE1 1DB, U.K.; ‡ Faculty of Biology, Medicine and Health, School of Biological Sciences, The University of Manchester, Manchester M13 9PT, U.K.; § Manchester Institute of Biotechnology, University of Manchester, Princess Street, Manchester M1 7DN, U.K.

## Abstract

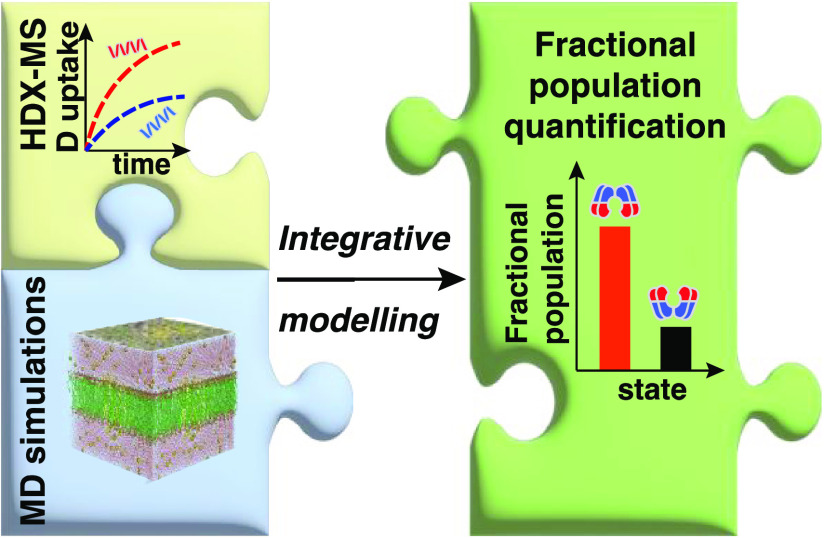

A yet unresolved challenge in structural biology is to quantify the conformational states of proteins underpinning function. This challenge is particularly acute for membrane proteins owing to the difficulties in stabilizing them for in vitro studies. To address this challenge, we present an integrative strategy that combines hydrogen deuterium exchange–mass spectrometry (HDX-MS) with ensemble modeling. We benchmark our strategy on wild-type and mutant conformers of XylE, a prototypical member of the ubiquitous Major Facilitator Superfamily (MFS) of transporters. Next, we apply our strategy to quantify conformational ensembles of XylE embedded in different lipid environments. Further application of our integrative strategy to substrate-bound and inhibitor-bound ensembles allowed us to unravel protein–ligand interactions contributing to the alternating access mechanism of secondary transport in atomistic detail. Overall, our study highlights the potential of integrative HDX-MS modeling to capture, accurately quantify, and subsequently visualize co-populated states of membrane proteins in association with mutations and diverse substrates and inhibitors.

## Introduction

One of the challenges of structural biology in the upcoming decades is to evolve from assigning static structural snapshots to characterizing dynamical ensembles. Novel methodologies are required for the interrogation of membrane proteins, where existing tools, such as crystallography and cryogenic electron microscopy (cryo-EM), are often unsuccessful in probing the intricate interplay of protein and membrane dynamics underlying function. Furthermore, nuclear magnetic resonance (NMR) spectroscopy studies of membrane-bound systems are exceptionally challenging since difficulties in protein expression, stabilization, and the size limitation of the NMR technique often remain prohibitive. In contrast, hydrogen deuterium exchange–mass spectrometry (HDX-MS) is a well-established technique to probe the conformational dynamics of soluble proteins, which has recently emerged also for interrogating more complex protein systems,^1^ such as membrane proteins.^2,3^ HDX-MS reports upon the deuterium exchange rates of backbone amide groups, averaged over oligopeptide protein segments. This technique offers advantages over traditional structural approaches, namely, tolerance for complex, heterogeneous environments (e.g., lipids, detergents, native membranes), low sample requirements, and no need for bio-orthogonal labels. Importantly, HDX-MS data report on the equilibrium of protein conformational ensembles including all relevant populations. Therefore, HDX-MS has become a particularly powerful tool to study dynamical mechanisms inaccessible to other structural techniques, also in systems such as membrane proteins.

To infer structural information from HDX-MS data, the peptide-level exchange has often been unsophisticatedly correlated to molecular simulations,^4^ to give qualitative insights into regions of relative flexibility.^5^ However, these simple insights neglect the full structural details of the information that HDX-MS can provide, whereas the magnitude of dynamical changes that computational interpretations can study is not well understood.^6−9^ The simplicity of these analyses has in the past constrained HDX-MS to qualitative studies, complementary to more technically challenging higher-resolution methods. More advanced HDX-MS analyses instead promise to uncover information that existing crystallographic or solution structural techniques are unable to provide—the full range of conformational states underpinning function in atomistic detail.^5,10^

A key issue in the interpretation of HDX-MS data, however, is the fact that the measured values of deuterium (D) uptake over time represents a conformationally averaged exchange rate for the peptide of interest. Attempts to connect the observed HDX to structure (e.g., a single crystal structure, or single modeled conformational state) are therefore qualitative at best. They also risk being subjective, based on the availability of structural data or preconceived ideas of protein conformational states, rather than an objective selection of a model that best fits the HDX data. The structural context of HDX-MS data is, therefore, better represented as an ensemble of structures, such as those generated by the HDXer method,^11^ or the Bayesian^12^ approach employed in HDX data interpretation.^13^ In this study, HDXer, a python package developed by Bradshaw et al.^11^ was used to recreate HDX-MS experimental observables by computing HDX-MS data from biomolecular simulations and performing ensemble refinement to fit a structural ensemble to experimental data. Although a recent addition to the catalogue of HDX-MS structural analysis software, the HDXer approach has been used to investigate a variety of protein systems, including both soluble and integral membrane proteins.^11,14,15^

Nevertheless, the ensemble-averaged nature of HDX-MS data poses challenges, as well as advantages, for interpretation via ensemble reweighting methods. In particular, if the HDX-MS experiment probes a highly diverse conformational ensemble, i.e., one interconverting between multiple conformational states, each with independent H–D exchange rates, then the ensemble-averaged HDX-MS signal may not be sufficient to unambiguously deconvolute the full extent of structural variation present.^16^ In studies of conformational mechanisms, this can be avoided by restricting proteins to particular conformational states as much as possible. In our own work, we have made use of single point mutations and ligand binding to probe single states of the XylE transport cycle independently of one another and describe the conformational process and key determinants of transport.^2,17^

XylE, an *Escherichia coli* homologue of GLUT1 (human glucose transporter), is one of the closest and most characterized GLUT1 homologues. GLUT1 facilitates glucose translocation across cell membranes in mammalian cells, which is also a promising and valuable drug target as malfunction of GLUT1 is associated with cancer, diabetes, and other diseases. Due to a highly conserved substrate-binding site, many studies have attempted to provide a structural framework to infer the molecular mechanism of sugar transport and crucial ligand interactions with disease-related residues in GLUT1 using XylE.^18−20^ Here, we demonstrate an integrative approach to describe conformational populations present in HDX-MS experiments using XylE as the model system. First, we benchmark our approach by calculating the populations present in the wild-type (WT) apo XylE protein ensemble, and in an ensemble driven toward an outward-facing (OF) conformational state by single point mutation (G58W). We find that the predicted conformational populations are relatively invariant to the model used to connect protein structure to residue protection factors and exchange rate. Next, we conformationally describe subtle HDX differences observed between XylE ensembles in different lipid environments. Finally, we characterize the conformational effects of substrate binding to XylE and contrast the calculated substrate-bound ensemble with those of inhibitor-bound states. In doing so, we provide an atomistic description of the mechanism of substrate and inhibitor recognition in XylE and demonstrate an important improvement in making useful structural interpretations with HDX-MS data.

## Results

### Benchmarking

**Figure 1:**
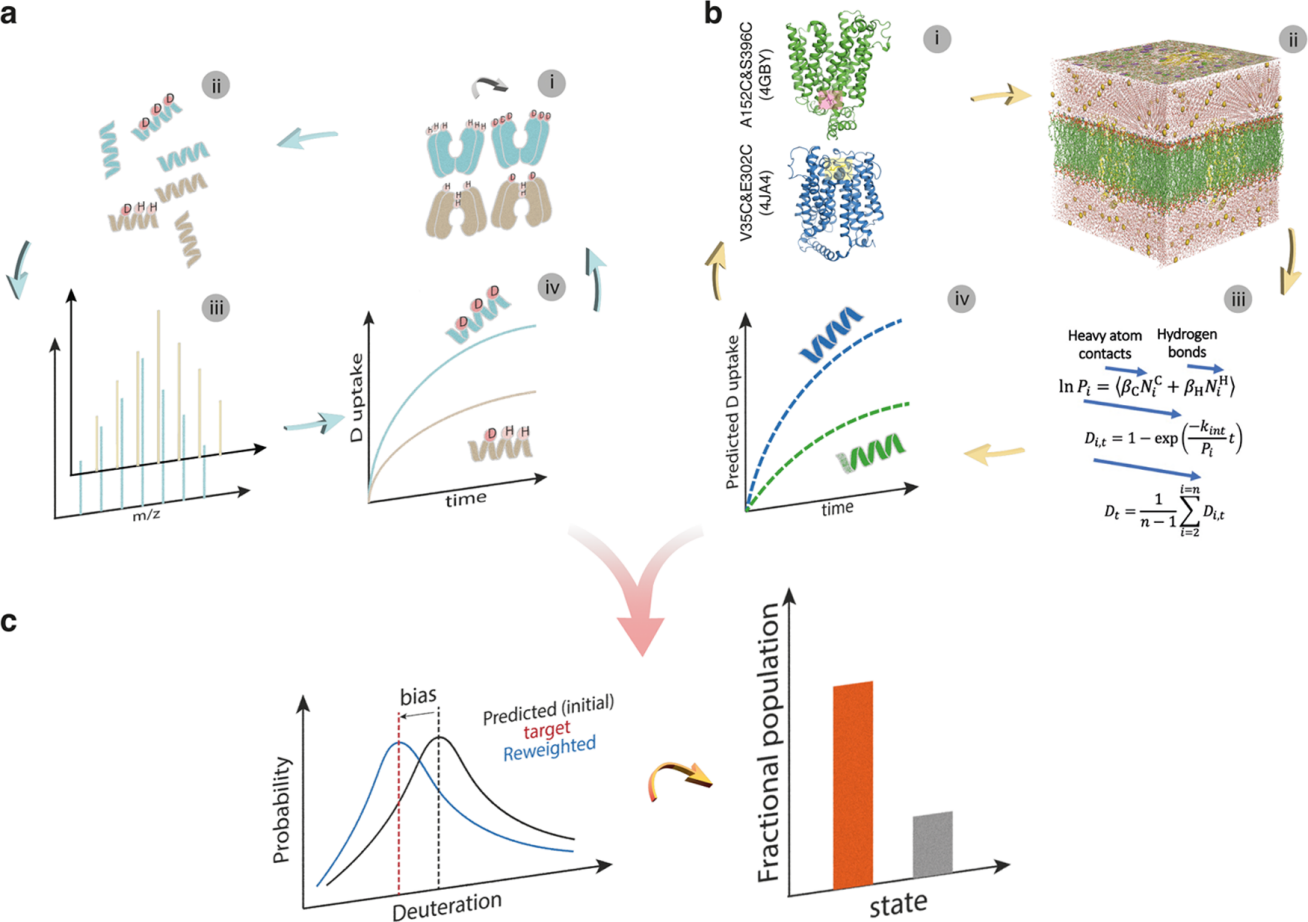
Integrative modeling workflow with HDX-MS data and molecular simulations (a, i) H–D exchange of protein backbone amides occurs spontaneously in deuterated solution. (a, ii) The exchange can be quenched at various time points and measured at peptide-level resolution when coupled to enzymatic digestion. (a, iii and iv) Deuterium uptake over time is determined by measuring the peptide mass by LC-MS. (b, i) Generate initial protein structures. (b, ii) Ensemble sampling of protein structures via MD simulations. (b, iii and iv) Compute deuterium uptake based on an empirical model. (c) Fit predicted deuteration to target data to find an atomistic structural ensemble that best fits target HDX-MS data, then quantify fractional population in the final reweighting ensemble structure.

We developed a three-step approach that brings together experiments and computation. The steps involve: (a) gather and analyze HDX-MS data, (b) generate candidate model structures using MD simulations and subsequently extract simulated HDX-MS data, and (c) reweight the simulated data to fit a model ensemble that best conforms to the target data. Ultimately, the approach allows for quantifying the conformational populations of individual ensemble structures. In the first step, differential HDX-MS is carried out using previously described protocols^21^ (Figure 1a). This enables deuterium uptake measurements to be determined for identified peptides over various time intervals (ranging from seconds to hours). In this study, peptides with significant difference in hydrogen deuterium exchange (ΔHDX) were evaluated using Deuteros 2.0^22^ with a hybrid significance test model.^23^ The deuterium uptake difference is considered as significant if it is greater than a threshold value (99% confidence interval), and the significance is confirmed by a Welch’s *t*-test. As differential HDX-MS data are the results of subtraction of deuterium incorporation between two or more states, experimental uncertainties (e.g., back-exchange level, pH, temperature) are assumed to be equally applied to each state and can be canceled out across the multiple protein measurements. Such datasets are ideal for qualitative analysis, but no computational approaches have been developed to predict or analyze ΔHDX-MS data directly. As a consequence, ΔHDX-MS can currently only describe qualitative structural trends between protein states, instead of precise conformational changes. Here, we normalized each of our measured HDX-MS datasets against a maximally deuterated (MaxD) sample to prepare absolute HDX-MS data, allowing for quantification of HDX-MS measurements across multiple states. In the subsequent step, we deploy μs-long MD simulations^24^ to generate candidate structures of the protein under investigation (Figure 1b). We compute deuterium uptake associated with the generated model structures, using a common empirical model to estimate HDX protection factors (PF) from MD simulations. The model itself (eq 1) is a phenomenological approximation of HDX developed in the early 2000s and commonly used with parameters by Best and Vendruscolo.^25,26^ The model estimates structural protection as an ensemble average function of the hydrogen bonds, *N*_H_, and heavy-atom interatomic contacts, *N*_C_, involving each amide.
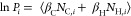
1The parameters β_C_ and β_H_, originally set to 0.35 and 2.0, respectively, arise from an empirical optimization of protection factor predictions with respect to experimental HDX data for several water-soluble proteins.^25^ Given the complex physiochemical determinants of exchange, the optimal values of these parameters can vary depending on the protein or experimental conditions, or indeed correlate poorly with observed exchange.^8,27^ Fortunately, sources of error in our predictions can be interrogated in the final step of the approach.^16^ In particular, here we explore the suitability of our computational model for exchange by testing the effects of reoptimizing the β_C_ and β_H_ parameters, and the effects of systematically including/excluding specific experimental data and molecular simulations.

In the final step, with the experimental and simulated HDX data in hand, we fit simulated deuteration to target experiments. We use the HDXer approach to adjust (“reweight”) the relative populations of models to fit individual absolute HDX-MS data (Figure 1c). This step allows us to find an atomistic structural ensemble that best fits target HDX-MS data and to quantify the fractional population of individual conformational states in the final reweighted ensemble.

**Figure 2:**
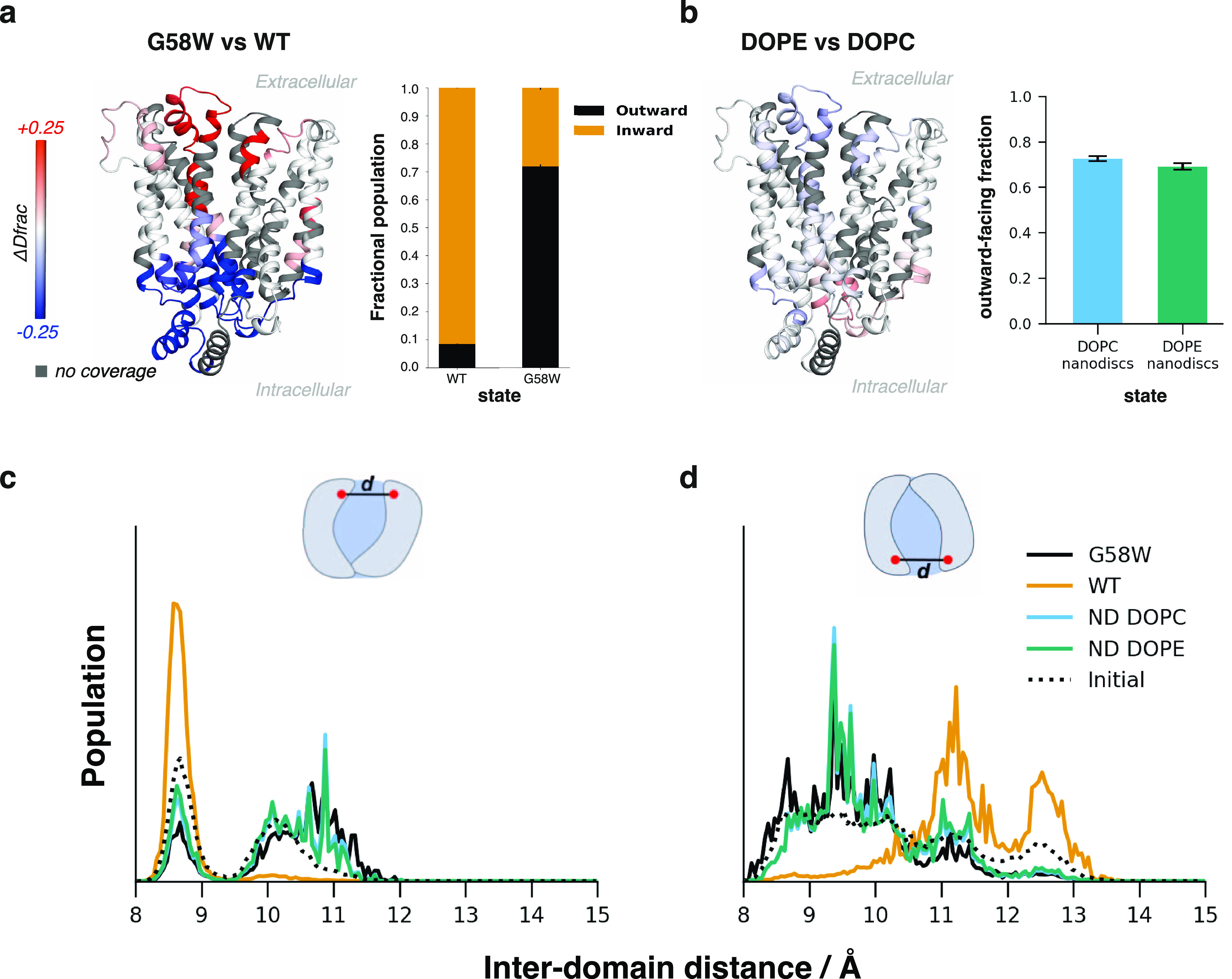
Benchmarking on XylE WT, G58W mutant, and lipid nanodisc. (a). Differential HDX-MS results for XylE WT and G58W solution ensembles highlight significant differences in deuterium uptake between WT & G58W XylE, localized to the extracellular and intracellular protein faces. The experimental data suggest that the G58W mutation shifts the XylE conformational ensemble to a more OF population than WT. Computational reweighting of a mixed OF/IF ensemble to fit the experimental WT and G58W HDX-MS data results in a clear separation of the structures present in each experimental dataset. WT XylE is mostly represented by structures from IF simulations (orange), and G58W by structures from OF simulations (black). (b) Differential HDX-MS results for XylE WT in DOPC-based and DOPE-based nanodiscs show small differences in a structural overlay of DOPC – DOPE ΔHDX-MS. After reweighting, both nanodisc environments consist of a mixed OF and IF ensemble, with populations intermediate between those of WT and G58W XylE in detergent micelles. DOPE-based nanodiscs shift toward a slightly more IF ensemble than DOPC-based. Regions showing a difference in HDX compared to the apo state are colored in blue (protected) or red (deprotected) scale according to the difference in fractional uptake normalized to the MaxD. In both cases, uncertainty in final conformational populations was estimated as the standard deviation from three independent reweighting analyses using systematic subsampling of the full candidate ensemble. Standard deviations of the subsampling for each fitting are plotted as errors (*n* = 3). (c) Effect of reweighting on the XylE interdomain distance distributions on the extracellular and (d) intracellular face of the protein. Larger distances correspond to a more “open” structure.

Initially, we benchmarked our strategy using existing data generated in our group.^2^ In previous studies, we have shown that the equilibrium conformational populations of states in the transport cycle of XylE may be affected by mutations, proton or ligand binding, and detergent or lipid environment.^2,17^ WT apo XylE has been crystallized only in an inward-open state, while observation of an outward-open state required a double mutation (G58W/L315W) of residues lining the extracellular vestibule, sterically hindering the transition to an inward-facing (IF) state. To compare experimental and predicted HDX-MS data, back-exchange-corrected HDX-MS data are required by HDXer. After reanalyzing the original differential HDX-MS data obtained by Martens et al. in order to ensure consistent peptides could be studied across all subsequent states, we performed a maximally deuterated (MaxD) control to obtain the absolute HDX-MS data of WT and the single mutant G58W XylE. In WT XylE, peptides at the intracellular face of the protein exhibit significant deuteration while peptides at the extracellular face are comparatively highly protected. In G58W XylE, the pattern is reversed and peptides at the extracellular face exhibit high deuteration, commensurate with a flexible, solvent-accessible conformational state (Figure S1). Differential HDX-MS analysis highlighted 35 peptides from previous results with significant differences in uptake between WT and G58W. Significant differences were entirely localized to the solvent-facing surfaces of the protein and were qualitatively consistent with those originally analyzed,^2^ suggesting that the G58W protein shifted the relative conformational populations of XylE toward more OF states (Figure 2a). Next, to quantify the magnitude of the shift in conformational populations represented by the previous experimental WT and G58W data,^2^ we performed ensemble reweighting. A mixed candidate ensemble, initially of 50% OF and 50% IF structures (Supporting Methods), was fitted using HDXer to target each of the absolute HDX-MS datasets separately. Targeting the WT HDX-MS data resulted in a final reweighted ensemble of 4.4% OF, and 95.6% IF structures. In contrast, targeting the G58W HDX-MS data resulted in a final ensemble of 80.6% OF and 19.4% IF structures (Figure 2a).

The bias applied to (i.e., the final relative weight of) each structure in the reweighted ensemble is inextricably linked to the model used to predict residue protection factors from the structure. The Best–Vendruscolo model is parameterized to estimate the conformational free energy change of “opening” (Δ*G*_op_) from simulations that predominantly sample the protein exchange-noncompetent or “closed” (C) state. Our simulations are approximately 10^3^-fold longer than the simulations used to parameterize the original Best–Vendruscolo model, and this additional sampling of structural fluctuations may reduce the accuracy of the parameterized model. We therefore investigated the sensitivity of the reweighted conformational populations to changes in the scaling parameters (β_C_ and β_H_) of the Best–Vendruscolo empirical model, by reoptimizing the scaling factors for μs-scale dynamics (Supporting Methods). The alternate optimization resulted in β_*C*_ = 0.29 (original β_C_ = 0.35), β_H_ = 3.9 (original β_H_ = 2.0), and reweighting was again applied to quantify the conformational populations present in the WT and G58W experimental data. The WT ensemble was calculated with final weights of 8.5% OF, and 91.5% IF, while the G58W ensemble was calculated to be 71.9% OF, and 28.1% IF (Figure S2).

Although the absolute magnitudes of the final reweighted populations do change between the two models, the overall structural interpretation that the G58W mutation shifts the conformational equilibrium from predominantly IF to predominantly OF is unchanged. The structural interpretations that reweighting can provide for XylE are therefore relatively robust to small uncertainties in the forward model. Large conformational effects, e.g., associated with point mutations, are therefore clearly amenable to reweighting interpretations, and consistently determined. With that in mind, we pose the question: Are smaller conformational/dynamical differences also interpretable?

Differential HDX signals between DOPC-based and DOPE-based nanodiscs (ND) are largely nonsignificant (Figure 2b) when compared at the individual peptide level. However, the difference in conformational populations observed following HDXer reweighting suggests that the combined HDX measurements may cumulatively reflect a small but consistent structural difference in the global OF/IF populations between the PC- and PE-based lipidic environments.

The conformational shifts associated with each experimental dataset may be explored in greater detail by examining the interdomain distance distributions for each reweighted ensemble. Here, a distinct difference between the WT and G58W or ND distributions is observed (Figure 2c), particularly in the distances observed at the intracellular face of the protein (Figure 2d). After reweighting to the WT data, a substantial population is observed at an intracellular interdomain distance of ∼13 Å, corresponding to a “fully open” conformation, while in reweighting to G58W or ND data, only a “partially open” conformation (interdomain distance ∼11 Å) is observed.

To visualize the structural differences between partially and fully open conformations in more detail, we extracted representative structures (centroids of each sub-distribution) from the intracellular distance distributions after reweighting to either the WT or G58W (Figures S3 and S4) HDX data. The larger interdomain distance observed in the fully open conformation appeared to arise from the motion of the intracellular helical bundle (residues 220–270), which lies away from the intracellular vestibule, packed against the C-terminal intracellular helix. In contrast, in the partially open conformation, the intracellular bundle is swung back toward the vestibule opening, although the intracellular pathway to the binding site remains accessible. Although the fully open conformation is more consistent with an “inward-open” state, we note that the intracellular helices are often only partly resolved in available crystal structures, thus supporting the idea of a high degree of flexibility in this region.

After reweighting to the G58W HDX-MS data, only small populations of IF structures remained. However, the OF conformations of XylE (corresponding to intracellular interdomain distances of 8.0–10.6 Å) also showed substantial flexibility in the intracellular helical bundle. Although these four intracellular helices form a cap to the intracellular vestibule in the outward-open crystal structure, the flexibility of these regions observed in MD simulations appears to be consistent with our HDX-MS data. The final ensembles after reweighting to DOPC or DOPE-based nanodiscs, however, were very similar in terms of OF/IF populations (Figure 2b) and interdomain distances (Figure 2c). The number of intracellular lipid contacts to E153, D337, and E397, which had previously been identified as key residues controlling the inward–outward conformational preference,^2^ also showed no appreciable difference after reweighting to either DOPC or DOPE nanodiscs HDX-MS (Figure S5).

Overall, our analysis implies that the effects of lipid type upon XylE conformational preference are very subtle, although consistent with previous hypotheses. The similarity between the final ensemble populations highlights a limit of the structural fidelity provided by the ensemble reweighting approach—in particular, our target dataset for reweighting does not include peptides covering the E153, D337, or E397 residues, any (or all) of which may be crucial to characterize the subtle effects of lipid composition upon structure.

### HDX-MS Reveals Distinct Protein Dynamics upon Substrate/Inhibitor Binding

Having established the feasibility of our strategy in benchmarking XylE WT and G58W mutant using previously published differential HDX-MS data obtained,^2^ we turned our attention to understanding distinct dynamics of XylE transporter upon substrate and inhibitor binding. We chose to use the substrate xylose and endogenous inhibitor, glucose, as well as exogenous ligands phloretin^28^ and phloridzin,^29^ which are known inhibitors of glucose transmembrane transport.^30^ A previous study has established XylE as a surrogate for the mechanistic understanding of GLUT1 via a conserved mechanism of ligand binding.^20^

**Figure 3:**
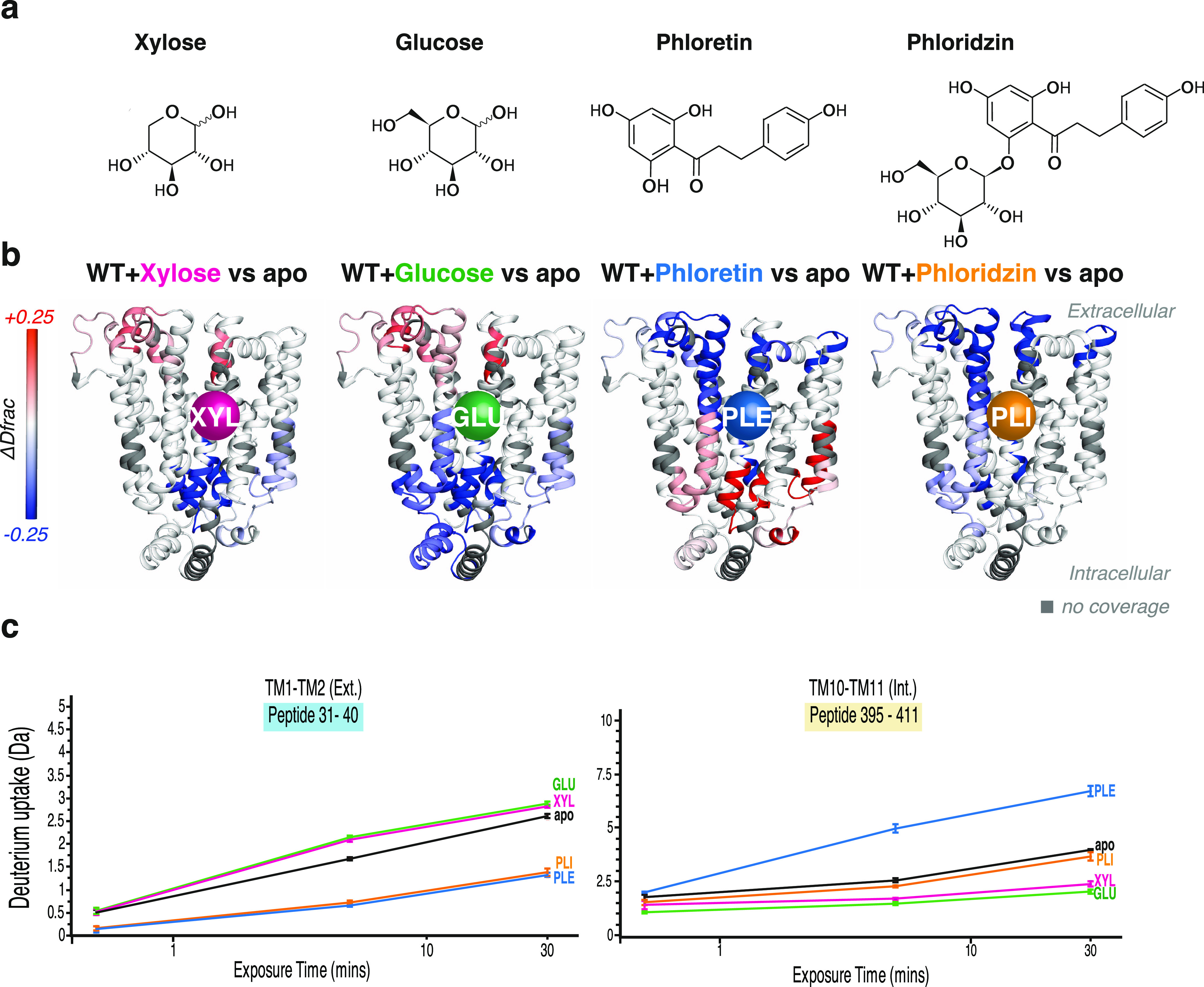
Differential HDX-MS experiments comparing ligand-bound and apo states of WT XylE. (a) Chemical structures of ligands: xylose, glucose, phloretin, and phloridzin. (b) Differential HDX-MS uptake pattern between apo XylE and ligand-bound XylE structures. Figures are plotted onto a 3D protein structure (PDB:4GBY). Regions showing a difference in HDX compared to the apo state are colored in blue (protected) or red (deprotected) scale according to difference in fractional uptake normalized to the MaxD. Regions with no coverage are colored in dark gray. Residue numbers for peptide segments with significant difference between states are reported in Figure S8. (c) Representative peptide deuterium uptake plots between apo and ligand-bound structures (peptide 31–40 on the extracellular side and 395–411 on the intracellular side). Standard deviations for each time point are plotted as error bars (*n* = 3).

To dissect the structural changes made by substrate and inhibitor binding before transport, we performed a new set of differential HDX-MS experiments, together with MaxD experiments, comparing XylE WT apo and in ligand-bound (xylose-, glucose-, phloretin-, and phloridzin-bound) states (Figure 3a). Prior to experiments, the protein and ligands were incubated at a ratio that-according to their binding affinity-enabled to achieve a binding occupancy of approximately 90% after dilution in deuterated buffer. (Table S1). We obtained 82% sequence coverage, allowing us to interrogate the dynamics of XylE in its ligand-bound and ligand-free states along most of the protein sequence (Figures S6 and S7, Table S2).

We began by carrying out differential HDX-MS experiments with the substrate xylose and inhibitor glucose. To obtain comparable conditions with other ligands, which required to be solubilized in DMSO, for each experiment, we equilibrated the protein and ligand together in 10% DMSO solution prior to labeling. This ensured that the presence of DMSO did not adversely affect protein dynamics for any single state, and hence that all states could be fairly compared. We also qualitatively compared the exchange patterns observed in these experiments (Figure S8) to our previously published data,^17^ and we observed analogous conformational fingerprints to the previous results. Specifically, in these new experiments, the presence of xylose and glucose leads to an increase in deuterium uptake on the extracellular side and a decrease on the intracellular side (Figures 3b,c and S8). Such deuterium uptake difference is a typical ΔHDX pattern of a transition toward an OF conformation.

Next, we explored the conformational landscape of WT XylE upon binding to phloretin and phloridzin. Interestingly, we observed a substantially different ΔHDX pattern in the presence of these GLUT inhibitors. Unlike xylose and glucose, phloretin-bound HDX fingerprint shows a decrease of deuterium uptake on the extracellular side (e.g., peptide 31–40) with an increase on the intracellular side (e.g., peptide 396–411), a ΔHDX pattern typical for the transition of transporter towards an IF conformation (Figure 3b,c). Intriguingly, compared to unbound XylE, the presence of phloridzin causes an overall decrease in deuterium uptake on both extracellular (e.g., peptide 31–40) and intracellular (e.g., peptide 396–411) sides (Figure 3b,c). For the phloretin-bound structure, the data suggest that phloretin binding drives the protein toward a more IF ensemble than the apo state. This likely precludes other ligands to bind to the extracellular side of the protein and eventually being transported. In the presence of phloridzin, a decrease in deuterium uptake from both extracellular and intracellular sides suggests an overall protection of the whole protein, consistent with an occluded-like state. It is interesting to speculate that these distinct conformational effects exerted by the two ligands on the structure of XylE may be indicative of different inhibitory pathways, likely reflecting differences in the effectiveness in inhibiting glucose transport, as previously suggested.^31,32^ It has to be noted that phloridzin is a glucoside of phloretin (structure in Figure 3a), suggesting that the conformational effect that this ligand exerts on XylE could arise from a combination of the glucose effect, which stabilizes OF, and phloretin effect, which stabilizes IF. To explore this further and to gain structural insight into the conformational landscape of the inhibitor-bound states, we proceeded to computational analyses using our integrative HDX-MS approach presented above.

### MD Simulations to Predict Protein-Inhibitor Binding Modes

**Figure 4:**
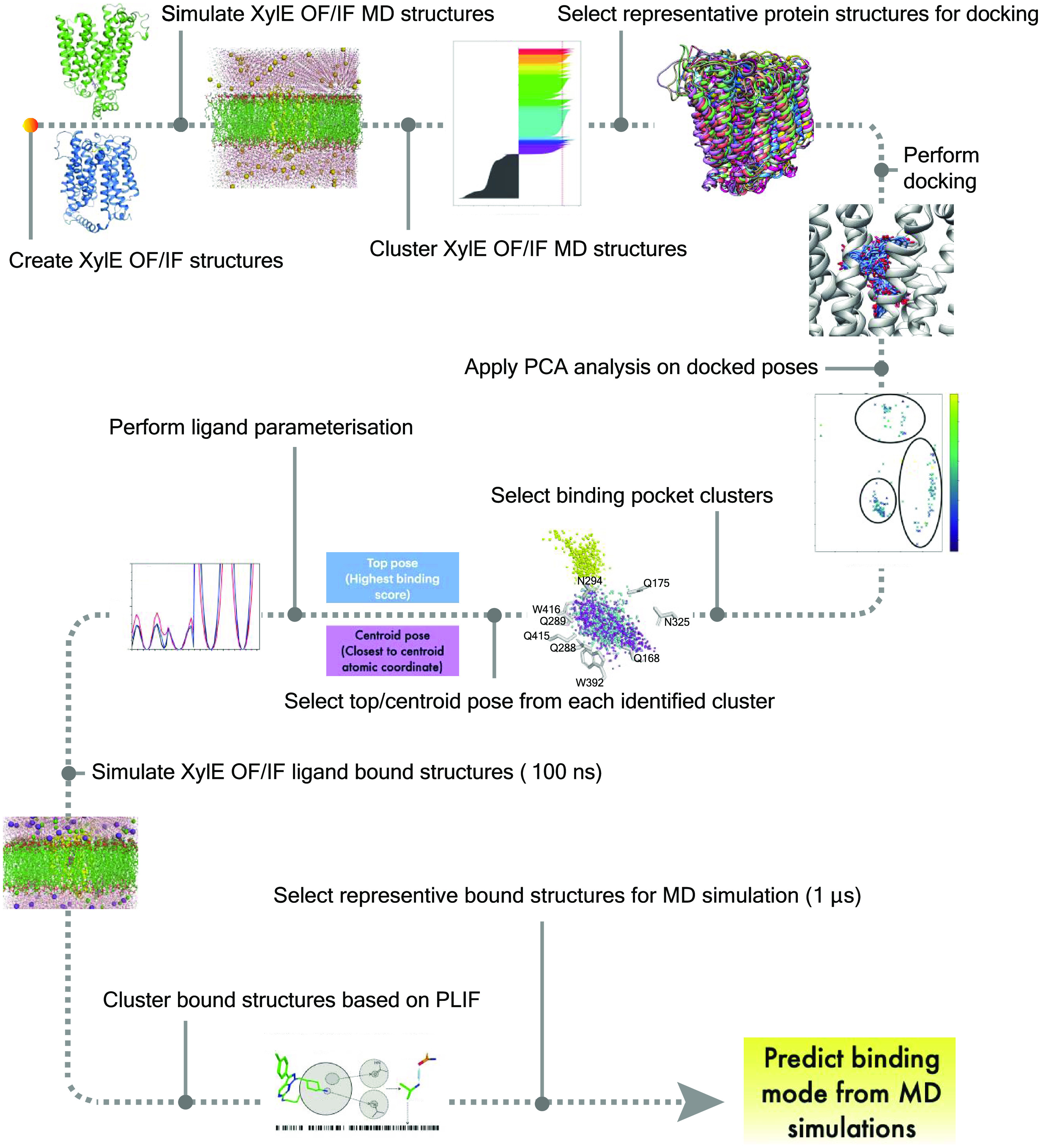
MD workflow of generating XylE phloretin- and phloridzin-bound structures. The workflow comprises generating apo receptor structures by first performing MD simulations of protein-only structure in locked conformation (OF/IF) to generate a broader range of conformations, clustering methods are then applied to select representative protein structure for docking; rigid docking of ligand to representative receptor structures; dimensionality reduction for the identification of binding site clusters and structural clustering for representative (centroid) and highest scoring (top) docking pose selection; simulation of representative/highest scoring ligand-bound structures; and analysis of pose dynamic and selection of suitable binding pose by stability, repeated sampling, and biological relevance criteria.

Integrative ensemble reweighting to each protein–ligand HDX-MS dataset first required a comprehensive candidate ensemble incorporating both OF and IF ligand-bound structures for each ligand. However, crystal structures are only available for OF xylose-bound (4GBY), OF glucose-bound (4GBZ), and IF apo (4JA4) structures. Therefore, to generate ensembles for the remaining protein–ligand states, we first generated conformationally locked protein structures—OF and IF mutants of XylE restricted by cysteine cross-linking mutations (OF: A152C/S396C and IF: V35C/E302C) based on available crystal structures (4GBY and 4JA4) and performed MD simulations (Supporting Methods) to sample the conformationally locked apo-state pocket. Subsequently, we extracted representative pocket (and receptor) structures from each apo simulation using a density-based spatial clustering of applications with noise (DBSCAN) method applied to the dihedral angles for key binding site residues. Docked poses for each ligand were generated using rigid receptor docking in Autodock Vina.^33^ Protein flexibility was instead incorporated by docking to the multiple representative structures from apo-state MD simulations, rather than a single crystal structure. We then subjected the docked poses to principal component analysis (PCA) and clustering in the reduced dimensions to identify highly populated binding modes suitable to initiate MD simulations. Finally, a two-step MD simulation (100 ns and 1 μs) allowed us to validate the stability of the selected bound structures (Figure 4).

**Figure 5:**
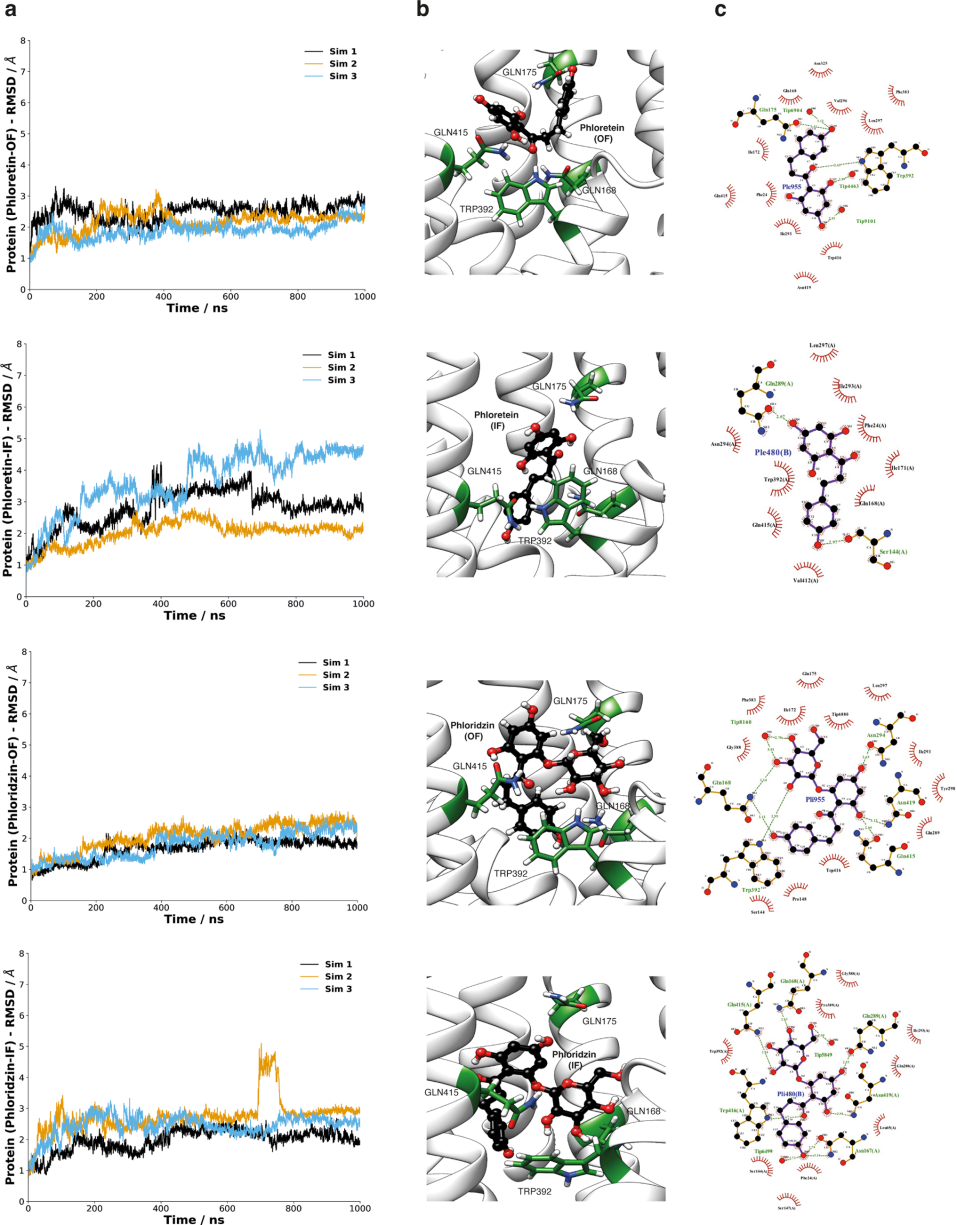
MD simulations of XylE phloretin- and phloridzin-bound structures in the OF and IF conformations. (a) RMSD plots of XylE backbone in bound phloretin and phloridzin (OF and IF) conformations through three independent 1 μs-long simulations. (b) Coordination of phloretin and phloridzin by XylE in OF and IF structures. Phloretin and phloridzin are shown in black balls and sticks, respectively. The binding site residues in XylE are colored green. (c) 2D protein–ligand interaction diagram generated by LigPlot+.^35^ Phloretin and phloridzin are shown in black balls and sticks, respectively, hydrogen bonds are shown as green dotted lines, while the red eyelash diagram represents hydrophobic interactions.

Analyses of the 1 μs-long MD simulations suggest that protein and ligands remain stably bound in OF and IF structures during the vast majority of simulations. (Figures 5a,b and S9). A closer inspection of the data however reveals interesting differences between phloretin and phloridzin. The sugar moiety in phloridzin appears to have a prominent function in binding to XylE. Crystallographic studies have previously shown three glutamines (Gln168, Gln175, and Gln415) in the ligand-binding site are critical in d-glucose recognition by XylE. In particular, Gln168 forms three hydrogen bonds with d-glucose, while it only has one single hydrogen bond with d-xylose. Additionally, Gln175 is hydrogen-bonded with the 6-hydroxyl group of d-glucose but not involved in d-xylose binding.^34^ Therefore, we performed hydrogen bond analysis for glucose-, phloretin-, and phloridzin-bound structures, where the number of hydrogen bonds was calculated over the 1 μs simulation time. Hydrogen-bond interactions between ligand and residue Gln168, Gln175, and Gln415 were analyzed separately (Figures 5c and S10). Our MD simulations show that all three residues form hydrogen-bond interactions with phloridzin in a similar pattern to d-glucose-bound structures in both OF and IF conformations. In contrast, phloretin-bound structures show substantially fewer H-bonds than d-glucose- and phloridzin-bound structures under all analyzed conditions. The results suggest that the glucose moiety in phloridzin plays a pivotal role in binding with XylE. Overall, our computational pose prediction workflow generated stably bound poses consistent with available experimental interaction information and provided new structural insights into the inhibitory function of phloretin and phloridzin upon binding to XylE.

### HDX Reweighting Approaches Quantify Conformational Population

Having gained insights into XylE-inhibitor binding modes by MD simulations, we sought to probe the conformational landscape of XylE in apo and ligand-bound states. Ensemble structures generated by MD simulations were used to predict HDX-MS deuterated fractions for peptide segments corresponding to our experimental HDX-MS data. The calculated deuteration fraction of each time point was compared with the corresponding experimental data. Peptides spanning residue 1–5 or 479 onwards (missing in the crystal structure) or with negative deuteration from experimental data (attributed to experimental noise) were excluded from reweighting analyses.

We performed HDXer analyses of each experimental HDX-MS dataset separately. Initially, for each HDXer analysis, we used a candidate ensemble comprising only the “state-specific” XylE states (i.e., apo simulations fit to apo HDX-MS data, and xylose-bound simulations fit to xylose-bound HDX-MS data, etc.) in a 50:50 mixture of OF/IF conformational populations. HDXer was then applied to each candidate ensemble to obtain a reweighted ensemble with improved correlation to experimental data (Figure S11). We applied a γ value (tightness of fit) corresponding to a reweighting apparent work *W*_app_ of ∼5 kJ/mol to initial ensemble structures to avoid overfitting (Figure S12). As such, the same *W*_app_ was then assigned in each individual reweighting, ensuring equivalent bias was applied to each initial ensemble of structures, and therefore results for each state were comparable.^16^

**Figure 6:**
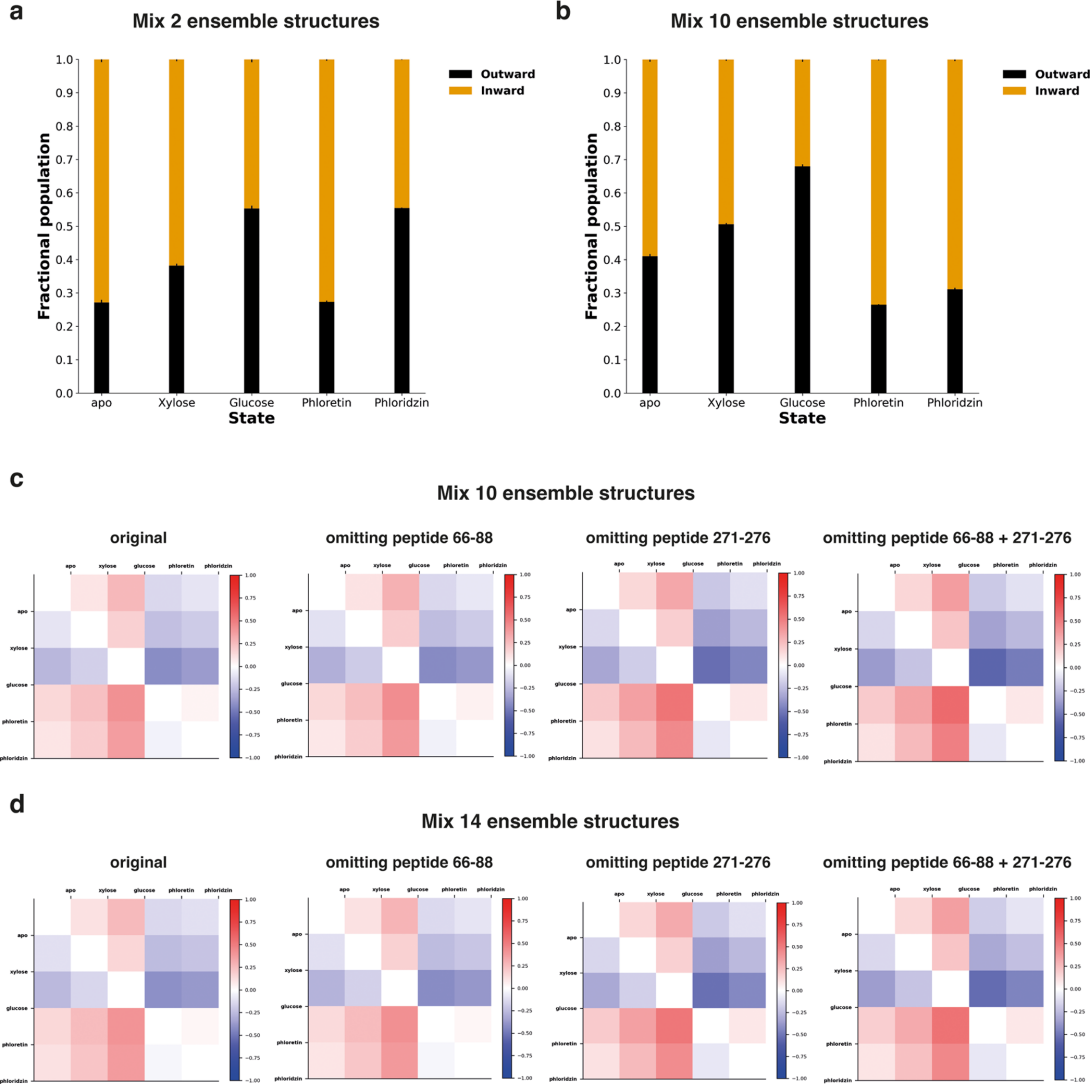
Ensemble reweighting of XylE structures in apo and ligand-bound states. (a) Fractional population of the final reweighting ensemble with a mixed candidate ensemble from two starting structures (OF/IF) fitted to each state-specific experimental HDX-MS dataset (apo, xylose-, glucose- phloretin-, and phloridzin-bound state). (b) Fractional population of the final reweighting ensemble with a mixed candidate ensemble from 10 starting structures (OF/IF) fitted to each experimental HDX-MS dataset (apo, xylose-, glucose-, phloretin-, and phloridzin-bound state). In both cases, uncertainty in final conformational populations was estimated as the standard deviation from three independent reweighting analyses using systematic subsampling of the full candidate ensemble. Standard deviations of the subsampling for each fitting are plotted as errors (*n* = 3). (c, d) Heatmap of relative fraction difference between states. Red indicates more OF, and blue indicates more IF.

Consistent with experimental HDX-MS data, binding of xylose and glucose resulted in an OF-favored conformational equilibrium shift compared to the apo state. Interestingly, the shift is significantly more prominent for the glucose-bound state for which the final reweighting ensemble results in a 55.3% OF population (Figure 6a and Table S3). Strikingly, phloretin-bound structures exhibit a dramatically different conformational landscape to xylose-/glucose-bound structures. The reweighted phloretin-bound ensemble consisted of 72.7% IF structures, consistent with the original visual interpretation of the conformational fingerprint observed in the experimental HDX-MS data (Figure 3b). Interestingly and consistently with HDX-MS experiments, phloridzin-bound structures suggest a different fractional population (55.4% OF) in the final reweighting ensemble compared to phloretin-bound structures. Although this may be reflective of a more occluded-like structure as suggested by the HDX-MS fingerprint (Figure 3b), we do not have fully occluded structures in the simulation ensembles to confirm that.

Next, we mixed all 10 ensemble structures covering five protein states, each with two ensembles in both OF and IF conformations, to perform reweighting with an identical candidate ensemble for each experimental dataset. Noticeably, mixing all 10 ensemble structures to fit experimental data results in a slightly better agreement to target data for all protein states (Table S4), implying some conformations in the “alternate state” structures are better at describing each experiment dataset. However, the overall trend of percentage population for apo, xylose-, glucose-, and phloretin-bound structures remains the same compared to the previous ensemble with only “state-specific” structures. Surprisingly, reweighting of phloridzin-bound structures leads to a different percentage population compared to glucose-bound structures, but largely similar population to that observed in phloretin-bound (73.5% IF) structures, with 68.9% IF conformers in the final reweighting ensemble (Figure 6b and Table S4).

To investigate further the source of the discrepancy, we carried out additional reweighting experiments. Initially, we assessed the impact of experimental noise. RMSE values for each peptide segment were calculated from the ensemble mixture before and after reweighting by HDXer. Peptide 271–276 was identified as the most error-prone peptide across all states (Table S5). Additionally, due to an observed decrease and increase of deuterium uptake in different time points for phloretin-bound structures, peptide 66–88 was also included as an error-prone peptide to investigate how it affects the reweighting results using HDXer (Figure S13). We then carried out reweighting by omitting peptides 66–88 or 271–276 or both from the analysis. Our results suggest that the errors from these peptides are unlikely to impact the observations from the original dataset. Pairwise comparison of relative OF fraction population was plotted for apo and ligand-bound states (Figures S14a and S15). The overall trend of difference in the conformational population remains the same across all tested conditions (original and peptides excluded) (Figures 6c and S14b).

We then set out to assess the effect of sampling errors from insufficient conformational sampling. An additional four atomistic MD simulations initiated from alternate phloretin- and phloridzin-bound poses in both OF and IF were performed (Supporting Methods, Figure S16), adding up to a mixture of 14 ensemble structures for HDXer reweighting. We first checked the fractional populations after reweighting for previously generated ensemble structures, newly generated ensemble structures, or both for phloretin and phloridzin separately (Figure S14d). No distinguishable difference was observed. We then repeated reweighting for the full set of 14 mixed ensemble structures under the same conditions, which resulted in the best agreement with target experiment data obtained so far (Table S6). Interestingly, the mixed ensemble displayed a similar relative percentage population regardless of introducing additional ensemble structures or omitting peptides (Figures 6d and S14c, and Table S6). It is worth noting that our validation was carried out using the same Best and Vendruscolo empirical model,^25^ and any inaccuracies related to the model will systematically be reflected across all of the reweighting processes. Therefore, potential inaccuracies in the forward model should not affect one state more than the other.

## Discussion

In summary, we have presented a detailed workflow for quantifying the conformational landscape of the sugar transporter XylE. Initially, we generated a wide-ranging candidate ensemble of potential XylE structures, encompassing OF and IF transporter states. Subsequently, HDX-MS experiments together with ensemble reweighting suggest that the GLUT inhibitors phloretin and phloridzin exhibit a substantially different mode of action to the endogenous ligands (xylose and glucose). The final reweighted structural ensembles, fitted to each experimental dataset independently, allow us to probe this mechanistic difference at the atomistic level.

Our HDX-MS results indicate that the binding of phloridzin causes overall protection to the protein while its aglycone, phloretin shifts the protein conformational equilibrium toward IF. By combining MD simulations with a post hoc ensemble reweighting approach, we were able to quantify and visualize conformational changes incurred upon inhibitor binding. Overall, our results point to the hypothesis that phloridzin-bound structures cannot be assumed as an ensemble of structures occupying simple OF and IF representations. This hypothesis is supported by the occluded HDX-MS pattern (phloridzin-bound structure vs apo) and mixed (glucose and phloretin) populations after reweighting with only using “state-specific” OF and IF ensemble structures. Our models further revealed the different conformational changes of inhibitor binding and pointed to the key residue contributions for inhibitor binding (Gln168, Gln175, and Gln415). Overall, this interplay between different ligands and proteins offers an entirely new view of the mechanism of action for GLUT inhibitor binding.

As with any integrative modeling approach, the structural hypotheses generated by our HDXer analyses are subject to three main sources of error: in the computational model of exchange, in the simulated candidate ensemble of structures, or in the experimental data acquisition itself. In the absence of corroborating solution-state information from alternate experimental methods (a far from straightforward endeavor for membrane proteins), we instead interrogated our hypotheses with internal robustness checks of variance in the HDXer modeling. Reoptimizing the scaling factors of the empirical HDX model, changing the structures included in the candidate MD ensemble, and excluding specific experimental datapoints from analysis, all imparted quantitative differences to the final conformational populations after reweighting, as would be expected. However, the structural interpretations drawn from the final populations remained consistent throughout, strengthening our hypotheses. Other HDXer studies have also reported similar robustness of their structural interpretations, though it is important to note that the magnitude of potential errors in HDXer modeling may be different across biomolecular systems, and so these internal robustness checks are always a required component of the modeling process.^16^

Finally, by fitting a mixture of XylE WT and G58W ensemble structures to XylE WT experimental data, we observed differences in the final reweighted ensemble between previously published data,^2^ which led to 95.6% IF, and newly generated data, which led to 72.9% IF. It is worth noting that, as previous HDX-MS data were not associated with a MaxD control, we back-exchange-corrected them with a newly acquired MaxD control to enable the data for reweighting. In contrast, new HDX-MS data and associated MaxD were performed at the same time. By comparing these two sets of data, we observed consistently more deuterium uptake in newly generated data than in previously published data; however, the deuterium incorporation level in two MaxD showed only negligible difference (Table S7), indicating that the newly performed MaxD did not perfectly match previously acquired data. This introduced a small but consistent bias in the calculated relative fractional uptake (%) for previously published data and is responsible for the discrepancy in the reweighted ensemble. Despite the experimental discrepancy, we demonstrated the capability of the HDXer approach to reflect conformational effects and capture the differences in the target experimental data.

## Conclusions

In conclusion, we presented an integrated workflow and robust strategy, offering a new way to infer dynamic information from HDX-MS experiments, as well as providing detailed molecular insights into the MFS transporter. The workflow complements and improves existing methods to interpret ensemble-averaged experimental observables by moving from qualitative data interpretation to quantitative interpretation. The methodology not only paves the way for more sensitive and quantitative investigation of structural dynamics for transporters but also could readily be expanded to any proteins of interest, including mammalian transporters (e.g., GLUT1), and potentially benefit subsequent studies.

## Data Availability

Data supporting the findings of this paper are available from the corresponding author upon reasonable request. All of the deuterium uptake plots of the experiments presented for XylE are available on figshare data repository using the following link: (https://figshare.com/s/4f24c0aded2b5f51cd1d). Spectrometry proteomics data have been deposited to the ProteomeXchange Consortium via the PRIDE partner repository with the dataset identifier PXD034387.

## Supporting Information Available

Detailed descriptions of experimental and computational methods, including HDX-MS sequence coverage map; deuterium uptake plots; computational reweighting analysis; molecular docking analysis; MD simulations trajectory analysis; purified XylE sample chromatogram, and summary of HDX-MS measurements and ensemble reweighting results (PDF)
