# Supplementary material for: Integrating Hydrogen Deuterium Exchange–Mass Spectrometry with Molecular Simulations Enables Quantification of the Conformational Populations of the Sugar Transporter XylE
Source: J Am Chem Soc. 2023 Mar 28;145(14):7768–79. doi: 10.1021/jacs.2c06148 (PMC10103171; doi:10.1021/jacs.2c06148)
Supplement: Supplementary file 1 — ja2c06148_si_001.pdf [file ja2c06148_si_001.pdf]

Supplemental Information

Integrating Hydrogen Deuterium Exchange-Mass Spectrometry with Molecular Simulations Enables Quantification of the Conformational Populations of the Sugar Transporter XylE

Ruyu Jia[1†], Richard T. Bradshaw[1 †], Valeria Calvaresi[1], Argyris Politis [1,2,3] *

[1] Department of Chemistry, King's College London, 7 Trinity Street, London SE1 1DB, U.K.

[2] Faculty of Biology, Medicine and Health, School of Biological Sciences, The University of Manchester, Manchester M13 9PT, U.K.

[3] Manchester Institute of Biotechnology, University of Manchester, Princess Street, Manchester, M1 7DN, U.K.

*Correspondence: argyris.politis@manchester.ac.uk

† Authors contributed equally

Table of Contents

***Supporting Methods*..2**

***Supporting Figures*..19**

***Supporting Tables*..50**

***Supporting References*..64**

Supporting Methods

XylE expression and purification

XylE was overexpressed in *E. coli* BL21-AI (DE3) (Invitrogen), transformed with the XylE WT gene and cloned in the (30 µg/ml) kanamycin-resistant pET28-a plasmid (Novagen) modified with a C-terminal 10-histidine tag. Bacteria were grown in 6 baffled flasks each containing 1 L of LB media at 37 °C 220 rpm to an OD_{600} of 0.8. Expression was induced with 1 mM Isopropy-b-D-1-thiogalactopyranoside (IPTG) and 0.1% (w/v) L-arabinose, and growth continued until the value of OD_{600} was flat. The cells were harvested by centrifugation, washed in 200 mL phosphate-buffered saline (PBS) buffer and centrifuged again for 20 min at 4,200 rpm in a Beckman JLA-16.250 rotor. The pellet was then resuspended in 50 mL PBS buffer with 10 mM β-mercaptoethanol and 1 cOmplete protease inhibitor tablet and frozen at -70 °C before purification. Cells were defrosted and incubated with 1.5 µL benzonase nuclease (ThermoFisher) for 10 min at room temperature before passing through a constant cell disrupter at 25 kPsi and 4 °C. Then the ice-chilled membranes were isolated by ultracentrifugation for 30 minutes at 38,000 rpm in a Beckman Ti45 rotor, 4 °C. Membrane pellets were solubilised for 2 hours with mixing in solubilisation buffer [50 mM sodium phosphate pH 7.4, 200 mM NaCl, 10% (v/v) glycerol, 20 mM imidazole, 10 mM β-mercaptoethanol, and 2% n-Dodecyl β-D-maltoside (β-DDM, Anatrace), 0.1 mM phenylmethylsulfonyl fluoride (PMSF) and EDTA free protease inhibitor tablet (Roche)] at 4 °C. The protein solution was then isolated by centrifugation for 30 min at 38,000 rpm in a Beckman Ti70 rotor to remove insoluble material. The supernatant was filtered using 0.45 µm filter and applied to a Ni-NTA column equilibrated in 96% SEC purification buffer [50 mM sodium phosphate pH 7.4, 10% (v/v) glycerol, 2 mM β-mercaptoethanol, and 0.05% β-DDM (Anatrace), 0.1 mM phenylmethylsulfonyl fluoride] and 4% elution buffer [50 mM sodium phosphate pH 7.4, 500 mM imidazole, 10% (v/v) glycerol, 10 mM β-mercaptoethanol, 0.1 mM phenylmethylsulfonyl fluoride (PMSF) and 0.05% β-DDM (Anatrace)]. The bound protein was washed with 50 mL 85% SEC purification buffer – 15% elution

buffer and eluted with 2 mL of 100% elution buffer. The eluate was collected for further size exclusion chromatography (SEC). The SEC purification was conducted with a Superdex 16/600 GL SEC column, equilibrated with SEC purification buffer. The elution fraction containing XylE was collected and concentrated with a Vivaspin concentrator (100 kDa cutoff) (**Figure S17**). The samples were either flash frozen and kept at -70 °C until use or used directly for HDX-MS experiments.

Ligand solution preparation

D-xylose was purchased from Santa Cruz Biotechnology. D-glucose (>99.5%) was bought from SIGMA life science. Phloretin and phloridzin were purchased from Merck life science. All ligands were first dissolved in 100% DMSO as phloretin and phloridzin were insoluble in pure water. Subsequently, the solubility of ligands in 10% and 1% DMSO was tested separately to ensure the solubility in equilibration and deuterium labelling conditions (10 times dilution).

Peptide identification

Peptide identification of XylE was performed by liquid chromatography-tandem mass spectrometry (LC-MS/MS) analysis using a Synapt G2-Si HDMS coupled to nanoACQUITY UPLC (Waters). XylE in detergent micelles were prepared at a concentration of around 15 μM using 100 kDa cutoff Vivaspin concentrators. The protein sample (2.25 μL) was incubated with 0.25 μL DMSO and 22.5 μL equilibration buffer E (10 mM potassium phosphate in H_2O pH 7.0) and added with 25 μL ice-cold buffer Q (100mM potassium phosphate in formic acid pH 2.5), simulating labelling conditions. Then, protein samples were injected into the LC system and digested online with a self-packed pepsin column at 20 °C. Peptides were trapped for 3 min using an Acquity BEH C18 1.7 μm VANGUARD pre-column at a 200 μL/min flow rate in solvent A (0.1% formic acid in HPLC water, pH 2.5) before eluted to an Acquity UPLC BEH C18 1.7 μm analytical column with a linear gradient (8-40%) of solvent B (0.1% formic acid in acetonitrile) at a flow rate of 40 μL/min. All trapping and

chromatography were kept at 0 ºC. Then, peptides went through electrospray ionization in positive ion mode and were analysed using a Synapt G2-Si mass spectrometer (Waters) within the mass range 100-2000 m/z. Leucine Enkephalin was applied for mass accuracy correction and sodium iodide was used as calibration for the mass spectrometer. MS^E data were collected by fragmenting with a 20-30 V trap collision energy ramp. Five protein injections were performed for peptide identification. To minimize peptide carryover, the pepsin column was washed once between injections using a pepsin wash solution (1.5 M Gu-HCl, 4% (v/v) MeOH, 0.8% (v/v) formic acid) and an LC run with a sawtooth gradient was conducted between each sample injection to wash the analytical segment.

Continuous deuterium labelling of XylE

XylE in detergent micelles were prepared at a concentration of around 15 μM using a 100 kDa cutoff Vivaspin concentrator. Protein and ligands (xylose, glucose, phloretin and phloridzin solubilised in DMSO) were incubated at ratio enabling about 90% binding occupancy after dilution in deuterated buffer (**Table S1**) according to **Equation 2**, for an estimation of the fraction of the protein in bound forms (f_B) from the starting concentration of protein ($[P_0]$) and ligand ($[L_0]$) with binding affinity (K_d). To enable HDX, the protein alone and the protein incubated with ligands were 10-fold diluted with the deuterium labelling buffer L (10 mM potassium phosphate buffer in D_2O, pD 7.0) to initiate the exchange reaction at 20 ºC. An aliquot of 25 μL (containing 2.5 μL of protein) was withdrawn from the labelling mixture at various time points (30 s, 5 min, and 30 min) and quenched 1:1 with 25 μL ice-cold buffer Q. After quenching, samples were left on ice for 10 s before flash freezing them in liquid nitrogen and kept at -70 ºC until LC-MS analysis. Technical triplicates were performed at every time point and condition studied.

Equation 2

$$f_B = \frac{[P_0] + [L_0] + K_d - \sqrt{([P_0] + [L_0] + K_d)^2 - 4[P_0][L_0]}}{2[P_0]}$$

LC-MS analysis of HDX samples

Frozen quenched samples were rapidly thawed and injected onto a Waters nanoACQUITY UPLC system with all trapping and chromatography elements set at 0 ºC. Then, the protein was digested with a self-packed pepsin column with pepsin immobilized on agarose resin beads at 20 ºC. The pepsin column was washed 4 times between injections using the pepsin wash solution. Two sawtooth runs were done between each sample run to reduce peptide carry-over. Peptides were trapped for 3 min using an Acquity BEH C18 1.7 μm VANGUARD pre-column at a 200 μL/min flow rate in solvent A before eluted to an Acquity UPLC BEH C18 1.7 μm analytical column with a linear gradient (8-40%) of solvent B at a flow rate of 40 μL/min. Then peptides went through electrospray ionization progress in a positive ion mode using Synapt G2-Si mass spectrometer (Waters). The mass range for MS was m/z 100 to 2000 in positive ion mode. Leucine Enkephalin was applied for mass accuracy correction and sodium iodide was used as calibration for the mass spectrometer. MS data were collected by a 20-30 V trap collision energy ramp. All the isotope labelling time points were performed in triplicate.

Maximally deuterated control

XylE (in DDM micelles) was concentrated to 20 μM in 50 mM sodium phosphate, 100 mM NaCl, 10% (v/v) glycerol, 1 mM β-mercaptoethanol, 0.02% β-DDM pH 7.4. 2 μL of protein sample were diluted in solvent A and repeatedly injected into the LC system. Following online pepsin digestion,

peptides eluting from the pepsin column were collected. Three injections were repeated, the eluted fractions mixed together and then split again into three samples. Peptides were lyophilized on a freeze dryer. Each aliquot of lyophilized peptides was incubated with 2.5 μL of buffer E and diluted 10-fold with 6 M deuterated urea in D_2O at 20 ºC, resulting in identical deuterium fraction as for labelled proteins. The reaction was allowed to proceed for 3 hr, before quenching with 25 μL buffer Q. Quenched samples were kept on ice for 10 s, then flash-frozen and stored at -70 ºC until LC-MS analysis. Analysis of the maximally deuterated samples was carried with the pepsin column replaced with a metal union and LC-MS analysis was conducted as for standard deuterated samples.

HDX data evaluation and statistical analysis

Peptide identification was performed by processing the acquired MS^E data with PLGS (ProteinLynx Global Server 2.5.1, Waters). DynamX v.3.0 (Waters) was used to further filter peptides with 0.25 fragments per amino acid and identified in at least 4 out of 5 acquired MS/MS files. To obtain a final peptide map, spectra were further visually inspected in DynamX to exclude peptides of insufficient quality or misidentified. Then all the MS data including undeuterated references and deuterated samples were processed by DynamX for calculation of deuterium incorporation. Peptides with a statistically significant difference in HDX were determined by in-house Deuteros 2.0 software[1] with a hybrid significance test with a 99% confidence interval.

Re-parameterization of empirical HDX-MS model scaling factors

We re-parameterized the scaling factors β_C and β_H in our empirical HDX model to evaluate the sensitivity of the reweighted conformational populations to changes in the model. Identically to the original approach used by Best and Vendruscolo, we re-fitted the scaling factors β_C and β_H to experimentally-measured HDX protection factor data for seven globular proteins: barnase[2], horse heart cytochrome C[3], staphylococcal nuclease[4], ribonuclease H[5], equine lysozyme[6], human α-

lactalbumin[7], and bovine pancreatic trypsin inhibitor[8]. Each protein was initially prepared from an available PDB structure (1A2P, 6K9J, 1EY0, 2RN2, 2EQL, 3B0O, and 5PTI) using the Amber ff14SB force field[9], solvated in a box of TIP3P solvent[10] and 150 mM NaCl such that no solute atom was < 10 Å from the edge of the box, and charge neutralised by the addition of Na^+ or Cl^- ions as appropriate. Each system was then minimised using 5000 steps of steepest descent minimisation, equilibrated to 300 K and 1 bar pressure over a 2-step equilibration protocol totalling 100 ps, and then further simulated for a total of 500 ns. Simulated contacts and hydrogen bonds were then calculated for each protein system, j, and each protein residue, i, and optimal β_C and β_H scaling factors determined by minimizing the mean square difference between experimental protection factors and simulated protection factors, $\rho = \sum_{i,j}\left(\overline{\ln P_{i,j,\mathrm{sim}}} - \ln P_{i,j,\mathrm{exp}}\right)^2$. All simulations were performed in Gromacs (2020.1) on the research computing facility at King's College London, Rosalind (https://rosalind.kcl.ac.uk/).

Preparation of XylE structures for MD simulations

Initial protein structures were downloaded from the Protein Data Bank (PDB) (https://www.rcsb.org/) with PDB ID 4GBY for outward-open partially occluded conformation and 4JA4 for IF conformation. Missing residues in the IF XylE structure (built from 4JA4 chain A) were rebuilt using Modeller 9.24[11]. The 4JA4 sequence with gaps was first aligned to that of 4GBY so that the same residues (5-479) were present in both final structures. 10 initial models of the missing loop sequences were generated, using 4GBY as a template for the loop structures and 4JA4 as a template for the remaining parts of the protein. For each initial model, residues in the loops were remodelled using the loop model functionality, to give 5 alternate loop structures. From the initial model with the lowest molpdf score and DOPE score, the loop conformation with the lowest subsequent molpdf score and the DOPE score was chosen as the initial IF structure for MD simulations. Restrictions using cysteine cross-linking (A152C/S396C or V35C/E302C) were applied to the protein in separate simulations. Site-

directed mutations were introduced into structures using PyMol with structural-guided identification of loci for cysteine mutations. An intracellular cysteine pair A152C and S396C was introduced in the outward-open partially occluded XylE structure (PDB 4GBY), and an extracellular cysteine pair (V35C and E302C) in the inward-open XylE structure (PDB 4JA4), with a sulfur-sulfur distance of 2.1 Å in both structures. These restrictions have been previously validated experimentally to lock the protein in OF or IF states[12]. Possible Asn/Gln/His sidechain flips were checked by uploading to MolProbity[13], followed by manual visual inspection on PyMol where sidechain flips were determined in **Table S8**.

Setup of XylE cross-linked states in POPE bilayer system

The construction of XylE structures with cross-linked mutations in the membrane system was prepared by CHARMM-GUI[14, 15]. Terminal group patches were set to ACE and CT3 for the N-terminus and C-terminus, respectively. POPE lipids were selected at a 1:1 ratio for bilayer construction, the final upper leaflet number is 177 and the lower leaflet is 178. The protein was aligned along the z-axis and the bilayer in the xy-plane. Structures were oriented in the membrane by the PPM web server[16]. The system was then solvated with 25124 TIP3P water molecules, resulting in a cuboid periodic box of 110 Å x 110 Å x 112 Å. Na^+ and Cl^- ions were added to neutralise the system charge and to create an ionic atmosphere of 150 mM NaCl. Protonation states of the titratable residues were assigned by the H++ server[17] (http://biophysics.cs.vt.edu/). Specifically, E206 was protonated, consistent with the previous study, and all remaining residues were assigned to their standard protonation states at pH 7.0.

MD simulations of XylE cross-linked systems

Protein simulations were performed in Gromacs (2020.1) on the research computing facility at King's College London, Rosalind (https://rosalind.kcl.ac.uk/), Jade (https://www.jade.ac.uk/) and HPC

cluster Gravity (https://apps.nms.kcl.ac.uk/wiki/gravity/). The whole system was first energy-minimized for 5000 steps without any positional or dihedral restraint using a steepest-descent method to relax any steric clashes. The system was then equilibrated to 303.15 K and 1 bar pressure over a 6-stage equilibration protocol with a total simulation time of 100 ns. Positional restraints on the protein and lipids and dihedral restraints on lipid head groups were added to maintain geometry and chirality as the box size was equilibrated. The force constants of positional or dihedral restraints are specified in **Table S9**. Restraints were gradually decreased across the first 5 stages, allowing the protein sidechains to move first, then reducing the force constant on the backbone restraints, and finally only the protein Cα atoms were restrained in step 5 (**Table S9**). In the final stage – before production runs, an unbiased 60 ns NPT equilibration was carried out to prepare the system for production MD (**Figure S18**). A 100 ns production run was then performed at constant temperature (303.15 K) and constant pressure (1 bar) using a Nose-Hoover thermostat and Parrinello-Rahman barastat[18] in a timestep of 2 fs. After completion of production runs, trajectories were read in MDTraj (https://www.mdtraj.org/1.9.5/index.html) and imported into Visual Molecular Dynamics (VMD v1.9.4) (https://www.ks.uiuc.edu/Research/vmd/) for visual inspection.

Extraction of representative MD structures

Representative protein structures from apo-state MD simulations were extracted using the DBSCAN (Density-based spatial clustering of application with noise) clustering method based on the chi1 dihedral value of 10 binding site residues (Phe24, Asn294, Asn325, Gln168, Gln175, Gln288, Gln289, Gln415, Trp392, Trp416). Three replicates of the simulations for OF/IF structures were concatenated into one trajectory with 12003 frames in total. Dihedral angles of the aforementioned residues were firstly calculated using Gromacs tools (gmx angle). Each dihedral angle was then represented by the sine and cosine of the angle, which turns the angles into a point on the unit circle. The distances between points (represented as the chord length) were clustered with minPts set as 60 and epsilon

ranging from 0.50 to 1.35 at 0.05 intervals. Each epsilon was assessed with a Silhouette score to choose an optimal cluster size and number of clusters. Epsilon values of 0.55 for both OF/IF structures were chosen. The mean value of the distance between points in each cluster was calculated, and the frame closest to this mean value was then extracted as the representative frame. In total, for OF conformation, 19 representative MD structures were chosen, whereas 11 were selected for IF conformation.

XylE-ligand binding mode prediction

Autodock Vina was used to predict the mode of binding of the ligands discussed in this study. Before docking, the structures of the GLUT inhibitors (phloretin and phloridzin) were downloaded from PubChem[19]. Xylose and glucose were extracted from corresponding crystal structures from Protein Data Bank (4GBY; 4GBZ). Both the representative protein frames extracted from MD simulation and original XylE crystal structures (4GBY; 4GBZ; 4JA4) were used as receptors. Receptor structures were prepared using Autodock Tools. The initial ligand and protein structure file (pdb) including the desired positioning of hydrogens was created with Autodock Tools. For the ligand structure, a torsion tree (a list of the rotatable torsions ready for sampling) was created for a selection of rotatable bonds. The completed protonated and torsion-parameterised ligand and protonated protein (receptor) structures were saved as a pdbqt file. A grid box used to define the centre and size of each docking run was defined as a dimension of 22.5 x 22.5 x 30 Å with a box centre sitting at the CG atom of residue Trp392. For each receptor structure, a maximum of 9 possible poses of each ligand were generated.

Dock xylose/glucose to crystal structures

To test the capability of Autodock Vina, it was applied to dock xylose/glucose back to crystal protein structures (4GBY; 4GBZ). Xylose/glucose docking was tested with two levels of 'exhaustiveness'

(conformational sampling) specified in the configuration file, with the exhaustiveness value set at 8 and 96. Docked poses for xylose/glucose into crystal structure (4GBY/4GBZ) were converted from .pdbqt to .pdb file using Open Babel[20] for further analysis. Each docked pose generated from several distinct representative protein structures was first aligned to the crystal structures 4GBY and 4JA4 respectively using a custom VMD script. Given that the ligand atom order from original crystal structures is different from the docked poses, the atom order of xylose and glucose in crystal structure was edited to match exactly with the docked poses achieved from Autodock Vina using a custom python script. Both xylose and glucose were successfully docked into the ligand-binding pocket of XylE since the top-scoring docking pose showed an almost identical binding pattern to the crystal structure (**Figure S19**).

Dock xylose/glucose to representative MD structures

Rigid docking (receptor structure treated as rigid-body) was carried out for xylose/glucose into representative protein structures (OF: 19; IF: 11) from MD simulations. For each ligand, up to 171 (OF) and 108 (IF), possible protein-ligand structures were generated, covering a range of different binding poses. The RMSD of the conformation between docked poses of xylose/glucose and their poses inside crystal structure was calculated using a custom RMSD calculation python script, each pose was assigned with an RMSD value. The distribution of the RMSD values between docked poses and ligand from crystal structure was plotted using a custom python script (**Figure S20a**). Multiple docking poses with high variance were then clustered using DBSCAN and hierarchical clustering methods. The top pose from the binding pocket cluster using DBSCAN and hierarchical clustering methods is identical. RMSD value between the top pose and crystal ligand structure was calculated as 1.626 Å (**Figure S20b**).

Dock phloretin/phloridzin to representative MD structures

Two GLUT inhibitors (phloretin and phloridzin) were docked into crosslinked XylE OF and IF structures extracted from MD simulations using the same protocol for xylose/glucose. Docked poses for each inhibitor were clustered using Principal Component Analysis (PCA) to the ligand heavy atom coordinates (after alignment of all structures to a reference binding site conformation), to reduce the dimensionality of the clustering problem. Poses were projected onto PC1 & PC2 and then clustered simply into quadrants using visually chosen PC-value cutoffs (**Figure S21**). In this way we first identified clusters of ligand binding poses, from which a centroid and a top-scoring ligand-bound structures were selected as the cluster representatives for further pose refinement by MD simulation (100 ns).

Parameterization of ligand molecules

Force field parameters for small molecules are not covered by the CHARMM36m bimolecular force field. The CHARMM General Force Field program (CGenFF)[21-23] was used to perform atom typing and assignment of parameters for phloretin and phloridzin structures. Quantum-Mechanical (QM) calculations were carried out using Gaussian 09[24] to validate and optimize parameters that exhibited high 'penalty scores' (>10) from CgenFF. A detailed parametrization protocol was followed using a plugin for the VMD software package known as Force Field Toolkit (ffTK)[25] to make sure the parameters were fitted to data from high-level QM calculations. Conformations of the ligand molecules with minimum energy were obtained from PubChem. An initial set of parameters was assigned with the CGenFF server, creating a parameter file (.str) containing the assigned parameters beyond those in the normal CGenFF parameter file (.prm). A Gaussian input file for geometry optimisation at MP2/6-31g* level was created using the "Opt. geometry" tab in ffTK, and the initial molecular conformation was then optimised and saved as a QM geometry optimised conformation (pdb file) for subsequent charge and dihedral optimisation. QM optimization of the charge was carried out on Gaussian 09 where input files were generated via ffTK using the "Water Int" tab, in which

water molecules were oriented to interact with functional groups in the molecule[22]. The orientation of each interacting water molecule depends upon whether the nearby atom is an H-bond donor or an H-bond acceptor based on their chemical environment. H-bonding interactions are predominantly based on electrostatics in molecular mechanical force fields. Positively charged atoms prefer to interact with the water oxygen atoms as H-bond donors while negatively charged atoms will interact with the water hydrogen atoms as H-bond acceptors, and carbons are assigned as both H-bond donors and H-bond acceptors in two Gaussian input files. Gaussian calculations were performed at the HF/6-31g* level for water interactions. Long-distance (> 4 Å) interaction data was removed from the fit of QM calculation to MM data in the charge optimisation process, as water molecules occasionally 'fly off' during optimisation due to steric clashes or unfavourable interactions arising from the automated water insertions. The target Gaussian water interaction data was read in ffTK via "Opt.Charges" tab, the Tolerance is set as default (0.005) which determines the convergence criterion of the fitting process. The Distance and Dipole weights, determining the relative importance of matching MM Distance and Dipole to QM data, were set at a relative weight of 2:1:1 Energy:Distance: Dipole in which the Distance and Dipole weighting were each set to 0.5. After the first iteration of optimisation, the charges were re-optimised at a smaller value of Tolerance (e.g., 0.001), the iterations were repeated until the final charges no longer changed from one step to next and then saved as an updated parameter file with the new charges. Like charge optimisation, dihedral optimisation requires computing the target data from high-level QM calculations first, then fitting MM dihedral parameters to target QM data. Initial dihedrals within the molecule of interest can be identified automatically in ffTK. In the Gaussian files, a torsional scan was performed by rotating each dihedral angle in steps until it rotates 180 degrees; the remainder of the molecule is allowed to relax at each step, to isolate the contribution of the energy associated with the dihedral of interest. Parameters with penalty scores under 10 from the initial CGenFF parameter assignment were removed from dihedral optimisation for fitting each dihedral at MM level to the QM target energy profile. After the first iteration of

optimisation with tolerance set as 0.01 and energy cut-off at 10.0 kcal/mol under simulated annealing optimisation mode, further refinement of the dihedral parameters was performed until the fitting converge using energy profile visualization in ffTK[26], and optimized dihedral parameters were updated in the final ligand parameter file for MD simulation runs.

Protein-ligand complex simulation setup

The selected phloretin/phloridzin structures from docking and the associated representative apo-state MD protein structures were first merged together using a PyMol script. The membrane system was built using CHARMM-GUI membrane bilayer builder. Both protein and ligand were selected and uploaded into the CHARMM-GUI input generator. To create a CHARMM-GUI recognizable ligand parameter file, an initial rtf file was generated using the CHARMM-GUI ligand reader & modeler with the same mol2 file used for CGenFF. The charges in the topology (.rtf) file were manually updated with the optimised charged values from ffTK ligand parameterisation, and the ligand atom names and orders in the merged pdb file were manually updated to match those in the mol2 file. The final parameter file (.par) with optimised dihedral angles is recognizable to CHARMM-GUI, which is therefore directly uploaded using the “upload CHARMM top & par hetero chain” option together with updated topology (.rtf) file for the ligand. The POPE bilayer construction and system preparation for MD simulations was done in CHARMM-GUI as described in the previous section (preparation of XylE in the POPE system).

Short MD simulations (100ns) of selected XylE-inhibitor bound structures

MD simulation protocol was similar to previously described for apo-state protein structures, except for some minor changes in restraints which are summarised in **Table S10**.

Protein ligand interaction fingerprints

Protein ligand interaction fingerprints (PLIFs) were used to fully characterize the possible protein-inhibitor binding modes from MD simulations. Calculation of PLIFs was performed using the python package Open Drug Discovery Toolkit (ODDT)[27] across the trajectories. Pairwise Tanimoto coefficient (**Equation 3**) was then calculated based on interaction fingerprints, where $|A \cap B|$ represents the number of the common ON bits presented in both string A and B, and $|A \cup B|$ is the union set, where the number of ON bits present in either string A or B is counted.

Equation 3

$$T_c(A, B) = \frac{|A \cap B|}{|A \cup B|}$$

The distribution of the similarity score was first visualized in a heat map to identify converged clusters using a custom python script (**Figure S22**). Due to high variance, the hierarchical clustering method was then applied to identify clusters to select the most representative XylE-inhibitor bound structures from each short MD simulation of possible binding mode. Identified clusters were chosen for further analysis where all the following 3 requirements were met: (1). The size of the cluster was at least 10% of all MD frames. (2). The cluster contained at least one frame from trajectories initiated from each possible binding pose. (3). The majority of ligands inside the cluster were within 1.5 Å RMSD to the average structure (**Table S11**). The mean value of the distance between ligand conformation in each identified cluster was calculated, and the frame closest to this mean value was then extracted as the representative frame. Representative phloretin- and phloridzin-bound structures from identified clusters were extracted directly from previous short MD simulations (100 ns). An overall MD simulation workflow for equilibrium simulations is summarized in **Figure S23**.

Long MD simulation of XylE-ligand bound structures (1 μs)

Long MD simulations of phloretin- and phloridzin-bound structures were performed using the representative structure selected from the 100 ns simulation directly following the same MD simulation protocol described in the previous section (100 ns simulation protocol), only extending the production stage for 1 µs (**Table S10**). To compare phloretin- and phloridzin-bound structures with xylose- and glucose-bound structures, additional 1 µs MD simulations were performed. For xylose- and glucose-bound XylE OF structures, pdb structures (4GBY and 4GBZ) were used directly (substrate/inhibitor-bound protein structure only) following the simulation set-up preparation described in the previous section (Setup of XylE structures). Xylose- and glucose-bound IF structures were generated using Autodock Vina by docking xylose and glucose separately into IF apo-state crystal structure (4JA4). MD simulation protocols used were the same as phloretin- and phloridzin-bound structures.

Analysis of 1 µs XylE-ligand bound simulations

Trajectory files were read using Gromacs tools, and 10 separate input trajectory files, each accounting for 100 ns, were concatenated with the gmx trjcat tool in sorted order while frames with identical time stamps were removed. The concatenated trajectory was further modified with gmx trjconv, where atoms were centred in the box and frames were saved at a time step of 250 ps. The RMSD of ligand structure was computed using gmx rms for each 1 µs trajectory with 4001 frames in total. Each frame from the trajectory was superposed to the first frame with structures fitted onto protein transmembrane regions. A RMSD calculation followed, based on ligand heavy atoms. Output RMSD calculation results were read in a python script and plotted for visual inspection. Xylose in IF structure (4JA4) dissociated over the 1 µs simulation time, therefore XylE xylose-bound IF simulation was treated as XylE apo IF ensemble structures for further analysis. The number of hydrogen bond contacts between ligand (glucose, phloretin and phloridzin) and residues (Gln168, Gln175, and

Gln415) were calculated using gmx hbond. Output from contact calculations (number of hydrogen bonds as a function of time) was read in a custom python script and plotted for visualization.

MD simulations of XylE WT apo and G58W (1 μs)

Simulations of WT apo XylE were initiated from PDB entry 4JA4. Simulations of G58W XylE were initiated from 6N3I with the L315W mutation reverted to the wild-type leucine sequence. Following initial structure preparation, systems were protonated, minimised, and equilibrated following an identical 6-step protocol to the inhibitor-bound structures, followed by a 1 μs production simulation. Analysis of simulations was carried out as described in the aforementioned section.

MD simulations data preparation to run HDXer

An atomistic ensemble of protein structures from MD trajectories was generated using gmx trjconv, with the system centred in the box and the centre of mass of molecules put in the box. For each 1 μs simulation, 4001 frames were saved at the interval of 250 ps. Peptide relative fractional uptakes were corrected for the deuterium uptake of the maximally deuterated control and then extracted using a python script for defined peptide segments for each deuterium labelling time. A list of peptide segments present in the target HDX-MS data was extracted using a custom python script from extracted deuterated fractions data.

HDX data calculation using HDXer

Within HDXer, the Best and Vendruscolo method[28] was used to estimate protection factors based on ensemble structures from MD simulations. The distance cut-offs were set as 0.65 nm and 0.24 nm to count heavy atom contact and hydrogen bonds between backbone amides and surrounding atoms including protein atoms, POPE lipids atoms, N terminus and bound ligand if applicable. The scaling factors are set as default to 0.35 for β_c and 2.0 for β_H. Residue intrinsic exchange rates were

calculated at the experimental conditions, in which pD is 7.0 and temperature is 293 K, with reference acid, base, and water catalysis parameters set to 1.62, 10.18 and -1.50 respectively[29].

Reweighting ensemble experiments

Ensemble reweighting was carried out using the output files of per-residue contacts and H-bonds created by HDX data calculation via HDXer. A mixed candidate ensemble, initially composed of 50% OF and 50% IF structures, was fitted using HDXer to each target HDX-MS dataset with absolute HDX-MS data. The γ value parameter in ensemble reweighting was first explored in a wide range from 1×10^{-1} to 9×10^{2}. A "decision" plot was generated using a custom python script to investigate the effect of the choices of γ value upon the mean-square deviation (MSD) of the fitted data to target experimental data. In each decision plot, overfitting was classified as a rapid increase of the applied apparent work (W_{app}) for limited improvement of MSD. Typically, this occurred at W_{app} over 5 kJ/mol. To adjust the initial ensemble without overfitting and allow equivalent comparison of ensembles reweighted to different datasets, we compared all results at the exact γ value that corresponded as closely as possible to W_{app} of 5 kJ/mol. To investigate the effect of target experimental noise on final ensemble structures, a "leave one out" method was applied, in which peptide segments with high errors are systematically removed from the target data for each round of reweighting.

Supporting Figures

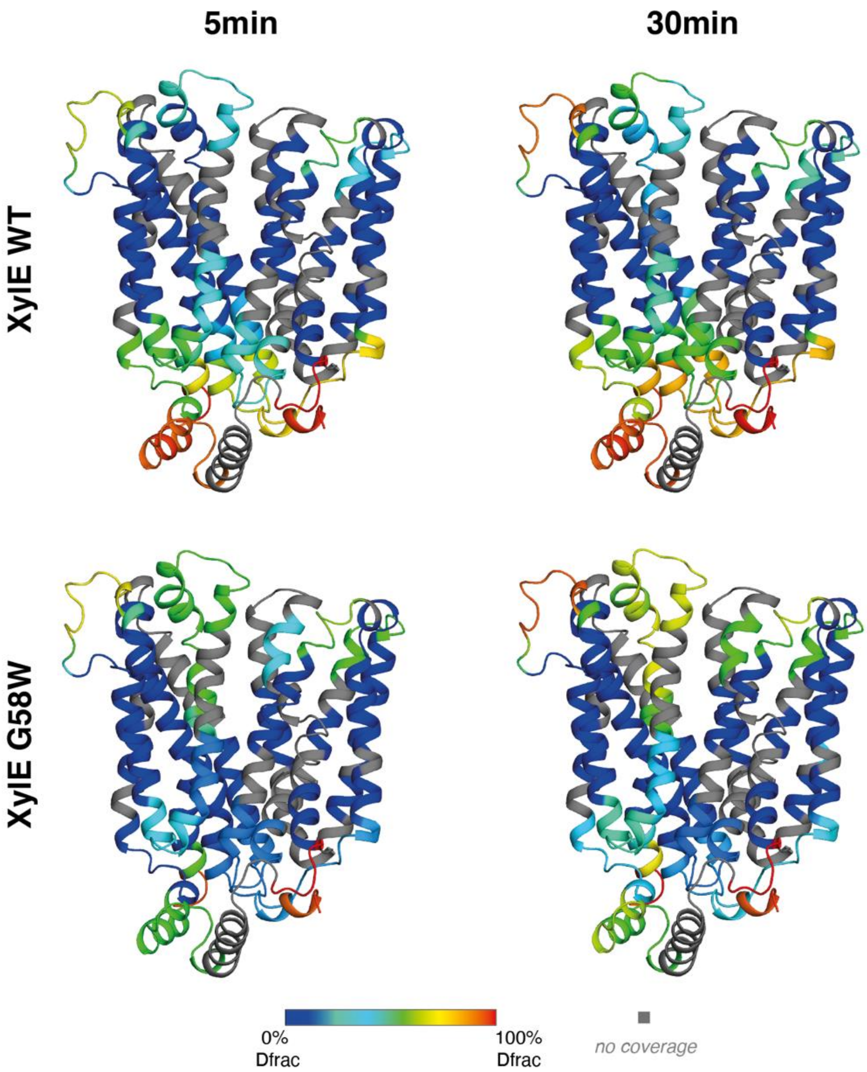

Figure S1 Absolute deuteration of XylE WT and G58W. Peptides are coloured in rainbow scale from 0 to 100% deuteration (purple to red) according to the observed fractional uptake normalized to the MaxD. No-coverage regions are coloured in dark grey. The sequence coverage is 76.4%.

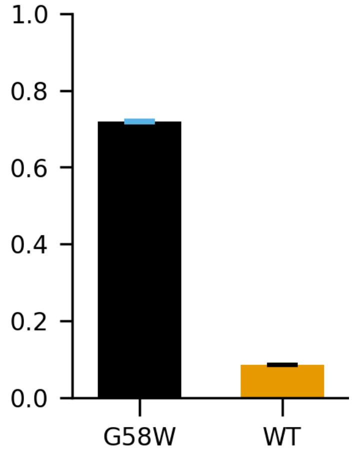

Figure S2 Computational reweighting of a mixed OF/IF ensemble using a parametrized predictive model with β_C = 0.29, β_H = 3.9 still results in a clear separation of the structures present in each experimental dataset. WT XylE remains mostly inward-facing, G58W mostly outward-facing.

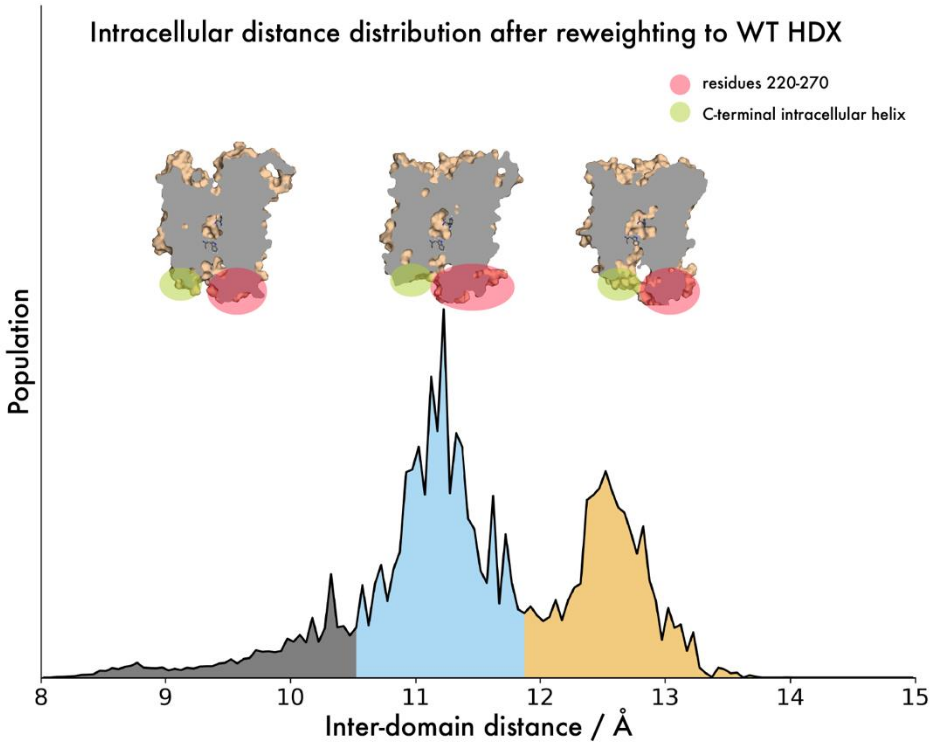

Figure S3 Representative structures (sub-distribution centroids) associated with final ensemble after reweighting to fit WT HDX-MS data. The final ensemble contains substantial populations of both a partially-inward-open (blue cluster) and fully-inward-open (orange cluster) state. The extent of opening, defined by the inter-domain distance, is particularly correlated with the motion of intracellular loops 1-4, visible at the lower right of the structures. Regions cover residues 220-270 and C-terminal intracellular helix are circled in red and green, respectively.

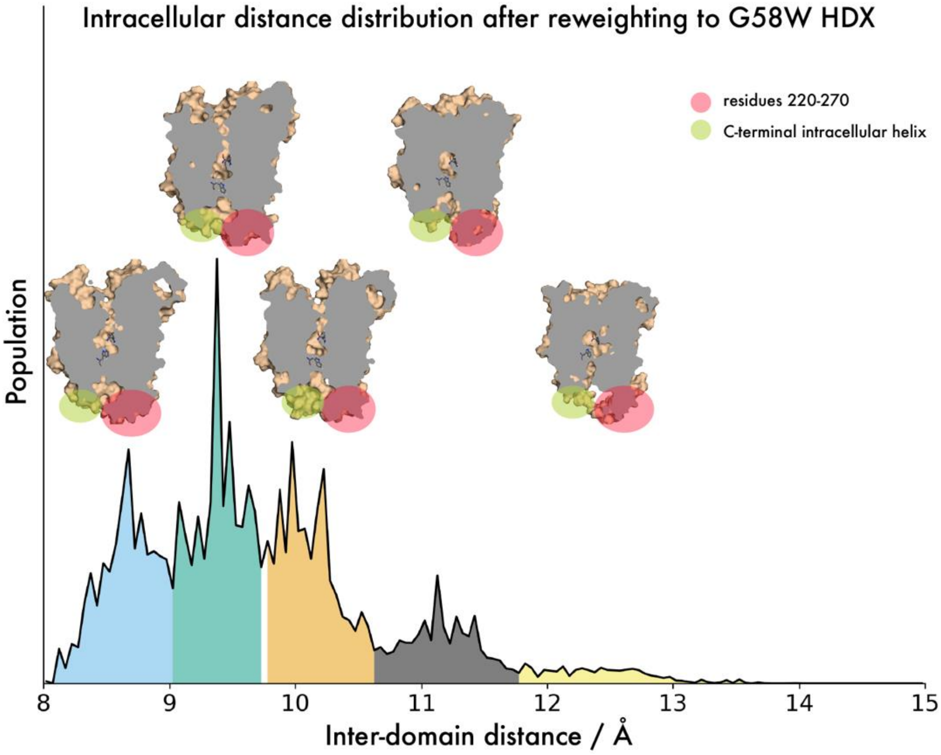

Figure S4 Representative structures (sub-distribution centroids) associated with final ensemble after reweighting to fit G58W HDX-MS data. The final ensemble contains mostly outward-facing populations (blue, green, and orange clusters), with a minor population of a partially inward-open state (black cluster). The fully-inward-open (yellow cluster) state has a negligible population. Outward-facing structures also show flexibility in intracellular loops 1-4, which results in the broad distribution of intracellular inter-domain distances between 8.0 – 10.6 Å. Regions cover residues 220-270 and C-terminal intracellular helix are circled in red and green, respectively.

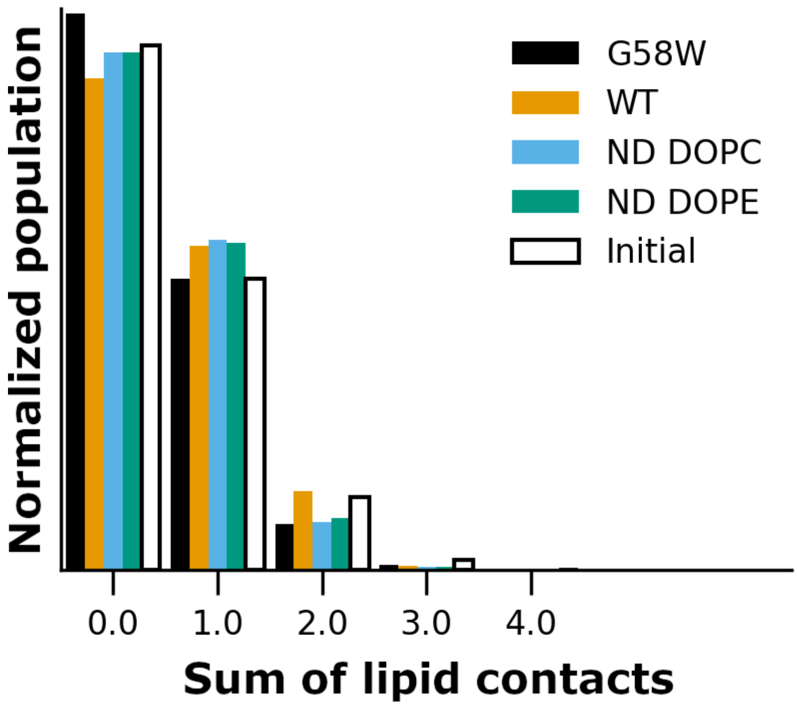

Figure S5 Sum of lipid contacts between the PE headgroup and E153, D337, or E397, before (white) and after reweighting to G58W (black), WT (orange), DOPC nanodisc (blue) or DOPE nanodisc (green) HDX-MS data.

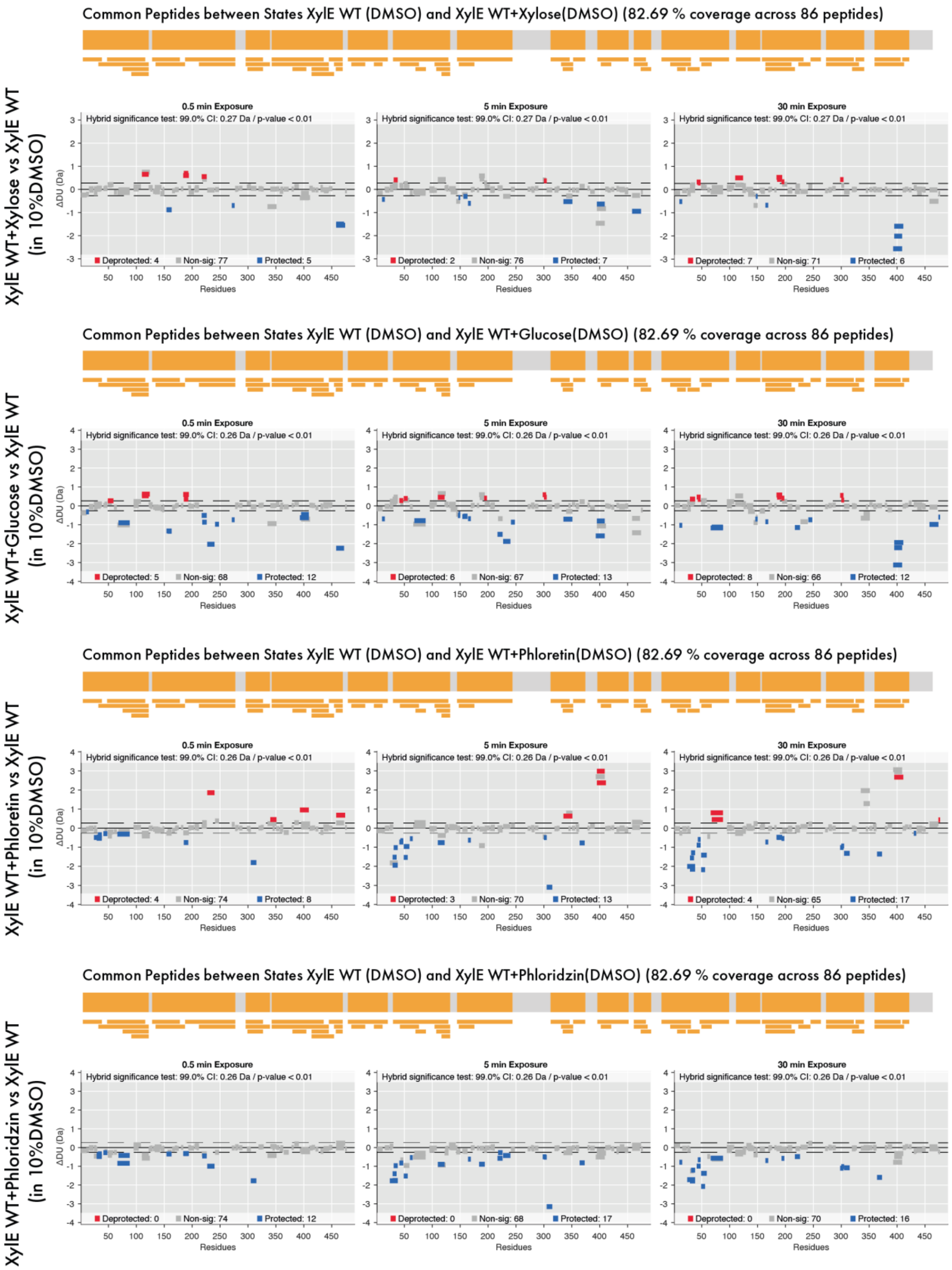

Figure S6 Woods plots and common sequence coverage maps obtained from differential HDX-MS of XylE apo and ligand-bound states. Each bar represents a single peptide with peptide length indicated by the bar length. Common peptides between two different protein states are indicated as orange.

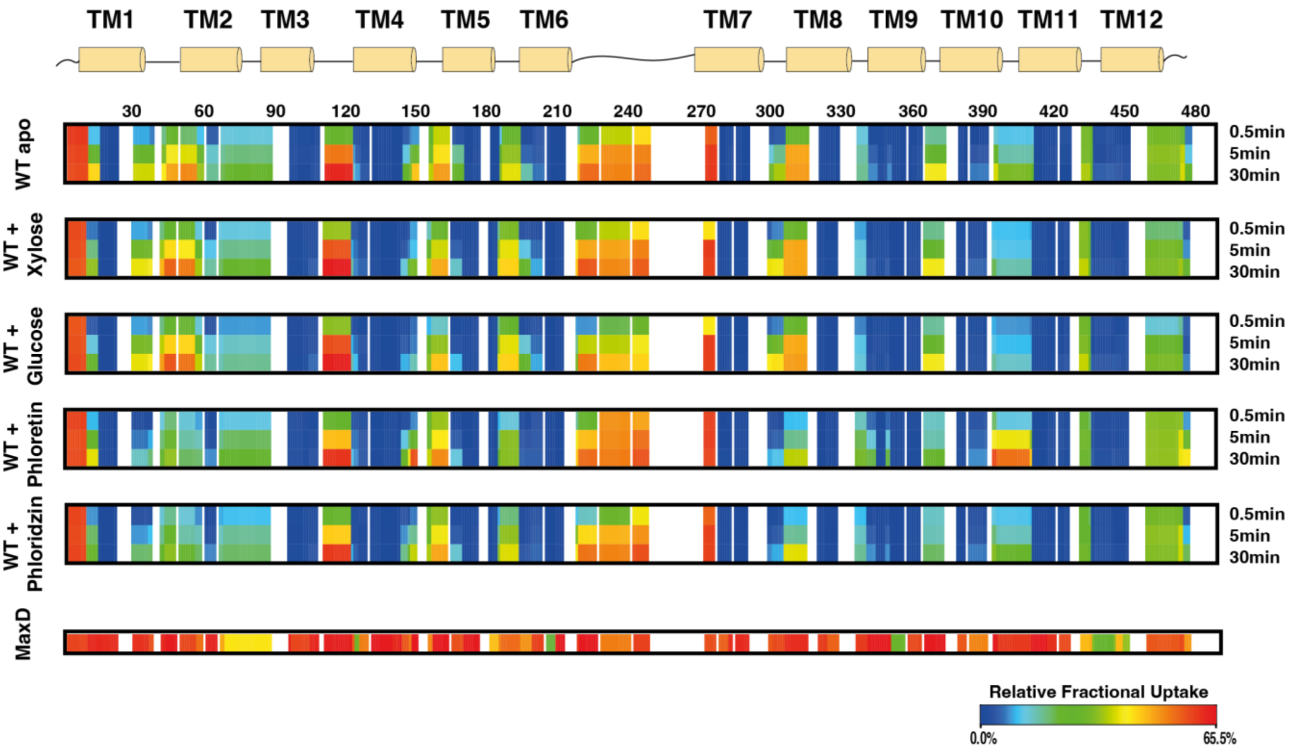

Figure S7 Heatmap of all protein states in triplicates. Protein states include XylE apo, WT + xylose, WT + glucose, WT + phloretin, WT + phloridzin and maximally deuterated control (MaxD).

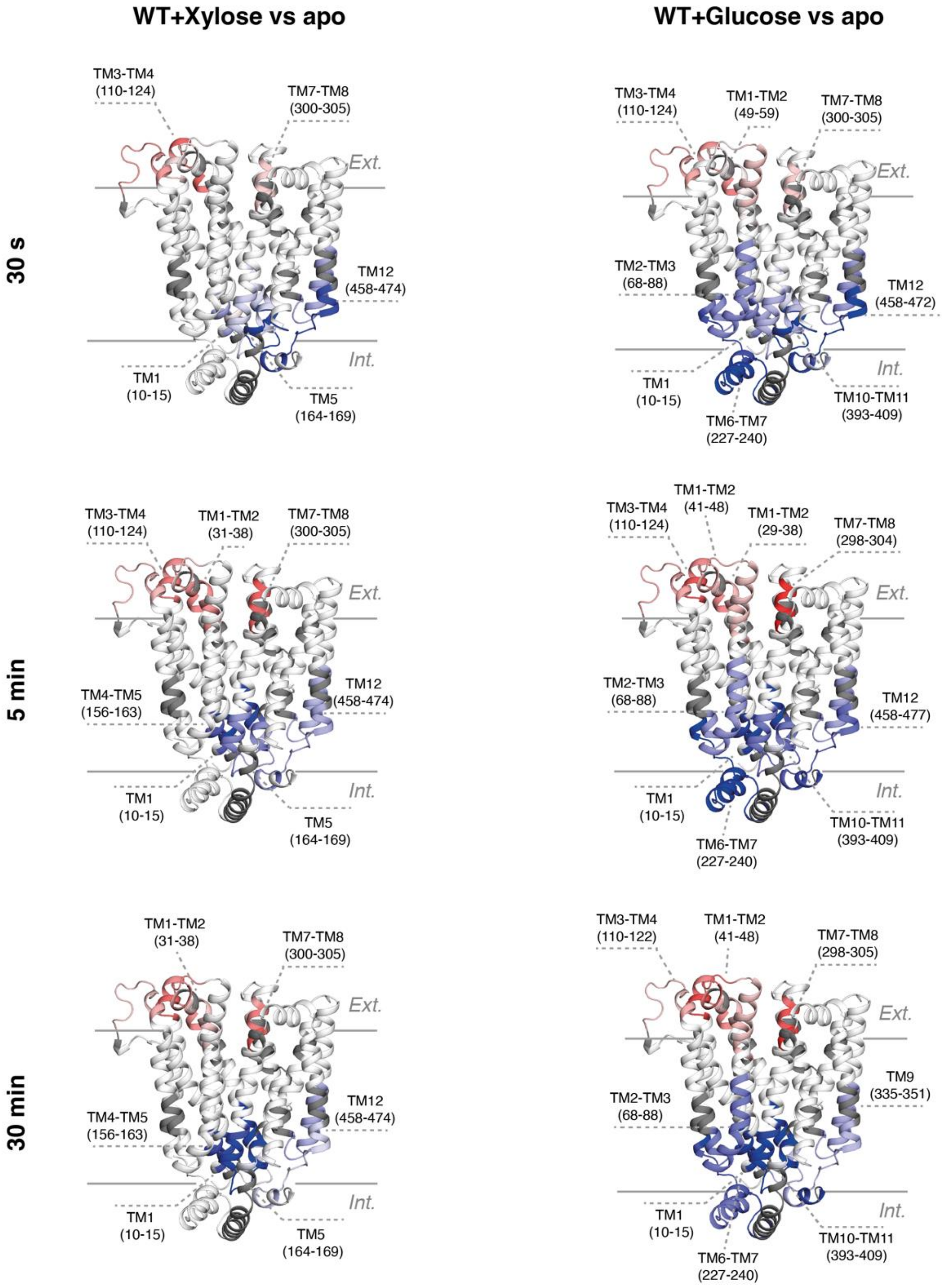
WT+Xylose vs apo
WT+Glucose vs apo
30 s
5 min
30 min
TM3-TM4
(110-124)
TM7-TM8
(300-305)
Ext.
TM12
(458-474)
TM1
(10-15)
Int.
TM5
(164-169)
TM3-TM4
(110-124)
TM1-TM2
(49-59)
TM7-TM8
(300-305)
Ext.
TM2-TM3
(68-88)
TM12
(458-472)
TM1
(10-15)
Int.
TM6-TM7
(227-240)
TM10-TM11
(393-409)
TM3-TM4
(110-124)
TM1-TM2
(31-38)
TM7-TM8
(300-305)
Ext.
TM4-TM5
(156-163)
TM12
(458-474)
TM1
(10-15)
Int.
TM5
(164-169)
TM1-TM2
(41-48)
TM3-TM4
(110-124)
TM1-TM2
(29-38)
TM7-TM8
(298-304)
Ext.
TM2-TM3
(68-88)
TM12
(458-477)
TM1
(10-15)
Int.
TM6-TM7
(227-240)
TM10-TM11
(393-409)
TM1-TM2
(31-38)
TM7-TM8
(300-305)
Ext.
TM4-TM5
(156-163)
TM12
(458-474)
TM1
(10-15)
Int.
TM5
(164-169)
TM3-TM4
(110-122)
TM1-TM2
(41-48)
TM7-TM8
(298-305)
Ext.
TM2-TM3
(68-88)
TM9
(335-351)
TM1
(10-15)
Int.
TM6-TM7
(227-240)
TM10-TM11
(393-409)

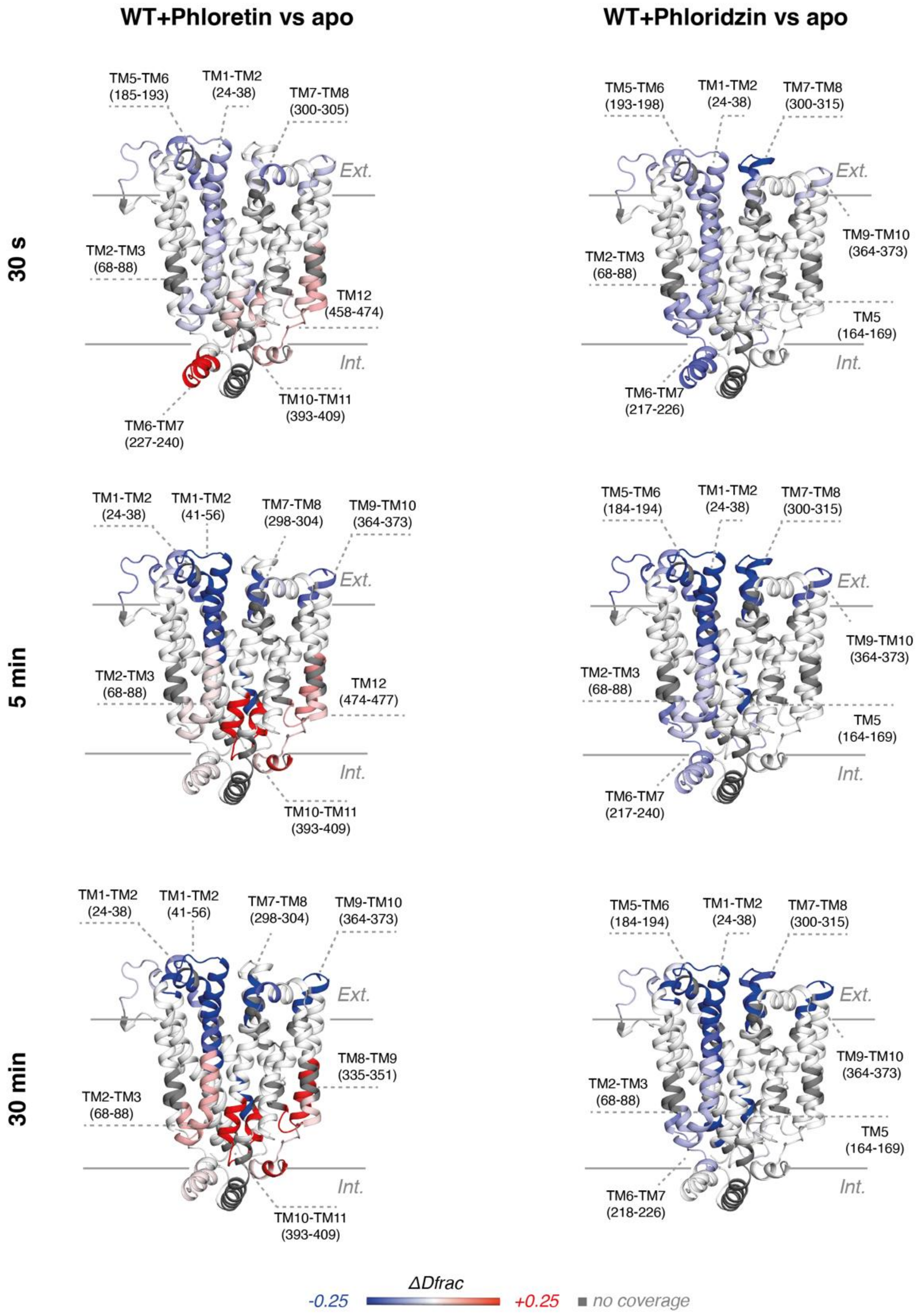

Figure S8 Differential deuterium uptake plotted onto 3D protein structure (PDB: 4GBY) at 30s, 5min and 30min for all protein states. Protein states include XylE apo, WT + xylose, WT + glucose, WT + phloretin and WT + phloridzin. Regions showing a difference in HDX compared to the apo state are coloured in blue (protected) or red (deprotected) scale according to the difference in fractional uptake normalized to the MaxD. No-coverage regions are coloured in dark grey.

a

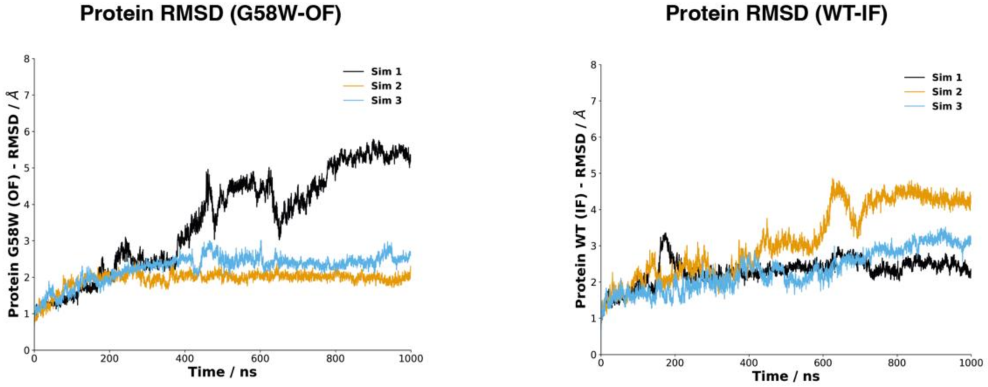
Protein RMSD (G58W-OF)
Protein G58W (OF) - RMSD / Å
Time / ns
Sim 1
Sim 2
Sim 3
Protein RMSD (WT-IF)
Protein WT (IF) - RMSD / Å
Time / ns
Sim 1
Sim 2
Sim 3

b

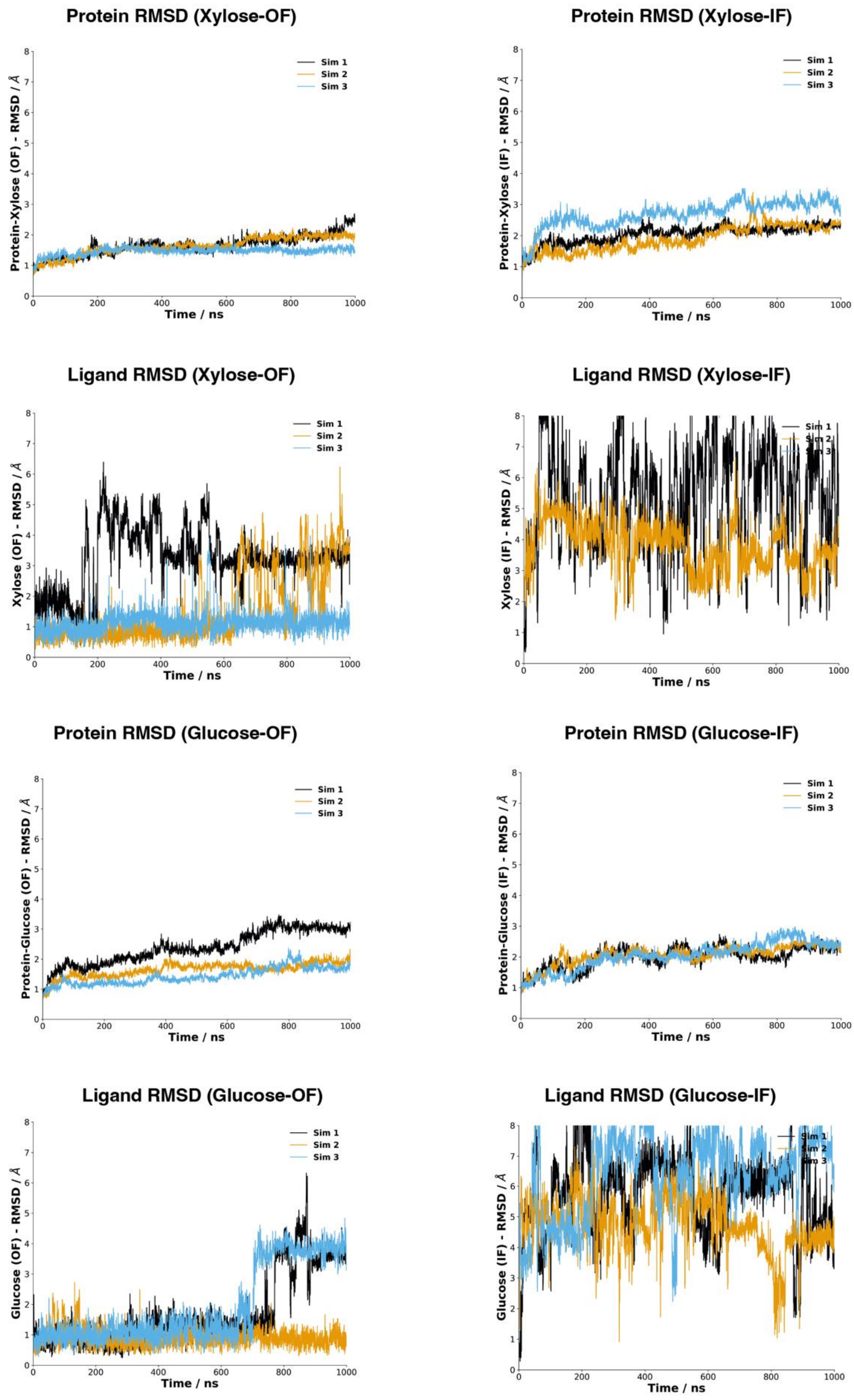
Protein RMSD (Xylose-OF)
Protein-Xylose (OF) - RMSD / Å
Time / ns
Sim 1
Sim 2
Sim 3
Protein RMSD (Xylose-IF)
Protein-Xylose (IF) - RMSD / Å
Time / ns
Sim 1
Sim 2
Sim 3
Ligand RMSD (Xylose-OF)
Xylose (OF) - RMSD / Å
Time / ns
Sim 1
Sim 2
Sim 3
Ligand RMSD (Xylose-IF)
Xylose (IF) - RMSD / Å
Time / ns
Protein RMSD (Glucose-OF)
Protein-Glucose (OF) - RMSD / Å
Time / ns
Sim 1
Sim 2
Sim 3
Protein RMSD (Glucose-IF)
Protein-Glucose (IF) - RMSD / Å
Time / ns
Sim 1
Sim 2
Sim 3
Ligand RMSD (Glucose-OF)
Glucose (OF) - RMSD / Å
Time / ns
Sim 1
Sim 2
Sim 3
Ligand RMSD (Glucose-IF)
Glucose (IF) - RMSD / Å
Time / ns

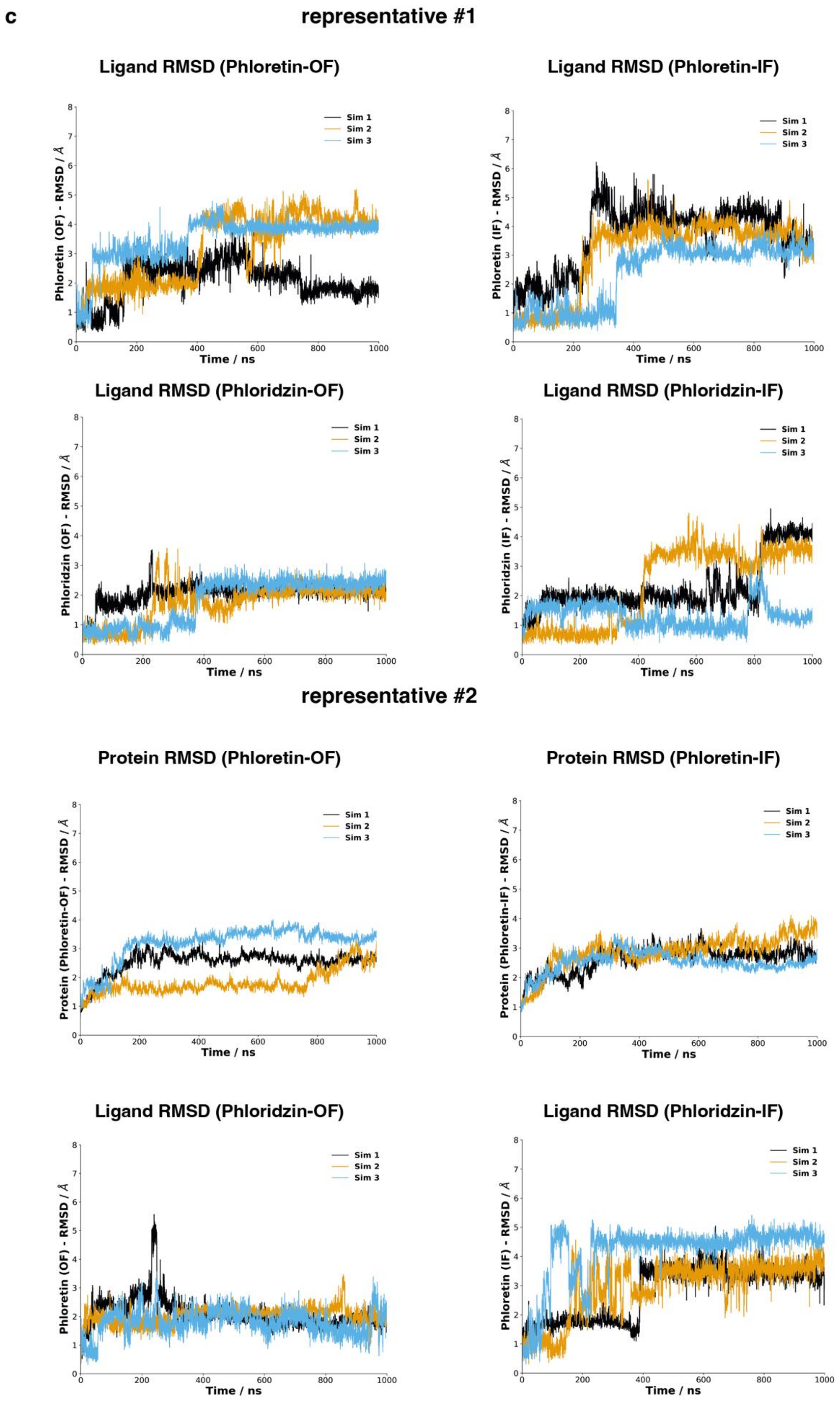
c
representative #1
Ligand RMSD (Phloretin-OF)
Phloretin (OF) - RMSD / Å
Time / ns
Sim 1
Sim 2
Sim 3
Ligand RMSD (Phloretin-IF)
Phloretin (IF) - RMSD / Å
Time / ns
Sim 1
Sim 2
Sim 3
Ligand RMSD (Phloridzin-OF)
Phloridzin (OF) - RMSD / Å
Time / ns
Sim 1
Sim 2
Sim 3
Ligand RMSD (Phloridzin-IF)
Phloridzin (IF) - RMSD / Å
Time / ns
Sim 1
Sim 2
Sim 3
representative #2
Protein RMSD (Phloretin-OF)
Protein (Phloretin-OF) - RMSD / Å
Time / ns
Sim 1
Sim 2
Sim 3
Protein RMSD (Phloretin-IF)
Protein (Phloretin-IF) - RMSD / Å
Time / ns
Sim 1
Sim 2
Sim 3
Ligand RMSD (Phloridzin-OF)
Phloretin (OF) - RMSD / Å
Time / ns
Sim 1
Sim 2
Sim 3
Ligand RMSD (Phloridzin-IF)
Phloretin (IF) - RMSD / Å
Time / ns
Sim 1
Sim 2
Sim 3

Figure S9 RMSD plots of protein and ligand structures in OF and IF conformation over the 1 μs simulation time. (**a**)apo state (4GBY and 4JA4). (**b**) Xylose (4GBY and 4JA4)-, glucose (4GBZ and 4JA4)-bound states. (**c**) Phloretin (4GBY and 4JA4) phloridzin (4GBY and 4JA4))-bound states. Simulations of (**a**) and (**b**) were generated using crystal structures directly or docked ligands into crystal structures, and (**c**) were generated by docking ligands into representative MD structures. Three independent simulation runs are shown in black, orange and blue, respectively.

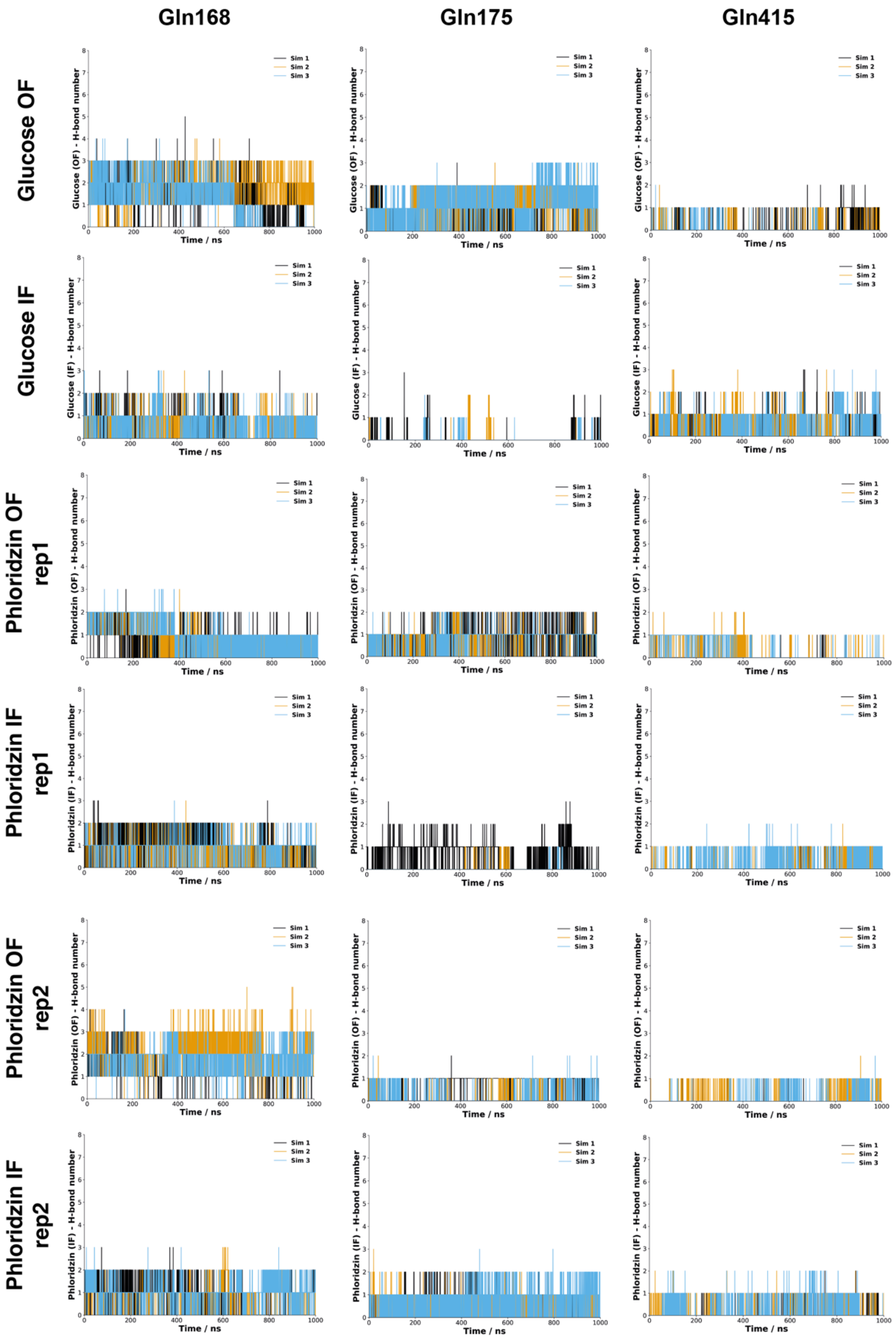
Gln168
Gln175
Gln415
Glucose OF
Glucose IF
Phloridzin OF rep1
Phloridzin IF rep1
Phloridzin OF rep2
Phloridzin IF rep2
Sim 1
Sim 2
Sim 3
Glucose (OF) - H-bond number
Glucose (IF) - H-bond number
Phloridzin (OF) - H-bond number
Phloridzin (IF) - H-bond number
Time / ns

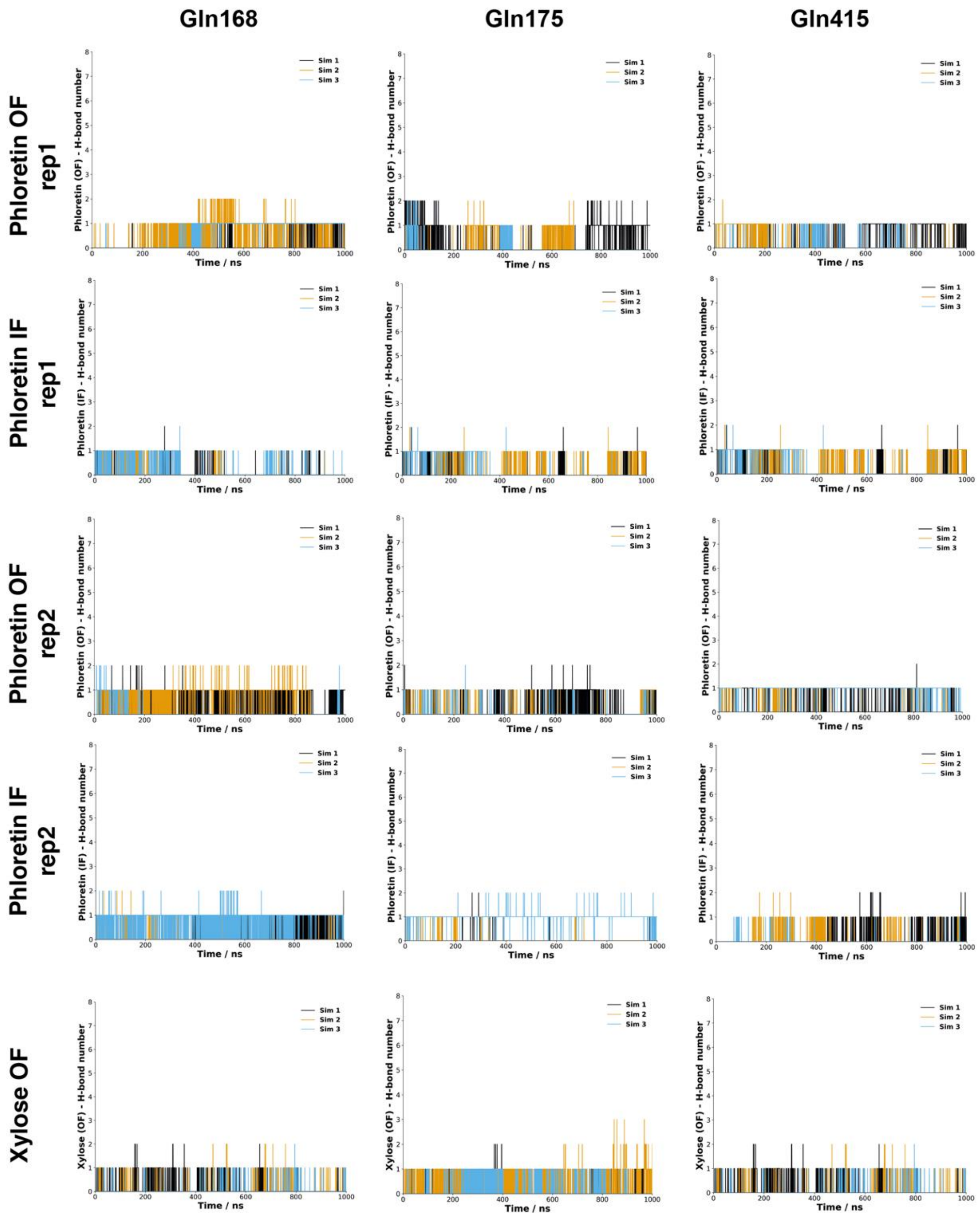

Figure S10 Hydrogen bond interaction analysis for glucose-, phloridzin-, phloretin-, and xylose-bound structures. The number of hydrogen bond interactions between ligand (glucose, phloridzin, phloretin and xylose) with residue Gln168, Gln175 and Gln415 over simulation time were plotted separately for three independent simulations in outward and inward-facing conformation. Xylose was dissociated from xylose-bound structures in inward-facing conformation from MD simulations, therefore, it was excluded from the H-bond analysis.

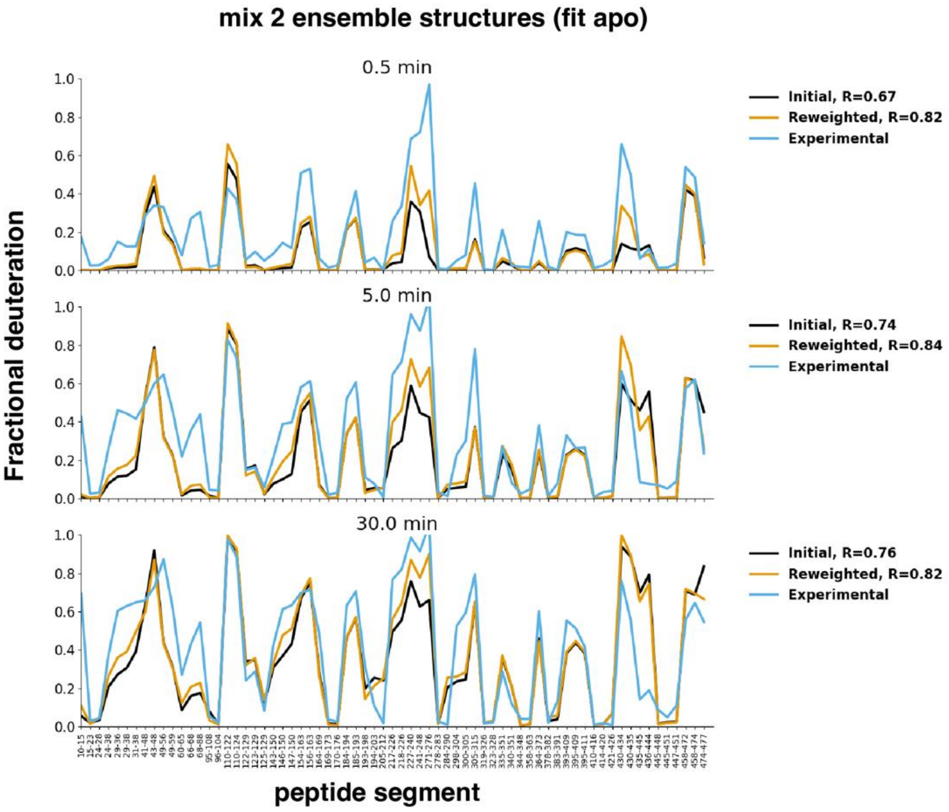
mix 2 ensemble structures (fit apo)
0.5 min
5.0 min
30.0 min
Fractional deuteration
peptide segment
Initial, R=0.67
Reweighted, R=0.82
Experimental
Initial, R=0.74
Reweighted, R=0.84
Experimental
Initial, R=0.76
Reweighted, R=0.82
Experimental

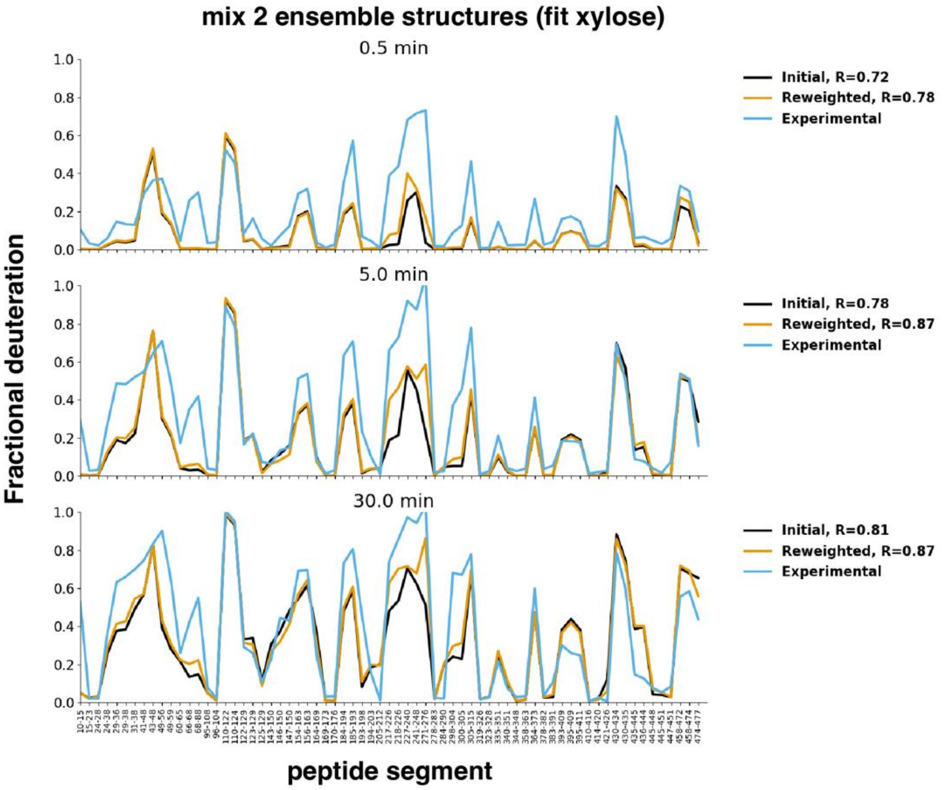
mix 2 ensemble structures (fit xylose)
0.5 min
5.0 min
30.0 min
Fractional deuteration
peptide segment
Initial, R=0.72
Reweighted, R=0.78
Experimental
Initial, R=0.78
Reweighted, R=0.87
Experimental
Initial, R=0.81
Reweighted, R=0.87
Experimental

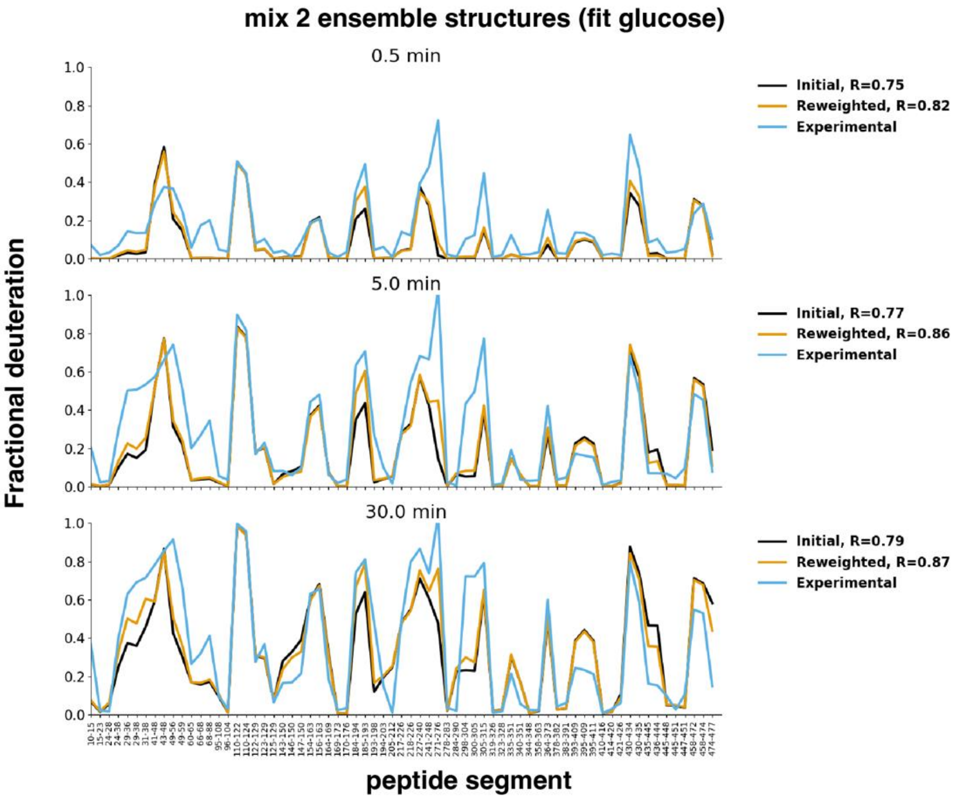
mix 2 ensemble structures (fit glucose)
0.5 min
Initial, R=0.75
Reweighted, R=0.82
Experimental
5.0 min
Initial, R=0.77
Reweighted, R=0.86
Experimental
30.0 min
Initial, R=0.79
Reweighted, R=0.87
Experimental
Fractional deuteration
peptide segment

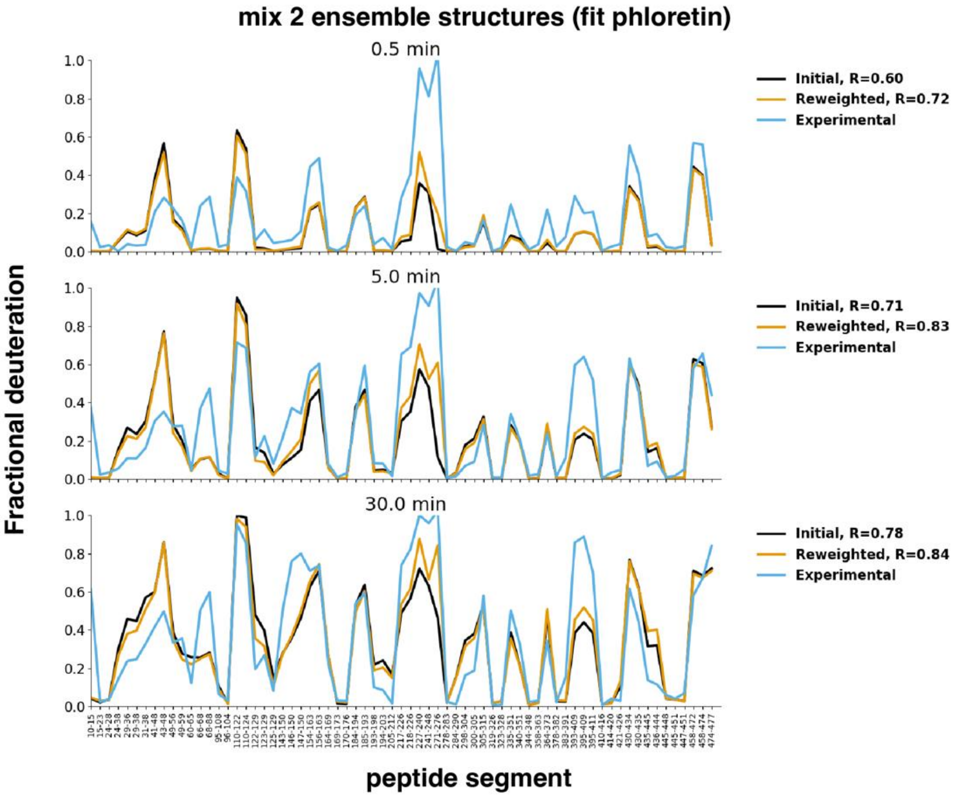
mix 2 ensemble structures (fit phloretin)
0.5 min
Initial, R=0.60
Reweighted, R=0.72
Experimental
5.0 min
Initial, R=0.71
Reweighted, R=0.83
Experimental
30.0 min
Initial, R=0.78
Reweighted, R=0.84
Experimental
Fractional deuteration
peptide segment

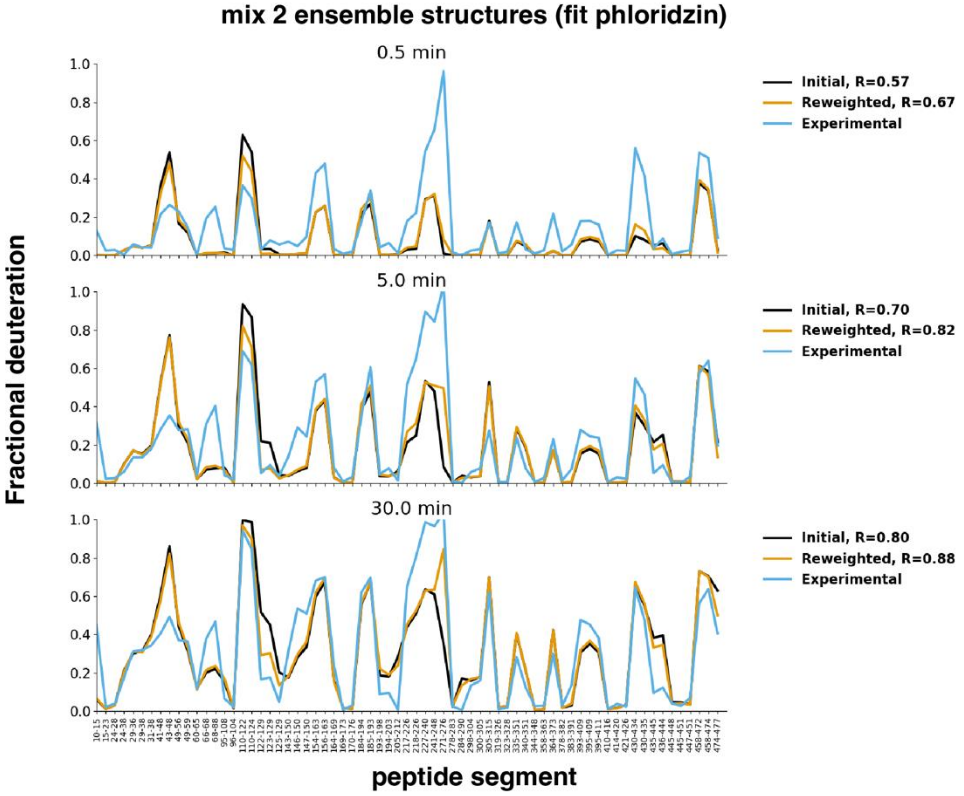

Figure S11 Fractional deuteration before and after reweighting. Fractional deuteration for initial predicted, reweighted and experimental HDX data were shown in black, orange and blue, respectively.

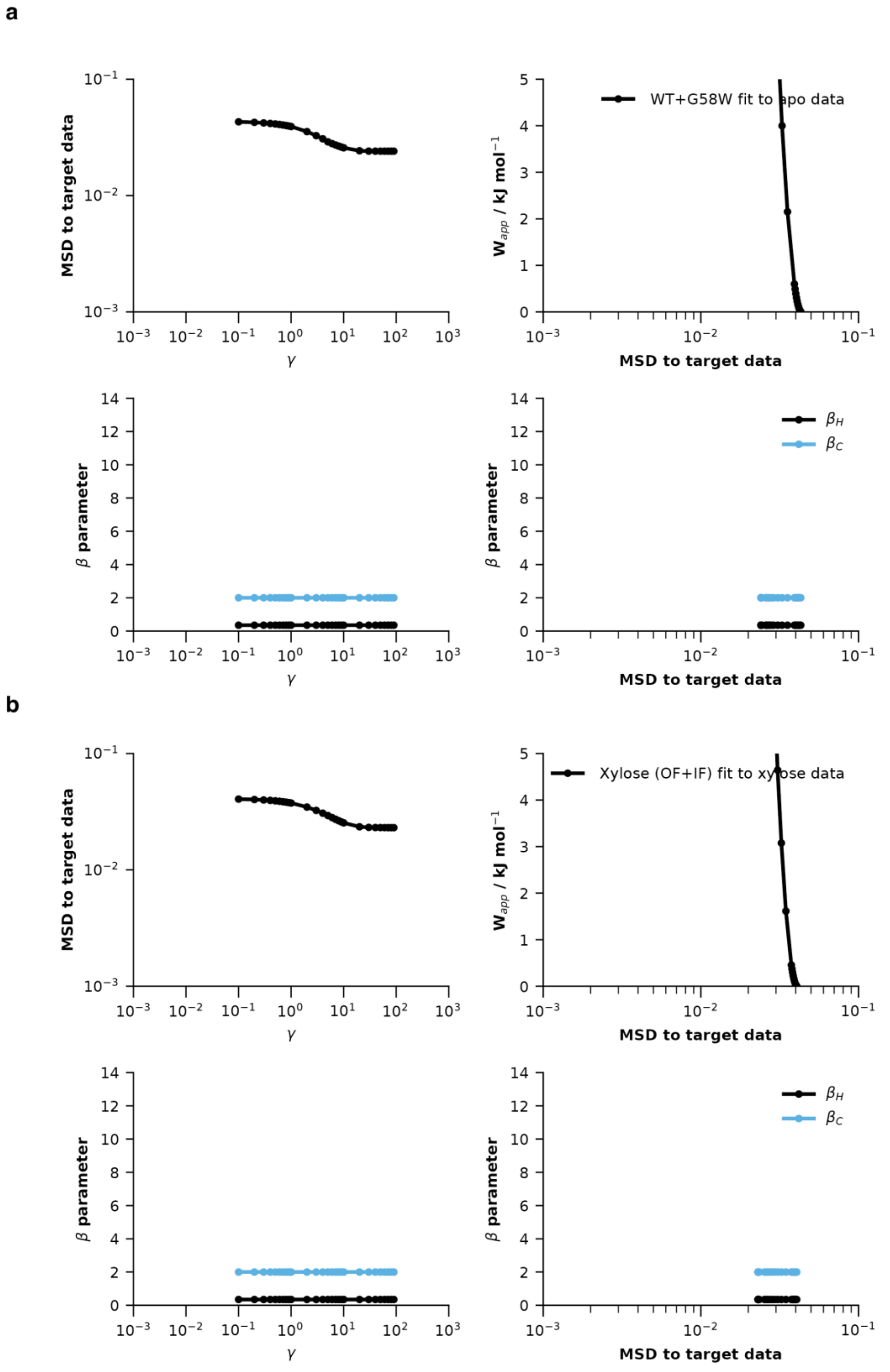
a
b
MSD to target data
γ
WT+G58W fit to apo data
Xylose (OF+IF) fit to xylose data
W_app / kJ mol⁻¹
β parameter
β_H
β_C

c

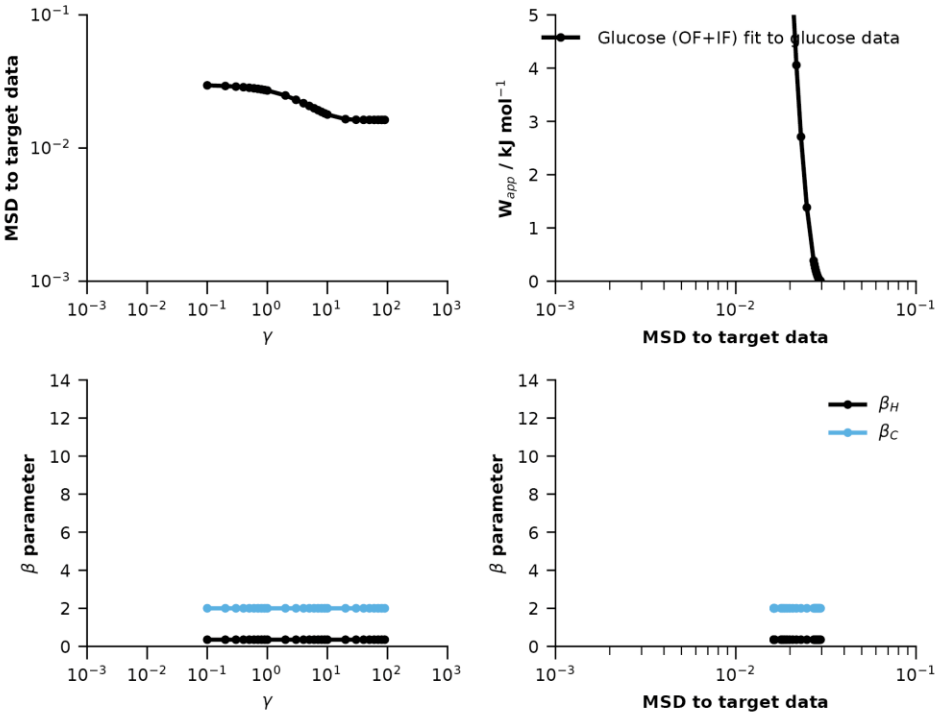
MSD to target data
γ
Glucose (OF+IF) fit to glucose data
W$_{app}$ / kJ mol^{-1}
MSD to target data
β parameter
β_H
β_C

d

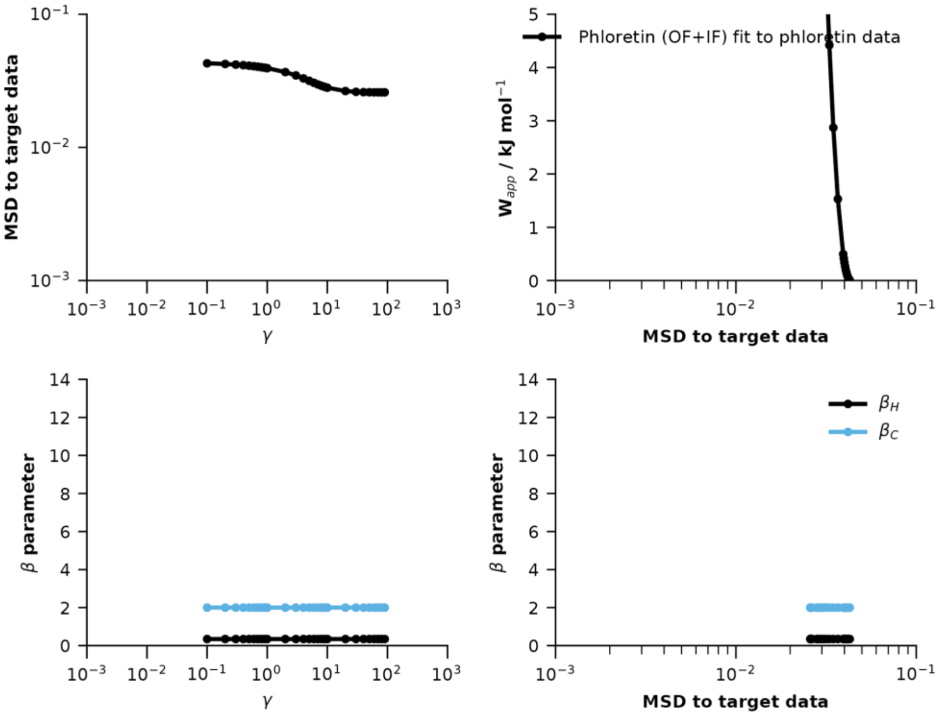
MSD to target data
γ
Phloretin (OF+IF) fit to phloretin data
W$_{app}$ / kJ mol^{-1}
MSD to target data
β parameter
β_H
β_C

e

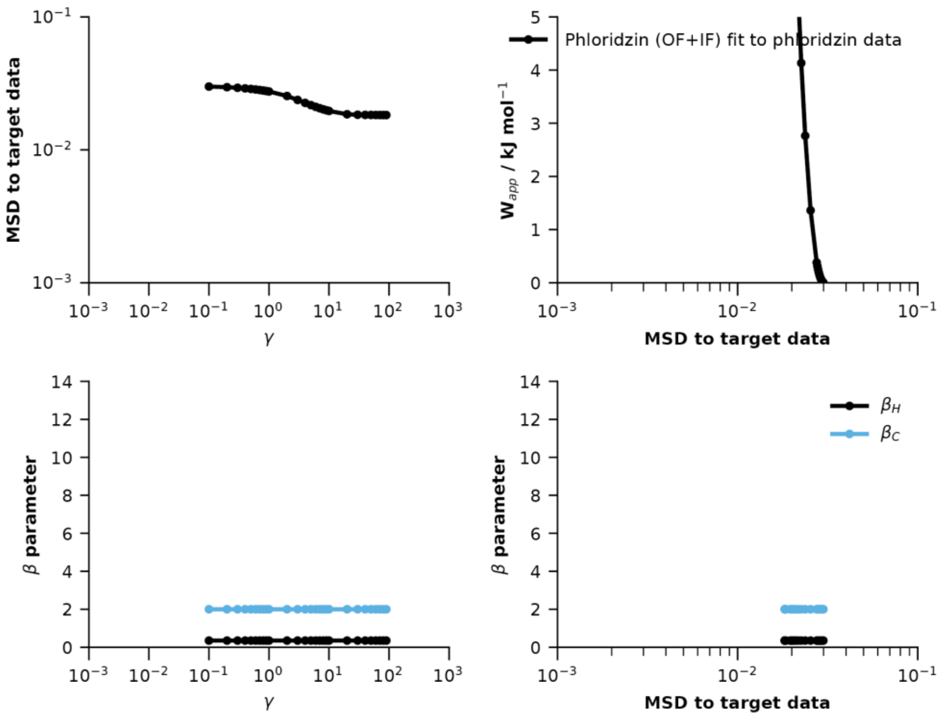

Figure S12 Decision plots on ensemble reweighting. Ensemble reweighting was carried out by fitting ensemble structures (**a**) WT and G58W, (**b**) Xylose-bound (OF and IF), (**c**) Glucose-bound (OF and IF), (**d**) Phloretin-bound (OF and IF) and (**e**)Phloridzin-bound (OF and IF) to experimental XylE (**a**) apo, (**b**) Xylose-, (**c**) Glucose-, (**d**) Phloretin- and (**e**) Phloridzin-bound data, respectively. Top left: Relationship between mean-square deviation (MSD) to target data and γ. Top right: Relationship between applied work (kJ/mol) and MSD to target data. Bottom left: Relationship between scaling factor (β_H andβ_C) and γ. Bottom right: Relationship between scaling factor (β_H andβ_C) and MSD to target data.

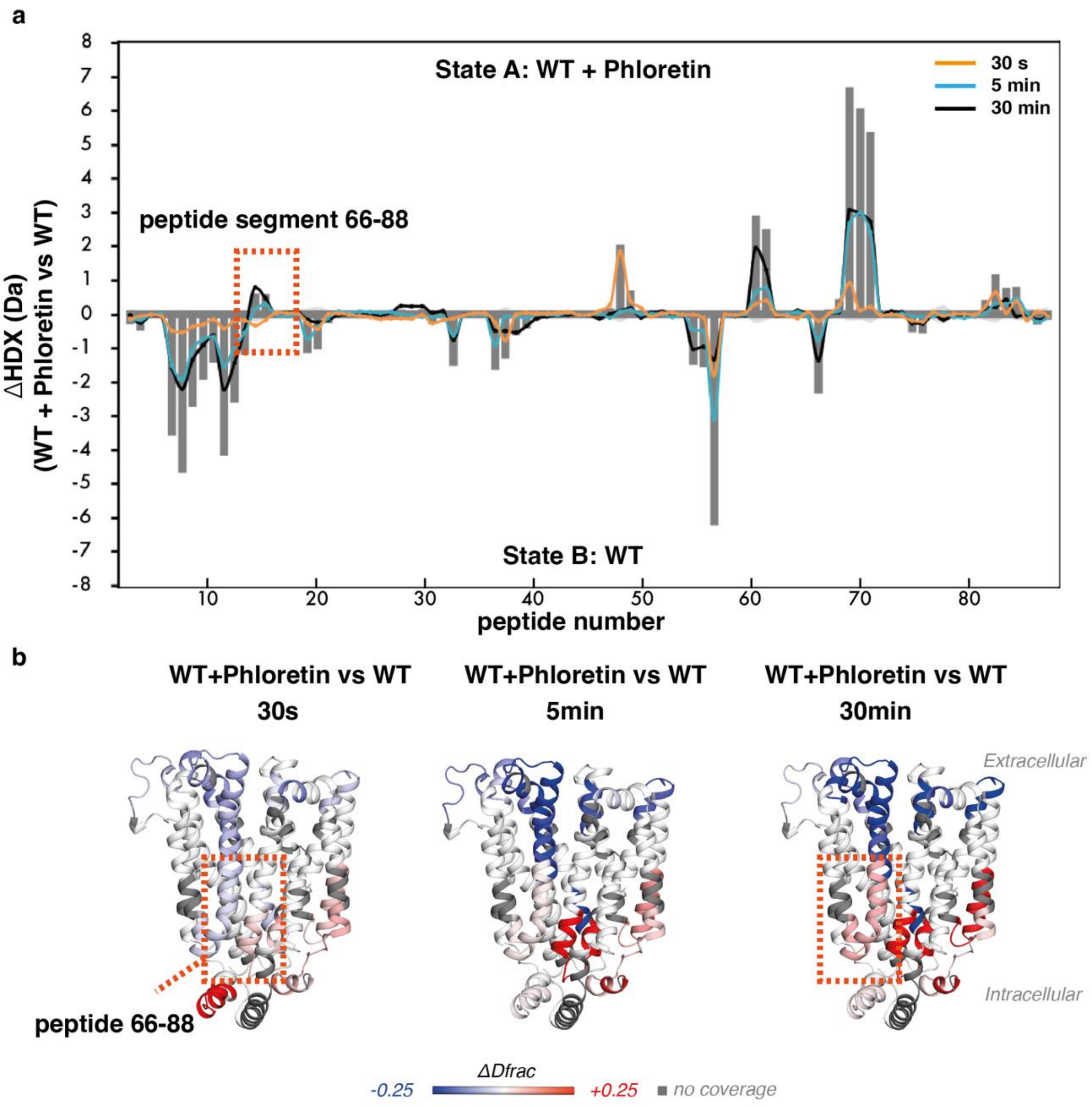

Figure S13 Representation for differential HDX-MS between phloretin-bound and WT structures. (**a**) Difference plot. Orange, cyan and black represent different time points (30 s, 5 min, and 30 min). The grey bars represent the sum of differences over all timepoints. (**b**) Deuterium uptake difference plot on 3D protein structure. Peptide 66-88 presents different HDX patterns at the 30 s and 30 min time points. Regions showing a difference in HDX compared to the apo state are coloured in blue (protected) or red (deprotected) scale according to the difference in fractional uptake normalized to the MaxD. No-coverage regions were coloured in dark grey.

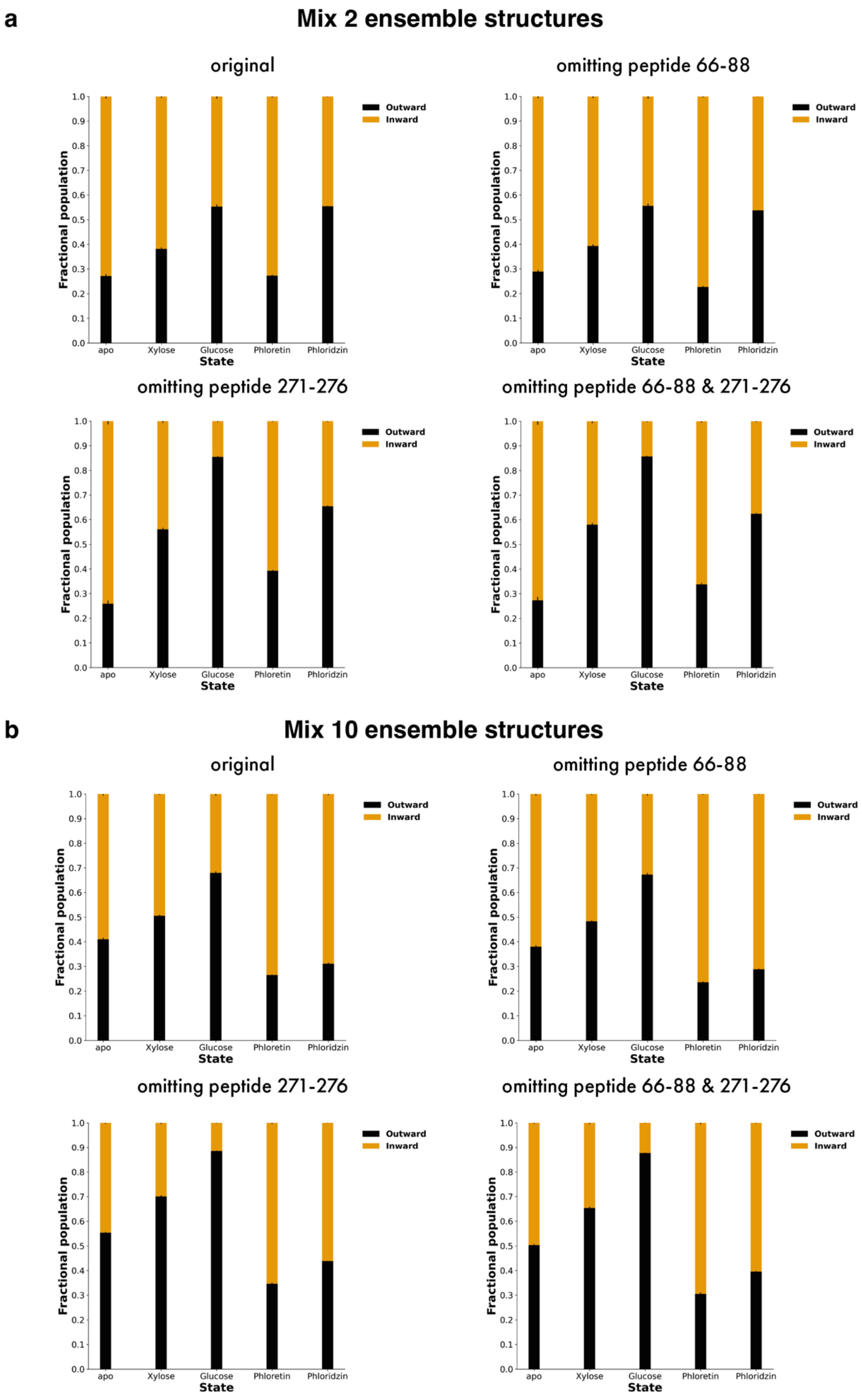
a
Mix 2 ensemble structures
original
omitting peptide 66-88
omitting peptide 271-276
omitting peptide 66-88 & 271-276
b
Mix 10 ensemble structures
original
omitting peptide 66-88
omitting peptide 271-276
omitting peptide 66-88 & 271-276
Fractional population
State
apo
Xylose
Glucose
Phloretin
Phloridzin
Outward
Inward

c Mix 14 ensemble structures

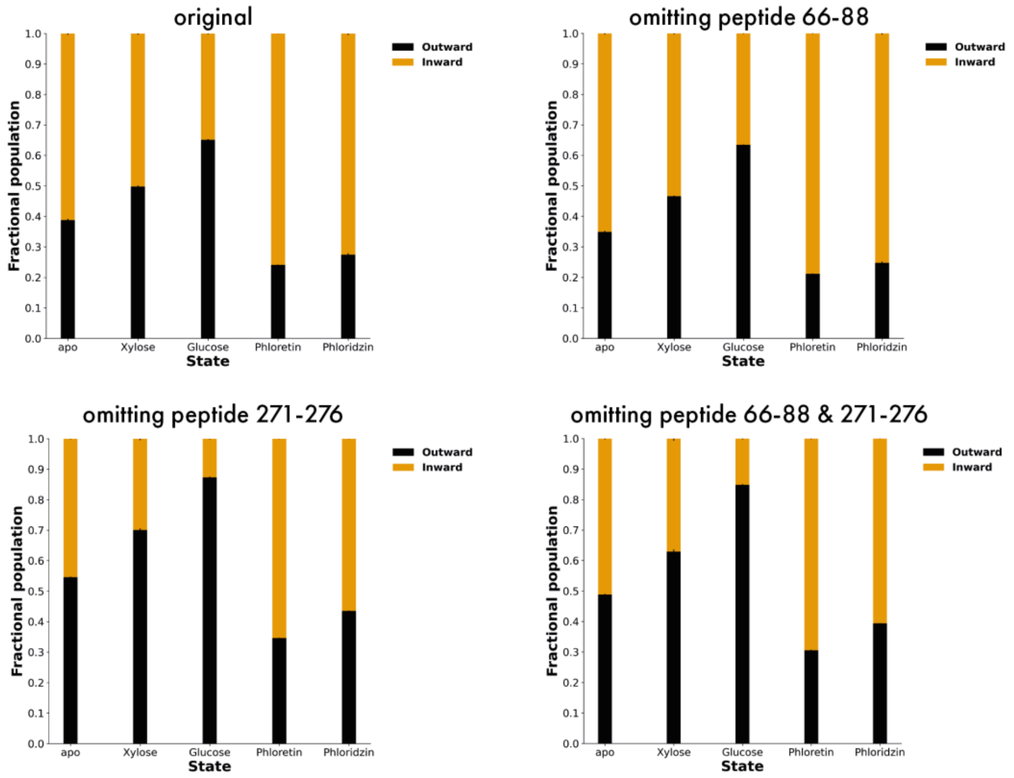

d Compare representative ensemble structures (Phloretin/Phloridzin)

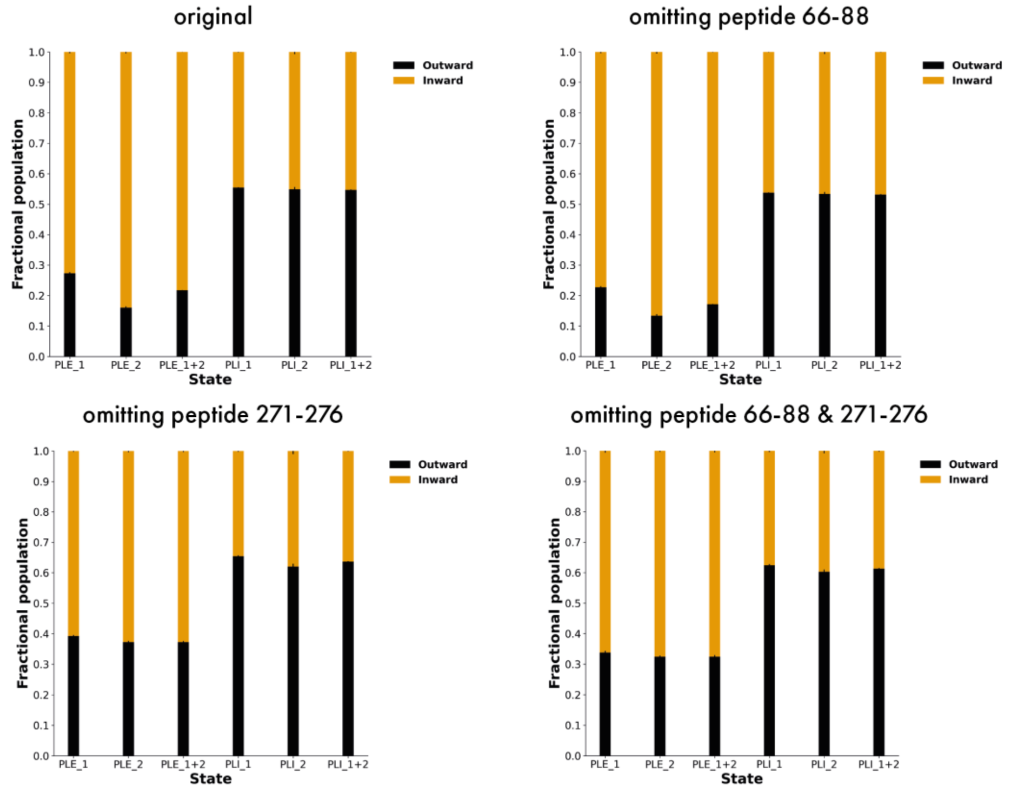

Figure S14 Bar charts of fractional population after reweighting under 4 conditions (original, omitting peptide 66-88, peptides 271-276 or both). (**a**) Mix 2 ensemble structures. (**b**) Mix 10 ensemble structures. (**c**) Mix 14 ensemble structures. (**d**) Comparing fractional population using ensemble structures from alternative initial poses for phloretin and phloridzin (representative structures 1, 2 and both).

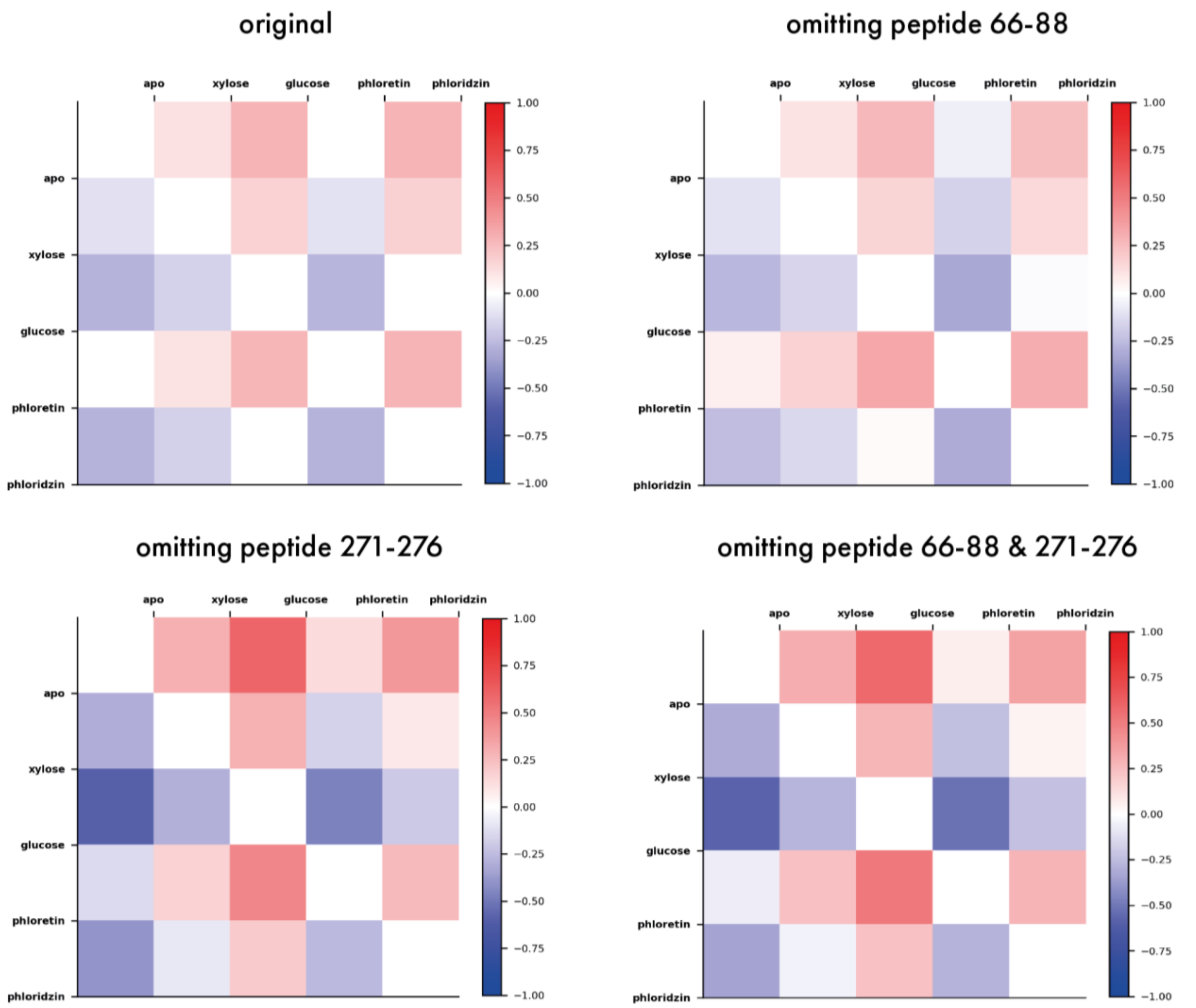

Figure S15 Heap map of relative fractional population of mixing 2 ("state-specific") states between WT and ligand-bound states under four conditions (original, omitting peptide 66-88, peptides 271-276 or both) for mixing 2 ensemble structures. Red indicates relatively outward-facing, and blue indicates relatively inward-facing.

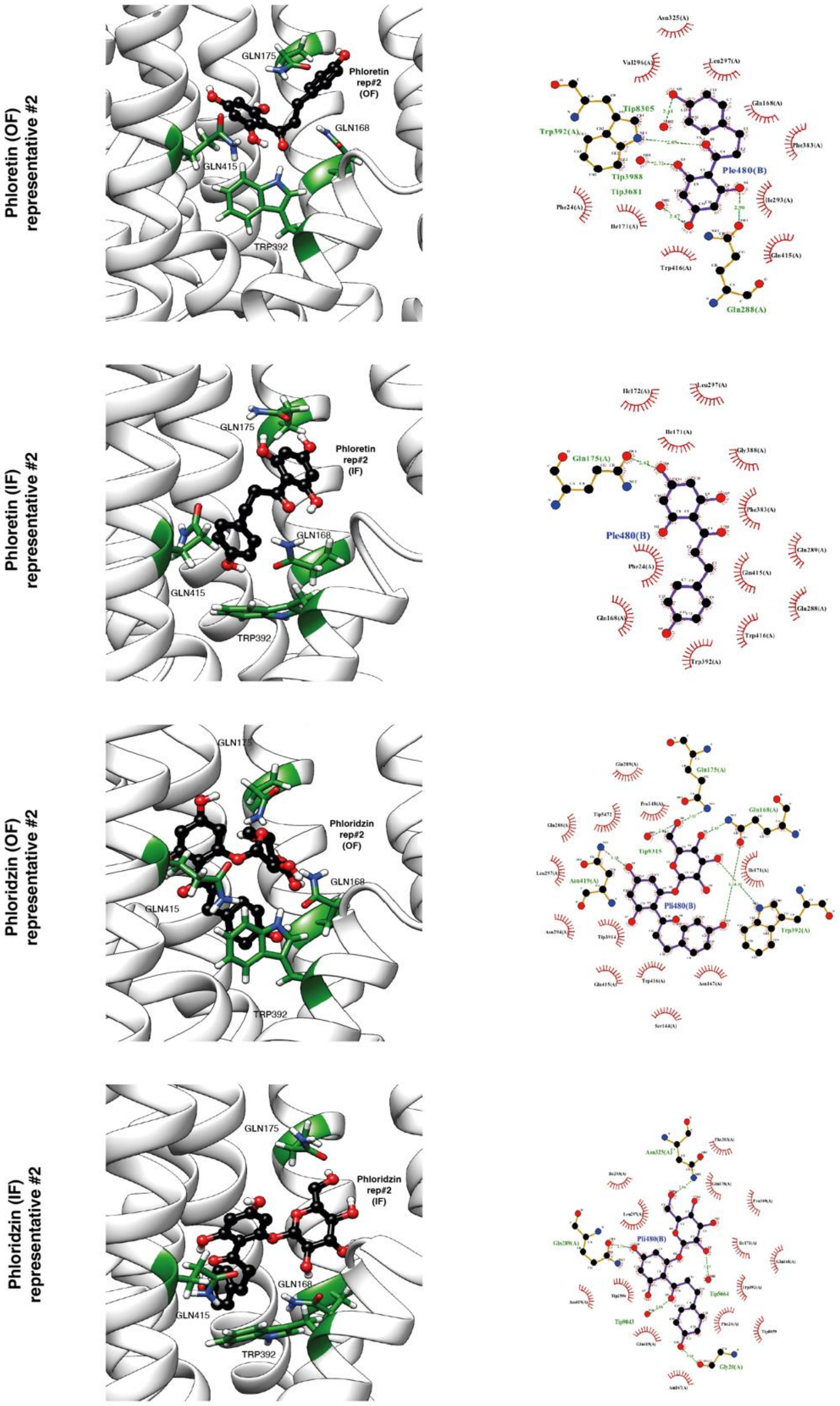

Figure S16 Coordination of second representative phloretin and phloridzin by XylE in outward-facing and inward-facing structures and 2D protein-ligand interaction diagram. Phloretin and phloridzin were shown in black balls and sticks. The binding site residues in XylE were coloured green. The protein-ligand interaction plots were generated by LigPlot+[30]. Hydrogen bonds were shown as green dotted lines, hydrophobic interactions were represented by red eyelashes.

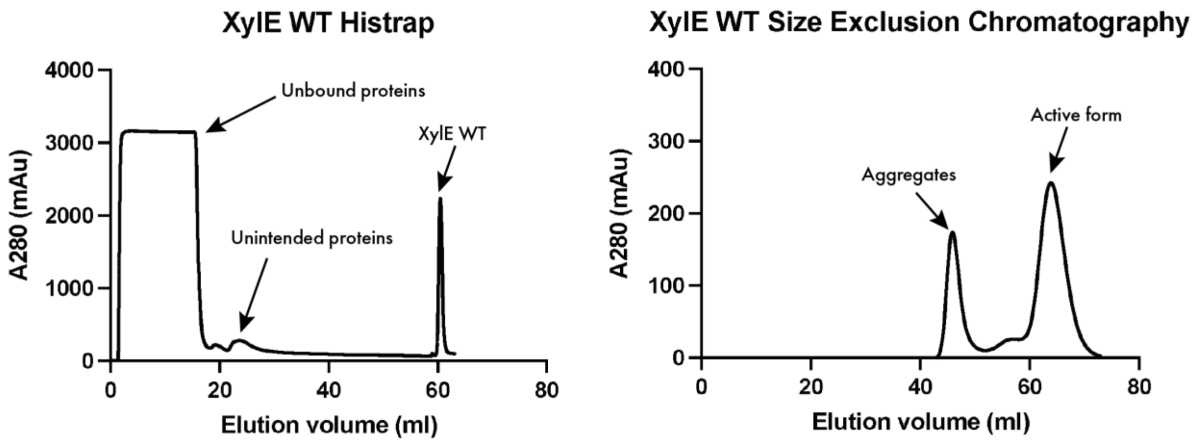

Figure S17 XylE WT chromatogram of Histrap and Size Exclusion Chromatography.

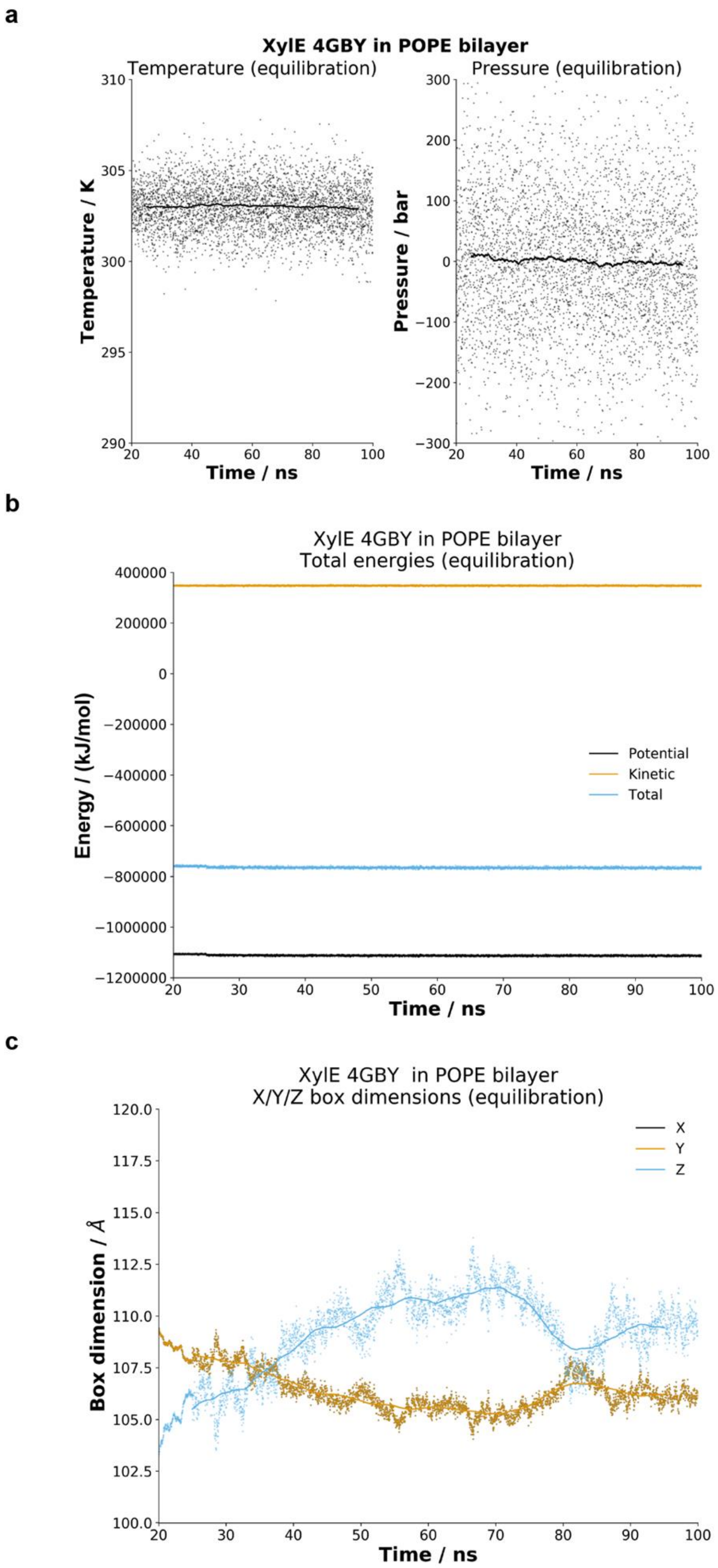

Figure S18 Trajectory analysis of MD simulations at the equilibrium stage. (**a**) Temperature and pressure fluctuate over time. (**b**)Potential, kinetic and total energy over simulation time. (**c**) Box dimension.

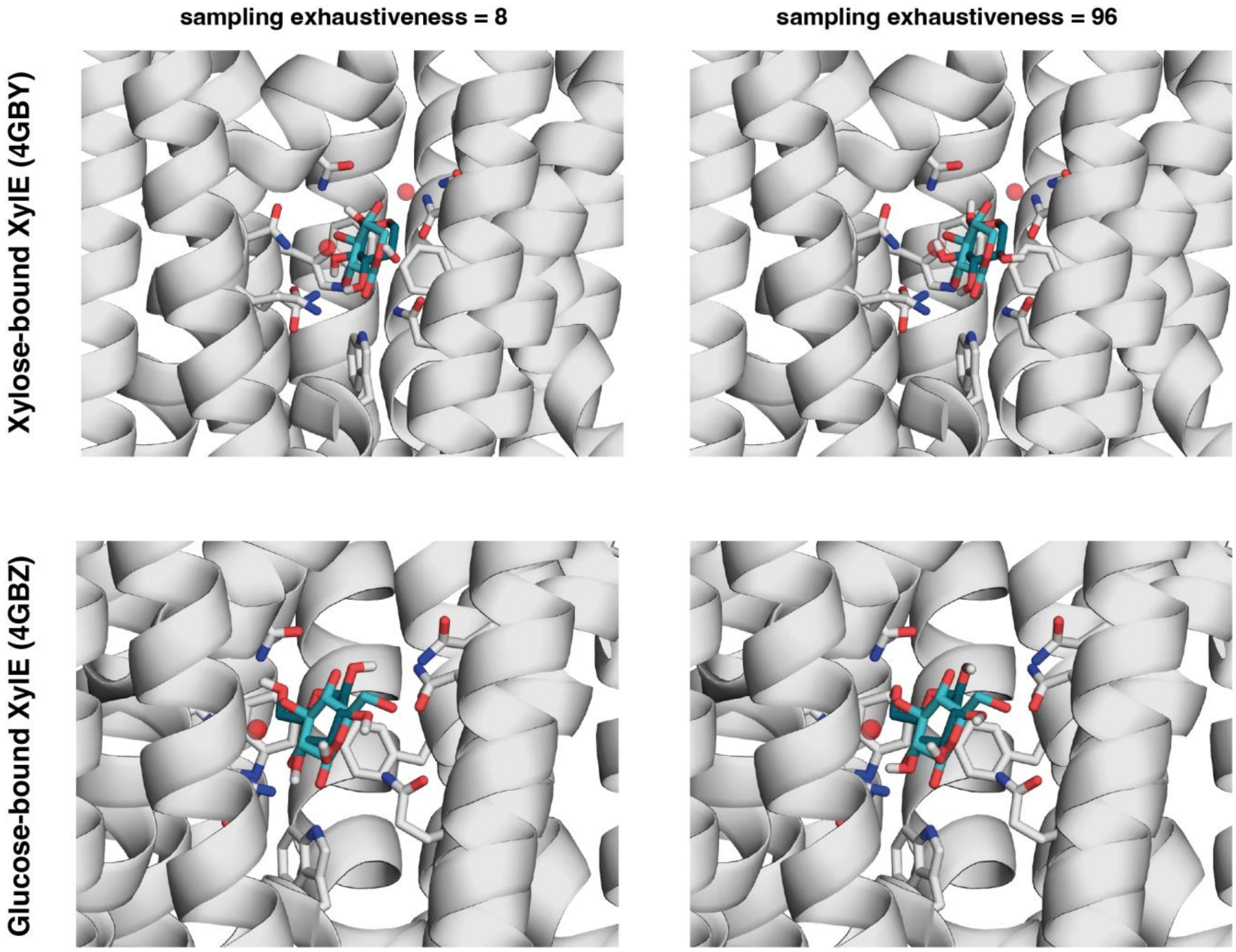

Figure S19 Evaluation of docking xylose/glucose to crystal structure using Autodock Vina. Two levels of exhaustiveness (8 and 96) were tested for docking xylose and glucose to their associated crystal structure (4GBY and 4GBZ). Crystal ligand structures were shown in cyan; the top pose from docking was shown in dark green. Binding site residues (F24, Q168, Q175, Q288, Q289, N294, N325, W392, Q415 and W416) were shown in white.

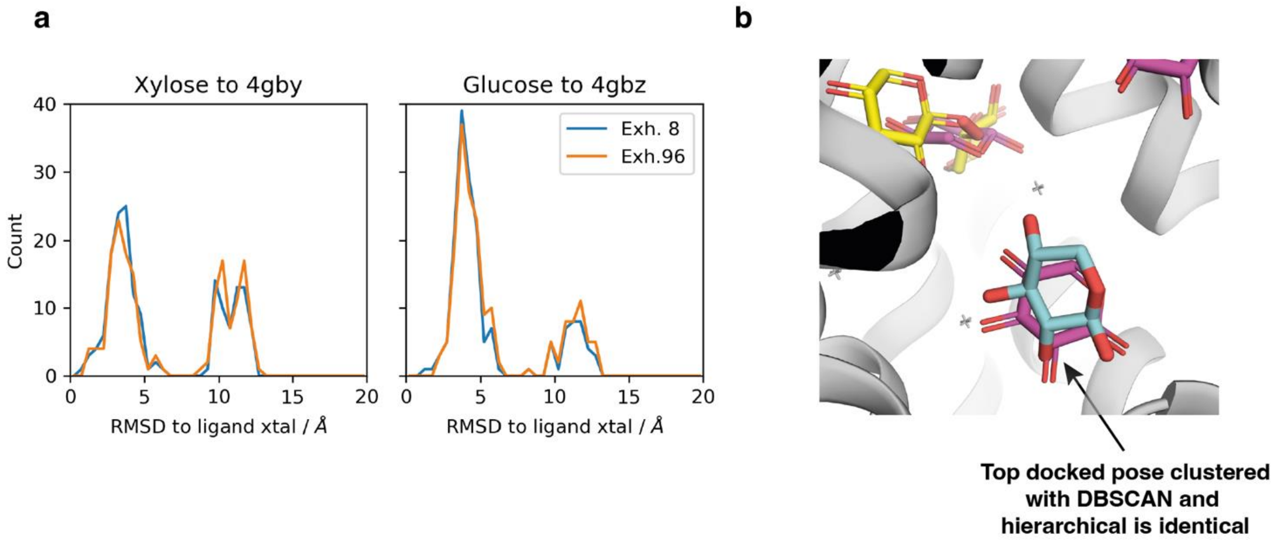

Figure S20 Docking analysis of xylose/glucose structures. (**a**) Distribution plots for rigid docking poses (xylose docked to XylE OF (4GBY) and glucose docked to XylE OF (4GBZ)). Each docking generated 9 poses, which led to 171 docked poses for 19 representative protein structures from MD simulations. Rigid docking was tested in two levels of exhaustiveness (8 and 96). (**b**) Representative poses from docking were clustered using DBSCAN and hierarchical clustering methods for rigid docking. Xylose pose from crystal structure was shown in cyan, docked xylose (clustered using DBSCAN) was shown in yellow, and docked xylose (clustered using hierarchical clustering method) was shown in magenta. the top docked pose in the binding was identical from DBSCAN and hierarchical clustering, with an RMSD value of 1.626 Å to ligand pose in the crystal structure.

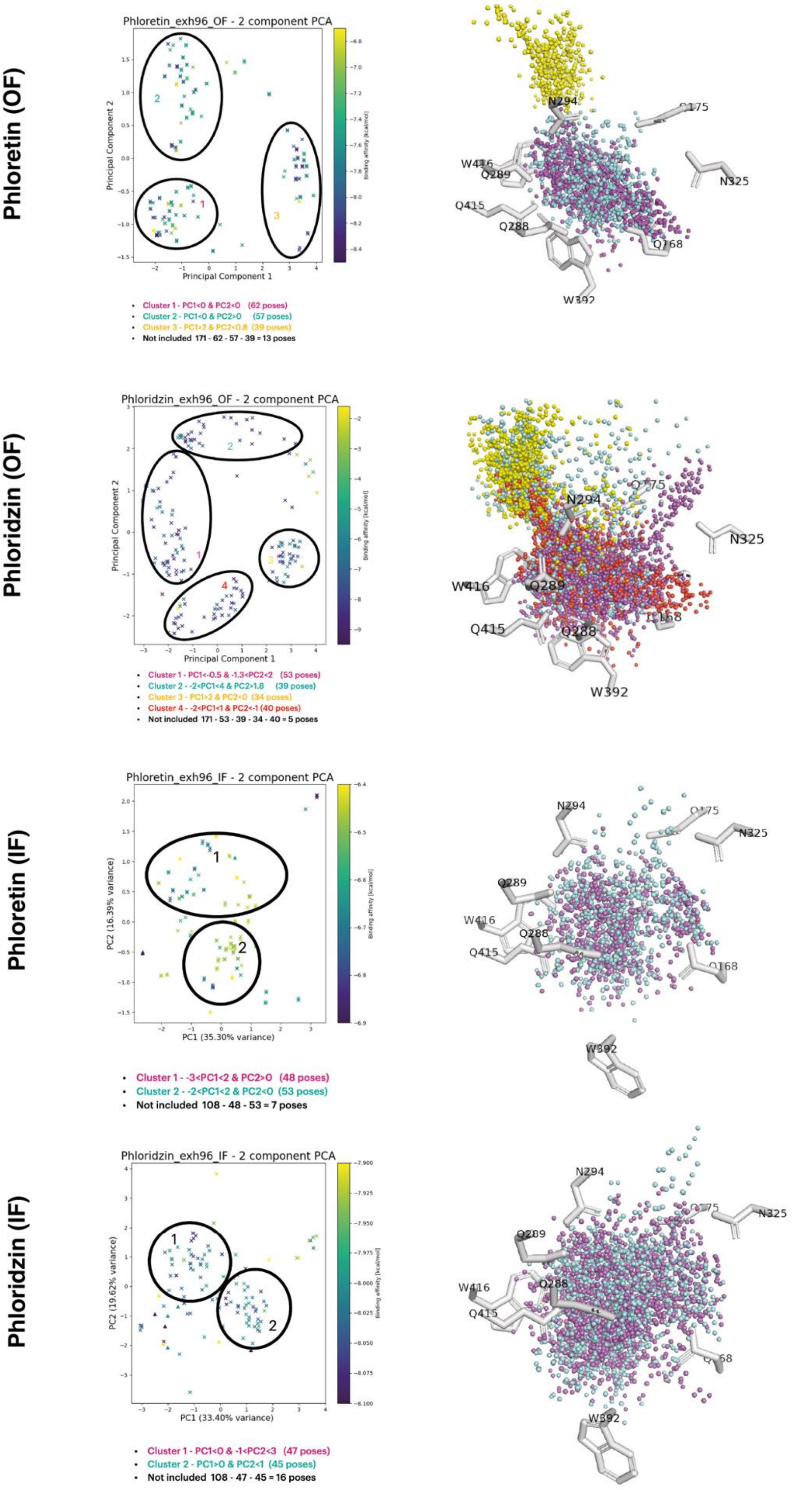

Phloretin (OF)
Phloretin_exh96_OF - 2 component PCA
Principal Component 1
Principal Component 2
Binding affinity [kcal/mol]
N294
Q175
W416
Q289
N325
Q415
Q288
Q168
W392
Cluster 1 - PC1<0 & PC2<0 (62 poses)
Cluster 2 - PC1<0 & PC2>0 (57 poses)
Cluster 3 - PC1>2 & PC2<0.8 (39 poses)
Not included 171 - 62 - 57 - 39 = 13 poses
Phloridzin (OF)
Phloridzin_exh96_OF - 2 component PCA
Principal Component 1
Principal Component 2
Binding affinity [kcal/mol]
N294
Q175
N325
W416
Q289
Q415
Q288
W392
Cluster 1 - PC1<-0.5 & -1.3<PC2<2 (53 poses)
Cluster 2 - -2<PC1<4 & PC2>1.8 (39 poses)
Cluster 3 - PC1>2 & PC2<0 (34 poses)
Cluster 4 - -2<PC1<1 & PC2<-1 (40 poses)
Not included 171 - 53 - 39 - 34 - 40 = 5 poses
Phloretin (IF)
Phloretin_exh96_IF - 2 component PCA
PC1 (35.30% variance)
PC2 (16.39% variance)
Binding affinity [kcal/mol]
N294
Q175
N325
Q289
W416
Q288
Q415
Q168
W392
Cluster 1 - -3<PC1<2 & PC2>0 (48 poses)
Cluster 2 - -2<PC1<2 & PC2<0 (53 poses)
Not included 108 - 48 - 53 = 7 poses
Phloridzin (IF)
Phloridzin_exh96_IF - 2 component PCA
PC1 (33.40% variance)
PC2 (19.62% variance)
Binding affinity [kcal/mol]
N294
Q175
N325
Q289
W416
Q288
Q415
W392
Cluster 1 - PC1<0 & -1<PC2<3 (47 poses)
Cluster 2 - PC1>0 & PC2<1 (45 poses)
Not included 108 - 47 - 45 = 16 poses

Figure S21 PCA analysis of docked poses for phloretin and phloridzin. Two-component PCA analysis was performed for docked poses of phloretin and phloridzin structures. PCA plots were generated using a custom python script. Clusters are colored in magenta, cyan, yellow, red and black for clusters 1, 2, 3, 4 and "not included", respectively. Binding site residues are shown as white sticks.

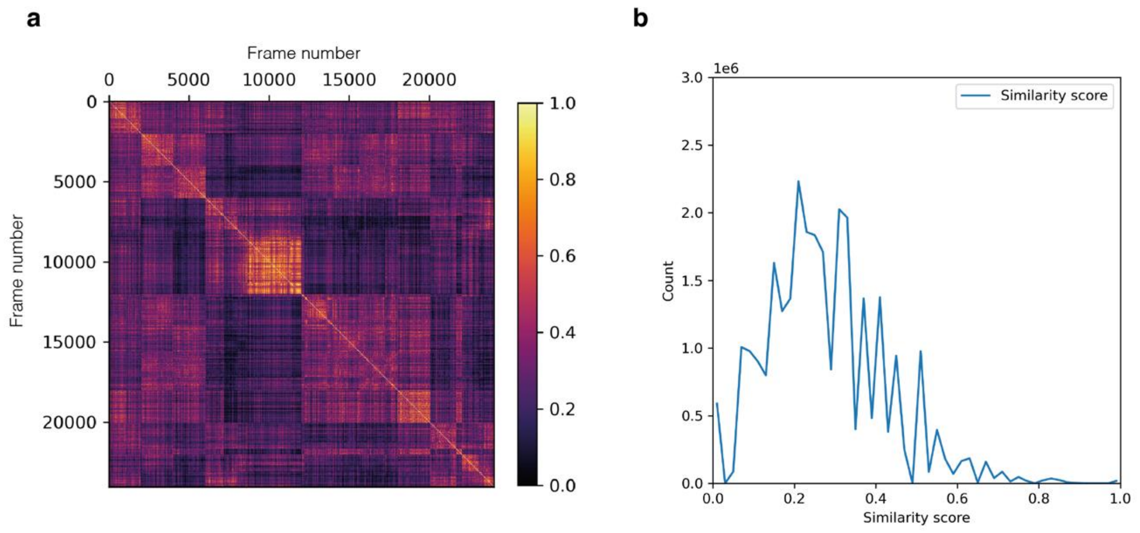

Figure S22 2D similarity score for protein-ligand interaction fingerprint analysis. (**a**) Pairwise Tanimoto coefficient of XylE-phloretin fingerprint (24012 MD frames) in OF conformation was calculated and plotted using a custom python script. Frames were coloured in gradient, yellow indicates a high similarity score, and black indicates a low similarity score. (**b**) Histogram of similarity score distribution for XylE-phloretin fingerprint (24012 MD frames) in OF conformation. The similarity score was displayed on the x-axis and the number of frames count ($1e^{6}$) was displayed on the y-axis.

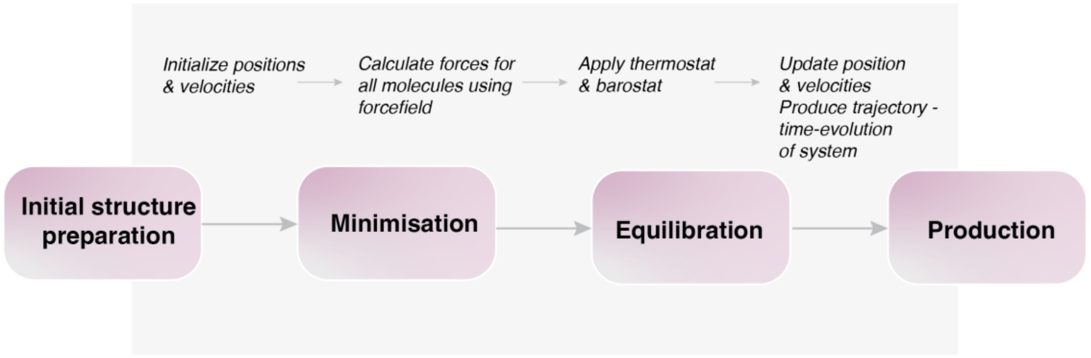

Figure S23 Workflow of a typical equilibrium MD simulation. The workflow starts from initial structure preparation and continues to minimisation (eliminate high energies in the model), equilibration (re-adjustment of particles to equilibrium) and production (data collection).

Supporting Tables

Table S1 Binding occupancy of XylE WT binding to xylose, glucose, phloretin and phloridzin.

	Kd[12] (µM)	Protein (µM)		Ligand (µM)		% Protein bound	
		Equilibration solution	Labelling solution	Equilibration solution	Labelling solution	Equilibration solution	Labelling solution
**Xylose**	350	13.86	1.37	30000.00	3000.0	98.85%	89.55%
**Glucose**	770	13.86	1.37	60000.00	6000.00	98.73%	88.62%
**Phloretin**	33.9	13.86	1.37	3000.00	300.0	98.88%	89.81%
**Phloridzin**	258	13.86	1.37	20000.00	2000.0	98.73%	88.57%

Table S2 HDX-MS measurements.

1. ΔHDX = (XylE WT+ Xylose) vs (XylE WT) in 10%DMSO

	XylE WT + Xylose	XylE WT
**HDX reaction details**	10mM potassium phosphate in H_2O pH 7.0, 0.02%DDM	
**HDX time course (min)**	0.5, 5, and 30 minutes	
**Number of peptides**	86	86
**Sequence Coverage**	82.7%	82.7%
**Average peptide length / Redundancy**	8.9 / 1.6	8.9 / 1.6
**Replicates (biological or technical)**	3 (technical)	3 (technical)
**Repeatability (average SD)**	0.046	0.043
**Significant differences in sum ΔHDX**	CI 99% ± 0.27 Da	

2. ΔHDX = (XylE WT+ Glucose) vs (XylE WT) in 10%DMSO

	XylE WT + Glucose	XylE WT
**HDX reaction details**	10mM potassium phosphate in H_2O pH 7.0, 0.02%DDM	
**HDX time course (min)**	0.5, 5, and 30 minutes	
**Number of peptides**	86	86
**Sequence Coverage**	82.7%	82.7%
**Average peptide length / Redundancy**	8.9 / 1.6	8.9 / 1.6
**Replicates (biological or technical)**	3 (technical)	3 (technical)
**Repeatability (average SD)**	0.044	0.043
**Significant differences in sum ΔHDX**	CI 99% ± 0.26 Da	

3. ΔHDX = (XylE WT+ Phloretin) vs (XylE WT) in 10%DMSO

	XylE WT + Phloretin	XylE WT
**HDX reaction details**	10mM potassium phosphate in H_2O pH 7.0, 0.02%DDM	
**HDX time course (min)**	0.5, 5, and 30 minutes	
**Number of peptides**	86	86
**Sequence Coverage**	82.7%	82.7%
**Average peptide length / Redundancy**	8.9 / 1.6	8.9 / 1.6
**Replicates (biological or technical)**	3 (technical)	3 (technical)
**Repeatability (average SD)**	0.044	0.043
**Significant differences in sum ΔHDX**	CI 99% ± 0.26 Da	

4. ΔHDX = (XylE WT+ Phloridzin) vs (XylE WT) in 10%DMSO

	XylE WT + Phloridzin	XylE WT
**HDX reaction details**	10mM potassium phosphate in H_2O pH 7.0, 0.02%DDM	
**HDX time course (min)**	0.5, 5, and 30 minutes	
**Number of peptides**	86	86
**Sequence Coverage**	82.7%	82.7%
**Average peptide length / Redundancy**	8.9 / 1.6	8.9 / 1.6
**Replicates (biological or technical)**	3 (technical)	3 (technical)
**Repeatability (average SD)**	0.044	0.043
**Significant differences in sum ΔHDX**	CI 99% ± 0.26 Da	

Table S3 HDX-MS reweighting data of mixing 2 ensemble structures.

Mix IF+OF (apo)	γ	Weighted RMSE to target	Applied work (kJ/mol)	Conformational population (IF:OF)
**Original**	3.5	0.177942	4.94481	0.72870:0.27130
**Omitting 66-88**	3.6	0.163209	5.01186	0.71076:0.28924
**Omitting 271-276**	4.6	0.171485	5.06270	0.74165:0.25835
**Omitting 66-88 + 271-276**	4.6	0.154807	4.95933	0.72677:0.27323

Mix IF+OF (xylose)	γ	Weighted RMSE to target	Applied work (kJ/mol)	Conformational population (IF:OF)
**Original**	4.2	0.174327	4.96328	0.61804:0.38196
**Omitting 66-88**	4.4	0.158985	5.06920	0.60683:0.39317
**Omitting 271-276**	5.5	0.164855	5.00366	0.43961:0.56039
**Omitting 66-88 + 271-276**	5.8	0.148635	5.05329	0.42037:0.57963

Mix IF+OF (glucose)	γ	Weighted RMSE to target	Applied work (kJ/mol)	Conformational population (IF:OF)
**Original**	4.7	0.144861	4.98085	0.44715:0.55285
**Omitting 66-88**	4.7	0.137190	4.95480	0.44391:0.55609
**Omitting 271-276**	6.5	0.128285	4.96765	0.14566:0.85434
**Omitting 66-88 + 271-276**	6.8	0.118516	5.04470	0.14327:0.85673

Mix IF+OF (Phloretin) Representative #1	γ	Weighted RMSE to target	Applied work (kJ/mol)	Conformational population (IF:OF)
**Original**	4.4	0.179977	5.03989	0.72698:0.27302
**Omitting 66-88**	4.5	0.167363	4.98114	0.77315:0.22685
**Omitting 271-276**	6.9	0.165217	5.04423	0.60736:0.39264
**Omitting 66-88 + 271-276**	7.2	0.150428	5.04090	0.66203:0.33797

Mix IF+OF (Phloretin) Representative #2	γ	Weighted RMSE to target	Applied work (kJ/mol)	Conformational population (IF:OF)
**Original**	4.9	0.181900	4.96968	0.83969:0.16031
**Omitting 66-88**	4.9	0.165580	4.99049	0.86608:0.13392
**Omitting 271-276**	5.7	0.165947	4.93941	0.83969:0.16031
**Omitting 66-88 + 271-276**	6.5	0.151093	4.97699	0.67538:0.32462

Mix IF+OF (Phloretin) Representative #1+2	γ	Weighted RMSE to target	Applied work (kJ/mol)	Conformational population (IF:OF)
**Original**	4.6	0.180121	4.98044	0.78256:0.21744
**Omitting 66-88**	4.7	0.165396	4.94515	0.82852:0.17148
**Omitting 271-276**	6.2	0.164322	5.04684	0.62753:0.37247
**Omitting 66-88 + 271-276**	6.7	0.149445	5.03621	0.67511:0.32489

Mix IF+OF (Phloridzin) Representative #1	γ	Weighted RMSE to target	Applied work (kJ/mol)	Conformational population (IF:OF)
**Original**	4.7	0.148382	5.04179	0.44563:0.55437
**Omitting 66-88**	4.7	0.138700	4.97599	0.46246:0.53754
**Omitting 271-276**	7.6	0.124186	4.95055	0.34598:0.65402
**Omitting 66-88 + 271-276**	8.5	0.116034	5.04881	0.37590:0.62410

Mix IF+OF (Phloridzin) Representative #2	γ	Weighted RMSE to target	Applied work (kJ/mol)	Conformational population (IF:OF)
**Original**	5.3	0.154079	4.95580	0.45107:0.54893
**Omitting 66-88**	5.6	0.142263	5.05621	0.46646:0.53354
**Omitting 271-276**	9.2	0.132222	5.03193	0.38013:0.61987
**Omitting 66-88 + 271-276**	9.7	0.116802	5.04263	0.39750:0.60250

Mix IF+OF (Phloridzin) Representative #1+2	γ	Weighted RMSE to target	Applied work (kJ/mol)	Conformational population (IF:OF)
**Original**	4.8	0.149978	4.98607	0.45319:0.54681
**Omitting 66-88**	4.9	0.139437	4.97173	0.46834:0.53166
**Omitting 271-276**	8.6	0.1225890	5.01158	0.36350:0.63650
**Omitting 66-88 + 271-276**	9.4	0.115578	4.99555	0.38712:0.61288

Table S4 HDX-MS reweighting experiments of mixing 10 ensemble structures.

Mix 10 to fit apo	γ	Weighted RMSE to target	Applied work (kJ/mol)	Conformational population (IF:OF)
**Original**	3.8	0.167557	5.07162	0.58981:0.41019
**Omitting 66-88**	4.0	0.156539	4.97036	0.62006:0.37994
**Omitting 271-276**	5.1	0.152565	4.95515	0.44674:0.55326
**Omitting 66-88 + 271-276**	5.6	0.140929	5.00836	0.49493:0.50307

Mix 10 to fit xylose	γ	Weighted RMSE to target	Applied work (kJ/mol)	Conformational population (IF:OF)
**Original**	3.7	0.163952	4.92331	0.49399:0.50601
**Omitting 66-88**	4.0	0.153689	4.98495	0.51704:0.48296
**Omitting 271-276**	5.0	0.150334	5.06030	0.29957:0.70043
**Omitting 66-88 + 271-276**	5.4	0.141076	5.02943	0.34622:0.65378

Mix 10 to fit glucose	γ	Weighted RMSE to target	Applied work (kJ/mol)	Conformational population (IF:OF)
**Original**	4.2	0.140658	5.07313	0.32059:0.67941
**Omitting 66-88**	4.3	0.136612	5.02650	0.32732:0.67268
**Omitting 271-276**	5.4	0.121775	4.96685	0.11498:0,88502
**Omitting 66-88 + 271-276**	5.8	0.118668	5.01107	0.12321:0.87679

Mix 10 to fit phloretin	γ	Weighted RMSE to target	Applied work (kJ/mol)	Conformational population (IF:OF)
**Original**	3.6	0.179290	4.95745	0.73512:0.26488
**Omitting 66-88**	3.8	0.164210	5.01612	0.76382:0.23618
**Omitting 271-276**	5.2	0.164627	5.01602	0.65402:0.34598
**Omitting 66-88 + 271-276**	5.6	0.148582	5.02416	0.69496:0.34504

Mix 10 to fit phloridzin	γ	Weighted RMSE to target	Applied work (kJ/mol)	Conformational population (IF:OF)
**Original**	4.5	0.129704	4.96615	0.68902:0.31098
**Omitting 66-88**	4.7	0.117545	5.04124	0.71142:0.28858
**Omitting 271-276**	7.5	0.110742	4.96434	0.56190:0.43810
**Omitting 66-88 + 271-276**	8.5	0.098408	5.03106	0.60442:0.39558

Table S5 RMSE value of peptides after reweighting.

	Peptides with RMSE over 0.3	Common peptides with RMSE over 0.3	Unique peptides with RMSE over 0.3	Union peptides with RMSE over 0.3
**Mix IF+OF (apo)**	10-15 (0.35606); 49-56 (0.32942); 271-276(0.45235); 305-315(0.32282); 435-445(0.33012); 436-444 (0.37610)	271-276	10-15; 49-56; 305-315; 435-445; 436-444	10-15; 43-48; 49-56; 227-240; 241-248; 271-276; 298-304; 300-305; 305-315; 393-409; 395-409; 435-445; 436-444
**Mix IF+OF (xylose)**	10-15(0.32147; 49-56(0.36862); 241-248(0.33938); 271-276(0.43909); 300-305(0.30507);		10-15; 49-56; 241-248; 300-305	
**Mix IF+OF (glucose)**	49-56(0.33397); 271-276(0.53095); 298-304(0.32483); 300-305(0.35711);		49-56; 298-304; 300-305	
**Mix IF+OF (phloretin)**	10-15(0.39923); 43-48(0.34699); 241-248(0.39807); 271-276(0.56135); 393-409(0.33052); 395-409(0.30392)		10-15; 43-48; 241-248; 393-409;395-409	
**Mix IF+OF (phloridzin)**	43-48 (0.33259); 227-240(0.32147); 241-248(0.33703); 271-276(0.62760);		43-48; 227-240; 241-248	

Table S6 HDX-MS reweighting experiments of mixing 14 ensemble structures.

Mix 14 to fit apo	γ	Weighted RMSE to target	Applied work (kJ/mol)	Conformational population (IF:OF)
**Original**	3.7	0.167289	5.04337	0.61204:0.38796
**Omitting 66-88**	3.9	0.154267	5.06030	0.65093:0.34907
**Omitting 271-276**	5.0	0.151724	5.02817	0.45409:0.54591
**Omitting 66-88 + 271-276**	5.3	0.138593	5.00156	0.51161:0.48839

Mix 14 to fit xylose	γ	Weighted RMSE to target	Applied work (kJ/mol)	Conformational population (IF:OF)
**Original**	3.6	0.163218	4.98732	0.50212:0.49788
**Omitting 66-88**	3.8	0.152143	4.93235	0.53420:0.46580
**Omitting 271-276**	4.9	0.149907	5.05656	0.29938:0.70062
**Omitting 66-88 + 271-276**	5.2	0.139319	4.99465	0.37072:0.62928

Mix 14 to fit glucose	γ	Weighted RMSE to target	Applied work (kJ/mol)	Conformational population (IF:OF)
**Original**	3.9	0.139824	4.95374	0.34900:0.65100
**Omitting 66-88**	4.0	0.135521	4.97450	0.36545:0.63455
**Omitting 271-276**	5.6	0.121471	5.02286	0.12684:0.87316
**Omitting 66-88 + 271-276**	5.9	0.117855	5.01597	0.15196:0.84804

Mix 14 to fit phloretin	γ	Weighted RMSE to target	Applied work (kJ/mol)	Conformational population (IF:OF)
**Original**	3.7	0.179584	5.07340	0.75874:0.24126
**Omitting 66-88**	3.8	0.164504	4.94549	0.78855:0.21145
**Omitting 271-276**	5.2	0.165276	4.99458	0.65387:0.34613
**Omitting 66-88 + 271-276**	5.6	0.149153	4.95957	0.69457:0.30543

Mix 14 to fit phloridzin	gamma	Weighted RMSE to target	Applied work (kJ/mol)	Conformational population (IF:OF)
**Original**	4.6	0.129659	5.01028	0.72563:0.27437
**Omitting 66-88**	4.8	0.116872	5.05214	0.75229:0.24771
**Omitting 271-276**	7.5	0.110742	5.00505	0.56482:0.43518
**Omitting 66-88 +271-276**	8.4	0.098166	4.98115	0.60631:0.39369

Table S7 HDX-MS data comparison between previously published and newly generated data for XylE WT. MaxD experiments for newly generated data were performed in parallel with the new HDX labelling, but not for previously published data.

start	end	Uptake (5 min) Previous data	Uptake (5 min) New data	Uptake (30 min) Previous data	Uptake (30 min) New data	MaxD For previous data	MaxD For new data
10	15	0.988023	1.341604	1.648423	2.178138	3.381621	3.138548
16	23	0.036203	0.02243	-0.04094	0.050032	4.731473	4.337314
29	36	0.360692	1.983312	0.712998	2.599542	5.022441	4.302648
29	38	0.47115	2.554559	1.029097	3.625274	6.525283	5.759576
31	38	0.42444	1.677353	1.06695	2.609273	4.518544	4.027515
41	48	0.904589	1.864013	1.302296	2.474225	4.039788	3.752867
43	48	0.82694	1.597494	1.21237	1.940894	2.835552	2.664079
49	56	1.026593	2.66125	1.03743	3.597548	4.591334	4.115131
68	88	3.383246	3.761676	4.186496	4.558251	9.930536	10.58284
110	122	3.891694	5.321523	5.397606	6.291501	6.913373	6.430672
110	124	4.028711	5.820736	5.891508	7.021107	8.349839	7.944533
122	129	0.161974	0.501083	0.294865	0.817584	3.950091	3.385067
143	150	0.506214	0.731701	1.091809	1.460049	3.949765	3.422853
146	150	0.447891	0.611255	0.805693	0.963076	1.868529	1.573661
164	169	0.576433	0.863486	1.101133	1.394248	3.424384	2.873047
169	173	0.052192	0.048571	0.095737	0.092673	2.900675	2.574969
217	226	2.413823	2.737934	2.843405	3.250789	5.307931	4.237368
227	240	6.632008	6.57535	6.600157	6.749862	8.180742	6.843217
241	248	3.545824	3.652138	3.82323	3.822171	4.69903	4.176144
298	304	0.051406	0.649032	0.06824	1.495712	3.287716	2.846126
344	348	0.015812	0.067183	0.018998	0.109736	2.837236	2.651964
395	409	2.281486	2.072208	3.36335	4.056172	9.301031	7.905648

Table S8 Modification of residue protonation states and Histidine sidechain flips using MolProbity[13]

**Protonation states**	**E206 = neutral**				
**Sidechain flips**	HSE158	HSE258	HSD262	HSD438	HSE440

Table S9 Energy minimisation and equilibration protocol for XylE protein structure.

	Length(ns)	Timestep(ps)	Ensemble	Restraint location	Restraint value (kJ mol^{-1} nm^{-2})	Lipid restraint location	Lipid restraint value (kJ mol^{-1} nm^{-2})	Thermostat/barostat
min	10k steps	N/A	N/A	None	N/A	N/A	N/A	N/A
Eq1a	10	0.001	NVT	All protein atoms	4000	Phosphorus atom positional restraint in the z-direction; dihedral restraints for palmitoyl chirality	Positional:1000; Dihedral:1000	V-rescale thermostat
Eq1b	10	0.002	NPT	All protein atoms	4000	Phosphorus atom positional restraint in the z-direction; dihedral restraints for palmitoyl chirality	Positional:40; Dihedral:100	V-rescale thermostat; Berendsen barostat
Eq2	5	0.002	NPT	All protein atoms	4000	N/A	N/A	V-rescale thermostat; Berendsen barostat
Eq3	5	0.002	NPT	Protein backbone	1000	N/A	N/A	V-rescale thermostat; Berendsen barostat
Eq4	5	0.002	NPT	Protein backbone	200	N/A	N/A	V-rescale thermostat; Berendsen barostat
Eq5	5	0.002	NPT	Protein CA	20	N/A	N/A	V-rescale thermostat; Berendsen barostat
Eq6	60	0.002	NPT	None	N/A	N/A	N/A	Nose-Hoover thermostat; Parrinello-Rahman barostat

Table S10 Energy minimisation and equilibration for XylE-ligand bound structure. Solute_all represents all protein and ligand atoms. Solute_bb is for all protein backbone atoms, Solute_sc is all protein sidechain atoms and ligand atoms, and Solute_ca is all protein alpha carbon atoms. Lipid_headgroup is the POPE phosphorus atom and lipid_dihedral represents the C1-C3-C2-O21 and C28-C29-C210-C211 dihedrals in POPE.

	**Length(ns)**	**Timestep(ps)**	**Ensemble**	**Restraint location & value ((kJ mol^{-1} nm^{-2}))**	**Thermostat/barostat**
**min**	10k steps	N/A	N/A	None	N/A
**Eq1a**	10	0.001	NVT	Solute_all = 4000; Lipid_headgroup=1000; Lipid_dihedral = 1000	V-rescale thermostat
**Eq1b**	10	0.002	NPT	Solute_all = 4000; Lipid_headgroup=40; Lipid_dihedral = 100	V-rescale thermostat; Berendsen barostat
**Eq2**	5	0.002	NPT	Solute_all = 4000	V-rescale thermostat; Berendsen barostat
**Eq3**	5	0.002	NPT	Solute_bb = 1000; solute_sc = 0	V-rescale thermostat; Berendsen barostat
**Eq4**	5	0.002	NPT	Solute_bb = 200; solute_sc = 0	V-rescale thermostat; Berendsen barostat
**Eq5**	5	0.002	NPT	Solute_ca=20	V-rescale thermostat; Berendsen barostat
**Eq6**	60	0.002	NPT	None	Nose-Hoover thermostat; Parrinello-Rahman barostat

Table S11 Representative XylE-inhibitor bound structures selection.

		Phloretin		Phloridzin	
		OF	IF	OF	IF
SD_RMSD (nm)	Representative structure #1	0.04845	0.04166	0.04451	0.03791
	Representative structure #2	0.05323	0.08742	0.04739	0.06654
Number of visited starting structures	Representative structure #1	4/4	4/4	4/4	4/4
	Representative structure #2	4/4	4/4	4/4	4/4
Population of total frames	Representative structure #1	3501/24012 = 14.58%	3811/24012 = 15.87%	5743/24012 = 23.92%	5955/24012 =24.80%
	Representative structure #2	4601/24012 = 19.16%	2999/24012 = 12.49%	4021/24012 =16.75%	4140/24012 = 17.24%
The population of frames inside the cluster shares < 1.5 Å RMSD with the average structure	Representative structure #1	3247/3501 = 92.74%	3771/3811 = 98.95%	5688/5743 = 99.04%	5903/5955 = 99.13%
	Representative structure #2	4295/4601 = 93.35%	2778/2999 = 92.63%	3825/4021 = 95.12%	4091/4140 = 98.82%

Supporting References

1. Lau, A. M.; Claesen, J.; Hansen, K.; Politis, A., Deuteros 2.0: Peptide-level significance testing of data from hydrogen deuterium exchange mass spectrometry. *Bioinformatics* **2021,** *37* (2), 270-272.
2. Clarke, J.; Hounslow, A. M.; Bycroft, M.; Fersht, A. R., Local breathing and global unfolding in hydrogen exchange of barnase and its relationship to protein folding pathways. *Proc Natl Acad Sci U S A* **1993,** *90* (21), 9837-41.
3. Milne, J. S.; Mayne, L.; Roder, H.; Wand, A. J.; Englander, S. W., Determinants of protein hydrogen exchange studied in equine cytochrome c. *Protein Sci* **1998,** *7* (3), 739-45.
4. Loh, S. N.; Prehoda, K. E.; Wang, J.; Markley, J. L., Hydrogen exchange in unligated and ligated staphylococcal nuclease. *Biochemistry* **1993,** *32* (41), 11022-8.
5. Chamberlain, A. K.; Handel, T. M.; Marqusee, S., Detection of rare partially folded molecules in equilibrium with the native conformation of RNaseH. *Nat Struct Biol* **1996,** *3* (9), 782-7.
6. Morozova-Roche, L. A.; Arico-Muendel, C. C.; Haynie, D. T.; Emelyanenko, V. I.; Van Dael, H.; Dobson, C. M., Structural characterisation and comparison of the native and A-states of equine lysozyme. *J Mol Biol* **1997,** *268* (5), 903-21.
7. Schulman, B. A.; Redfield, C.; Peng, Z. Y.; Dobson, C. M.; Kim, P. S., Different subdomains are most protected from hydrogen exchange in the molten globule and native states of human alpha-lactalbumin. *J Mol Biol* **1995,** *253* (5), 651-7.
8. Kim, K. S.; Fuchs, J. A.; Woodward, C. K., Hydrogen exchange identifies native-state motional domains important in protein folding. *Biochemistry* **1993,** *32* (37), 9600-8.
9. Maier, J. A.; Martinez, C.; Kasavajhala, K.; Wickstrom, L.; Hauser, K. E.; Simmerling, C., ff14SB: Improving the Accuracy of Protein Side Chain and Backbone Parameters from ff99SB. *J Chem Theory Comput* **2015,** *11* (8), 3696-713.
10. Jorgensen, W. L.; Chandrasekhar, J.; Madura, J. D., Comparison of simple potential functions for simulating liquid water. *The Journal of Chemical Physics* **1983,** *79* (2), 926-935.
11. Eswar, N.; Webb, B.; Marti-Renom, M. A.; Madhusudhan, M. S.; Eramian, D.; Shen, M. Y.; Pieper, U.; Sali, A., Comparative protein structure modeling using Modeller. *Curr Protoc Bioinformatics* **2006,** *Chapter 5*, Unit-5 6.
12. Jiang, X.; Wu, J.; Ke, M.; Zhang, S.; Yuan, Y.; Lin, J. Y.; Yan, N., Engineered XylE as a tool for mechanistic investigation and ligand discovery of the glucose transporters GLUTs. *Cell Discov* **2019,** *5*, 14.
13. Williams, C. J.; Headd, J. J.; Moriarty, N. W.; Prisant, M. G.; Videau, L. L.; Deis, L. N.; Verma, V.; Keedy, D. A.; Hintze, B. J.; Chen, V. B.; Jain, S.; Lewis, S. M.; Arendall, W. B., 3rd; Snoeyink, J.; Adams, P. D.; Lovell, S. C.; Richardson, J. S.; Richardson, D. C., MolProbity: More and better reference data for improved all-atom structure validation. *Protein Sci* **2018,** *27* (1), 293-315.
14. Lee, J.; Cheng, X.; Swails, J. M.; Yeom, M. S.; Eastman, P. K.; Lemkul, J. A.; Wei, S.; Buckner, J.; Jeong, J. C.; Qi, Y.; Jo, S.; Pande, V. S.; Case, D. A.; Brooks, C. L., 3rd; MacKerell, A. D., Jr.; Klauda, J. B.; Im, W., CHARMM-GUI Input Generator for NAMD, GROMACS, AMBER, OpenMM, and CHARMM/OpenMM Simulations Using the CHARMM36 Additive Force Field. *J Chem Theory Comput* **2016,** *12* (1), 405-13.

15. Wu, E. L.; Cheng, X.; Jo, S.; Rui, H.; Song, K. C.; Davila-Contreras, E. M.; Qi, Y.; Lee, J.; Monje-Galvan, V.; Venable, R. M.; Klauda, J. B.; Im, W., CHARMM-GUI Membrane Builder toward realistic biological membrane simulations. *J Comput Chem* **2014,** *35* (27), 1997-2004.
16. Lomize, M. A.; Pogozheva, I. D.; Joo, H.; Mosberg, H. I.; Lomize, A. L., OPM database and PPM web server: resources for positioning of proteins in membranes. *Nucleic Acids Res* **2012,** *40* (Database issue), D370-6.
17. Gordon, J. C.; Myers, J. B.; Folta, T.; Shoja, V.; Heath, L. S.; Onufriev, A., H++: a server for estimating pKas and adding missing hydrogens to macromolecules. *Nucleic Acids Res* **2005,** *33* (Web Server issue), W368-71.
18. Parrinello, M.; Rahman, A., Polymorphic transitions in single crystals: A new molecular dynamics method. *Journal of Applied Physics* **1981,** *52* (12), 7182.
19. Kim, S.; Chen, J.; Cheng, T.; Gindulyte, A.; He, J.; He, S.; Li, Q.; Shoemaker, B. A.; Thiessen, P. A.; Yu, B.; Zaslavsky, L.; Zhang, J.; Bolton, E. E., PubChem in 2021: new data content and improved web interfaces. *Nucleic Acids Res* **2021,** *49* (D1), D1388-D1395.
20. O'Boyle, N. M.; Banck, M.; James, C. A.; Morley, C.; Vandermeersch, T.; Hutchison, G. R., Open Babel: An open chemical toolbox. *J Cheminform* **2011,** *3*, 33.
21. Vanommeslaeghe, K.; MacKerell, A. D., Jr., Automation of the CHARMM General Force Field (CGenFF) I: bond perception and atom typing. *J Chem Inf Model* **2012,** *52* (12), 3144-54.
22. Vanommeslaeghe, K.; Raman, E. P.; MacKerell, A. D., Jr., Automation of the CHARMM General Force Field (CGenFF) II: assignment of bonded parameters and partial atomic charges. *J Chem Inf Model* **2012,** *52* (12), 3155-68.
23. Vanommeslaeghe, K.; Hatcher, E.; Acharya, C.; Kundu, S.; Zhong, S.; Shim, J.; Darian, E.; Guvench, O.; Lopes, P.; Vorobyov, I.; Mackerell, A. D., Jr., CHARMM general force field: A force field for drug-like molecules compatible with the CHARMM all-atom additive biological force fields. *J Comput Chem* **2010,** *31* (4), 671-90.
24. M. J. Frisch, G. W. T., H. B. Schlegel, G. E. Scuseria, M. A. Robb, J. R. Cheeseman, G. Scalmani, V. Barone, B. Mennucci, G. A. Petersson, H. Nakatsuji, M. Caricato, X. Li, H. P. Hratchian, A. F. Izmaylov, J. Bloino, G. Zheng, J. L. Sonnenberg, M. Hada, M. Ehara, K. Toyota, R. Fukuda, J. Hasegawa, M. Ishida, T. Nakajima, Y. Honda, O. Kitao, H. Nakai, T. Vreven, J. A. Montgomery, Jr., J. E. Peralta, F. Ogliaro, M. Bearpark, J. J. Heyd, E. Brothers, K. N. Kudin, V. N. Staroverov, R. Kobayashi, J. Normand, K. Raghavachari, A. Rendell, J. C. Burant, S. S. Iyengar, J. Tomasi, M. Cossi, N. Rega, J. M. Millam, M. Klene, J. E. Knox, J. B. Cross, V. Bakken, C. Adamo, J. Jaramillo, R. Gomperts, R. E. Stratmann, O. Yazyev, A. J. Austin, R. Cammi, C. Pomelli, J. W. Ochterski, R. L. Martin, K. Morokuma, V. G. Zakrzewski, G. A. Voth, P. Salvador, J. J. Dannenberg, S. Dapprich, A. D. Daniels, Ö. Farkas, J. B. Foresman, J. V. Ortiz, J. Cioslowski, and D. J. Fox, Gaussian 09 **2009,** *Gaussian, Inc., Wallingford CT*.
25. Mayne, C. G.; Saam, J.; Schulten, K.; Tajkhorshid, E.; Gumbart, J. C., Rapid parameterization of small molecules using the Force Field Toolkit. *J Comput Chem* **2013,** *34* (32), 2757-70.
26. Pang, Y. T.; Pavlova, A.; Tajkhorshid, E.; Gumbart, J. C., Parameterization of a drug molecule with a halogen sigma-hole particle using ffTK: Implementation, testing, and comparison. *J Chem Phys* **2020,** *153* (16), 164104.

27. Wojcikowski, M.; Zielenkiewicz, P.; Siedlecki, P., Open Drug Discovery Toolkit (ODDT): a new open-source player in the drug discovery field. *J Cheminform* **2015,** *7*, 26.
28. Best, R. B.; Vendruscolo, M., Structural interpretation of hydrogen exchange protection factors in proteins: characterization of the native state fluctuations of CI2. *Structure* **2006,** *14* (1), 97-106.
29. Nguyen, D.; Mayne, L.; Phillips, M. C.; Walter Englander, S., Reference Parameters for Protein Hydrogen Exchange Rates. *J Am Soc Mass Spectrom* **2018,** *29* (9), 1936-1939.
30. Laskowski, R. A.; Swindells, M. B., LigPlot+: multiple ligand-protein interaction diagrams for drug discovery. *J Chem Inf Model* **2011,** *51* (10), 2778-86.
